# Supplementary material for: Data-driven design of molecular nanomagnets
Source: Nat Commun. 2022 Dec 9;13:7626. doi: 10.1038/s41467-022-35336-9 (PMC9734471; doi:10.1038/s41467-022-35336-9)
Supplement: Supplementary file 1 — Supplementary Information [file 41467_2022_35336_MOESM1_ESM.pdf]

Supplementary Information

Data-driven design of molecular nanomagnets

Yan Duan[†,1,2], Lorena E. Rosaleny[†,*,1], Joana T. Coutinho[†,*,1,3], Silvia Giménez-Santamarina[1], Allen Scheie[4], José J. Baldoví[1], Salvador Cardona-Serra[1], Alejandro Gaita-Ariño[*,1]

[1] Instituto de Ciencia Molecular (ICMol), Universitat de València, C/ Catedrático José Beltrán 2, 46980 Paterna, Spain

[2] Spin-X Institute, South China University of Technology, Guangzhou 510641, P. R. China

[3] Centre for Rapid and Sustainable Product Development, Polytechnic of Leiria, 2430-028 Marinha Grande, Portugal

[4] Neutron Scattering Division, Oak Ridge National Laboratory, Oak Ridge, Tennessee 37831, USA

Supplementary Methods: Supplementary Sections 1-2

Supplementary Section 1. Construction of the dataset **3**

1.1 The question of publication bias 8

Supplementary Section 2. Classification in chemical families **10**

2.1. LnPc2 family 11

2.2. POM family 12

2.3. Schiff base family 13

2.4. Metallocene family 14

2.5. Diketonate family 15

2.6. Radical family 16

2.7. TM near Ln family 17

2.8. Mixed ligands family 18

2.9. Other families 19

Supplementary Discussion: Supplementary Sections 3-10

Supplementary Section 3. A graphical, interactive, browsable App **20**

3.1. Gallery of graphs: chemical variables to optimise the physical properties 23

3.2. Extended gallery of SIMDAVIS graphs: Arrhenius equation parameters 34

Supplementary Section 4. Statistical analysis of the chemical variables **36**

4.1. Initial multiple correspondence analysis 36

4.2. Clustering studies for the chemical variables 40

4.3. Lognormal modelling 45

Supplementary Section 5. Statistical analysis of the physical variables **45**

5.1. Overview of the main statistical relationships 46

5.2. Simple frequency distributions 48

5.3. Correlations between physical variables 48

5.4 The question of U_{eff} vs $U_{eff,ff}$ 53

5.5 Dependence of τ_0 vs U_{eff} 55

Supplementary Section 6. FAMD and magnetostructural clustering **59**

6.1. Magnetostructural clusters 61

Supplementary Section 7. Extended SHAPE analysis, comparison with reference polyhedra **66**

7.1 Methodology 66

7.2 Results 67

Supplementary Section 8. Extended SHAPE analysis, axial distortions **72**
8.1 Methodology 72
8.2 Results 75

Supplementary Section 9. Extended figures and discussion about U_{eff} vs Raman vs QTM **77**
9.1 Investigation of the relation between different relaxation parameters 77
9.2 Influence of CN and the number of ligands to optimise Raman and U_{eff} 82
9.3 Linear relation between log($U_{eff,ff}$) vs log(C) 84
9.4 Linear relation between log($U_{eff,ff}$) vs log(n) 91
9.5 Linear relation between log($U_{eff,ff}$) vs log(τ QTM) 97

Supplementary Section 10. Evidence for/against the main tested hypotheses **99**

Supplementary References **100**

Supplementary Section 1. Construction of the dataset

Data extraction was restricted to variables that can be systematically extracted from articles included in the present study. Our objectives for the data analysis were twofold: the correlation between different variables of the same (physical or chemical) category and the correlation between two variables from different (chemical and physical) categories. In the first case, the goal is to determine whether the variables are closely correlated with each other, and thus to simplify our analysis and to avoid false correlations. In the second case, the goal is to determine which chemical variables are proven to be the most influential on the physical performance.

For physical variables, we focus on the magnetic hysteresis and the behaviour of the out-of-phase component of the ac susceptibility. These two kinds of experimental observations are the most basic experimental tell-tale signs for SIM behaviour. For both, we extract from the articles qualitative and quantitative information. For ac susceptibility, we extract as qualitative information whether the out-of-phase component of the ac susceptibility χ'' vs the temperature has a maximum when there is no external dc magnetic field, or whether this maximum is absent but there is a frequency-dependent behaviour of χ'' with the temperature. As quantitative information, we extract the temperature of said χ'' vs T maximum. If the maximum of the out-of-phase component of the ac susceptibility appears in the presence of an external dc magnetic field, we extract the temperature at which the said maximum value appears and the applied external field. From reported magnetisation vs the magnetic field experiments, we extract as qualitative information the presence of full hysteresis (with remnant magnetisation and/or a coercive field), or at least a pinched (butterfly) hysteresis, and as quantitative information the maximum hysteresis temperature reported. In addition, a series of variables from the more extended theoretical analysis of the experimental data, namely the effective energy barrier U_{eff} and the relaxation time τ_0, as well as relevant information on what kind of fit gave rise to these parameters are also included.

In the case of the chemical variables, the information we collected and analysed is as follows: (a) the chemical family of the complex; (b) the lanthanide (Ln) ion; (c) whether the Ln ion is oblate, prolate or isotropic; (d) whether the Ln ion is Kramers or not; (e) the concentration of the sample (if the diamagnetic dilution is studied); (f) the coordination number of the Ln ion; (g) the number of coordinated ligands; (h) the coordination elements.

A full list of the variables, including the number of data points and the percentage of samples with valid values for each variable in the dataset, can be found in Supplementary Figs. 1.1 and 1.2. As a consequence of this sparseness in the data, not all samples will be present in all graphs: any (x vs y) plot can only include samples for which x and y are simultaneously present in the dataset.

Chemical Variables	N
Chemical Family	1411
Ln ion	1411
Ln anisotropy	1411
Ln Kramers	1411
Coordination Number	1337
Number of Ligands	1379
Coordination Elements	1411
Concentration	1402
Closest Polyhedron	795
CSM	795
Axial Distortion	622
Molecular Cluster	1404

Physical Variables	N
χ_{max}'' : Presence of maximum in $\chi''(T)$	1215
T_{B3} : Temperature of χ_{max}'' at zero-field	251
T_{B3H} : Temperature of χ_{max}'' at field H	347
H : External Applied Magnetic Field	348
Fit : U_{eff} fitted by T_{B3} or Argand	472
U_{eff} : Effective barrier with Orbach process	668
U_{eff2} : U_{eff} for a second relaxation process	24
$U_{eff,ff}$: U_{eff} for all processes (full fit)	76
τ_0 : Attempt time with Orbach process	621
$\tau_{0,ff}$: Attempt time with all relaxation processes	68
C : Raman prefactor	54
n : Raman exponent	56
τ_{QTM} : Quantum tunneling of the magnetisation time	41
$Hyst$: Presence of pinched or full hysteresis	380
T_{hyst} : Maximum hysteresis temperature	283
Mag. Struct.Cluster : Clustering by chem. & phys. props.	608

Supplementary Figure 1.1 | Chemical and physical variables included in the dataset. Correspondence between variables and symbols and number (N) of samples in the dataset containing that information. Created with BioRender.com.

Let us start by defining the chemical variables and explaining the different values they can take. When appropriate a numerical labelling equivalence for each value is given in square brackets, this is used in some of the statistical plots in later sections.

-The parameter "Chemical family" is categorical and takes one of the following 9 values for each sample: {$LnPc_2$ [1]; polyoxometalate [2]; Schiff base [3]; metallocene [4]; diketonate [5]; radical [6]; TM near Ln [7]; mixed ligands [8]; other families [9]}. Details on this classification are given in Supplementary Section 2.

-The parameter "Ln ion" is categorical and takes one of the following 10 values for each sample: {Pr^{3+} [1]; Nd^{3+} [2]; Sm^{3+} [3]; Gd^{3+} [4]; Tb^{3+} [5]; Dy^{3+} [6]; Ho^{3+} [7]; Er^{3+} [8]; Tm^{3+} [9]; Yb^{3+} [10]}.

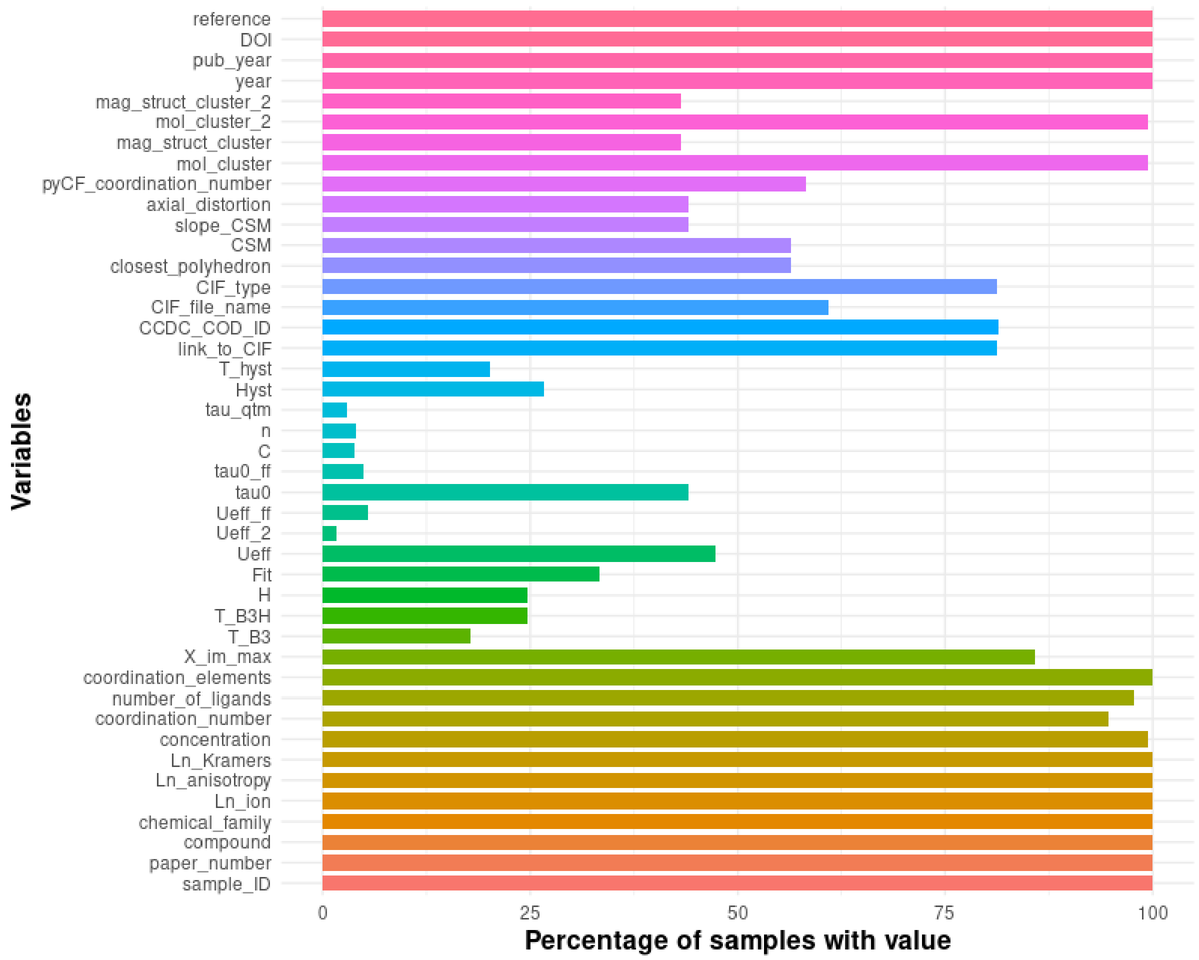

Supplementary Figure 1.2 | Percentage of samples with data included in the dataset. Percentage of samples containing valid values for each variable.

-The parameter "Ln anisotropy" is categorical and takes one of the following 3 values for each sample: {prolate [0]; oblate [1]; isotropic [2]}. This is determined directly by the Ln ion and presents a 1-to-1 correspondence with the sign of the Stevens α parameter. In the simplified zero field splitting Hamiltonian, this means that for an oblate ion (such as Dy^{3+} or Tb^{3+}) an axial coordination favours a high m_J ground state, whereas for a prolate ion (notably, Er^{3+}) it is the equatorial coordination the one that favours a high M_J ground state.

-The parameter "Ln Kramers" is categorical and takes one of the following 2 values for each sample: {non Kramers [0]; Kramers [1]}. Like the anisotropy, this is determined directly by the Ln ion.

-The parameter "concentration" takes the percentage value, e.g. concentration=1 is read as 1% concentration of the magnetic Ln ion in a diamagnetic matrix where 99% of the molecules are *e.g.* the Y^{3+} analog.

-The parameter "Coordination Number" (or CN) is an integer number between 2 and 9. Note that, for $LnPc_2$ we assigned CN = 8, as corresponding to the 8 N donor atoms; and for metallocenes we assigned CN = 2. From the point of view of steric hindrance in coordination chemistry, a Cp ring can be considered as equivalent to 3 coordination sites. However, from the point of view of the Crystal Field it creates and its coupling to "soft" vibrations, it is well-know that the electronic density responsible for the coordination is the π-electron cloud. Moreover, this π-electron cloud has a very reduced freedom to distort, due to the rigid nature of the aromatic ring. This is why we chose to treat the whole orbital (which in fact is delocalized over the rigid ligand) as a single coordination site for the purposes of the present study.

-The parameter "Number of ligands" is an integer number between 2 and 9, corresponding to the total number of ligands contributing donor atoms. The absolute number of ligands is registered, not the number of chemically different ligands: N identical ligands count as N.

-The parameter "Coordination Elements" is categorical and takes one of the following 5 values for each sample: {Oxygen [1]; Nitrogen [2]; Oxygen+Nitrogen [3]; Carbon [4]; Others [5]}. Any combination of oxygens and nitrogens is counted as "Oxygen+Nitrogen", and complexes with coordination elements different from O, N or C in the coordination sphere of Ln ions are in the category of "Others".

-The parameters "Closest polyhedron", "CSM" and "Axial distortion" are extracted from calculations as defined in Supplementary Sections 7, 8. "Closest polyhedron" is categorical, while "CSM" and "Axial distortion" are continuous numbers.

-The parameters of the type "Molecular cluster" are categorical and are extracted from statistical data processing as defined in Supplementary Section 4.2.

Let us continue by defining the physical variables.
-The parameter labelled as χ''_{max} (in plots), or $\chi_{im,max}$ (in data table), takes one of these possible values:
·[0]: Freq-independent χ'' (neither T_{B3} > 2 K reported, nor frequency-dependence in χ'' *vs* T),
·[1]: Freq-dependent χ'' (no T_{B3} > 2 K, but frequency-dependence in χ'' *vs* T measured),
·[2]: T_{B3} > 2 K, and
·[3]: Not Measured (no available data to assign the sample into one of the previous three categories).

-T_{B3} (T_{B3H}) is the temperature at which one finds the maximum value of χ'' *vs* T at 10^3 Hz, in absence (in presence) of an external magnetic field; H is the magnetic field, if present. It can be understood as the maximum temperature for which the system maintains short-term (millisecond) magnetic memory. For articles that provide χ'' *vs* T with a curve for each different frequency, we simply chose the curve corresponding to the frequency 10^3 Hz (or the closest one) and registered the temperature for the maximum χ'', or the absence of a maximum. However, if the articles represent χ'' *vs* frequency as isothermal curves for each different temperature, the same information is accessible indirectly by reading the points in the graph vertically at the abscissa value corresponding to the frequency 10^3 Hz and checking

in consecutive temperature curves whether χ" values present a non-monotonic evolution with respect to temperature, and therefore a maximum.

-"Fit" registers whether the parameters to determine U_{eff} and τ_0 were obtained from χ"(T) maxima at different frequencies or from an Argand plot.

-U_{eff}, $U_{eff,2}$, $U_{eff,ff}$ are the effective energy barriers and τ_0, $\tau_{0,ff}$ are the attempt times, which means the pre-exponential factors. The values of the effective energy barrier U_{eff} and of the attempt time τ_0 are recorded if they are determined from a fit considering a single Orbach process. In the cases where a second Orbach process is considered, we register (besides U_{eff}, τ_0 for the first process) its effective energy barrier $U_{eff,2}$. If a more complete model for relaxation is employed including an Orbach process as well as the Raman process, Quantum Tunnelling of the Magnetization and/or a direct process, we consider this a "full fit"(in short: ff), and record the value of the effective energy barrier $U_{eff,ff}$ and the attempt time or pre-exponential factor $\tau_{0,ff}$.

-The parameter labelled as "*Hyst*" takes one of the four values as follows:

·[0]: No hysteresis above 2 K reported,
·[1]: Pinched/butterfly Hysteresis (magnetic hysteresis above 2 K reported, but no magnetic coercivity field or remnant magnetisation can be determined; see Supplementary Fig. 1.3),
·[2]: Full Hysteresis (magnetic hysteresis above 2 K reported, and additionally either magnetic coercive field or remnant magnetisation can be determined; see Supplementary Fig. 1.3),
·[3]: Not Measured (no available data to assign the sample into one of the previous three categories).

To standardise data as far as possible, instead of taking the hysteresis temperatures as reported in the main text by the different researchers (that often employ different criteria) we examined all figures available ourselves and employed a uniform criterion to extract the data. As a consequence, in many cases our data do not coincide with the author's explicit claims.

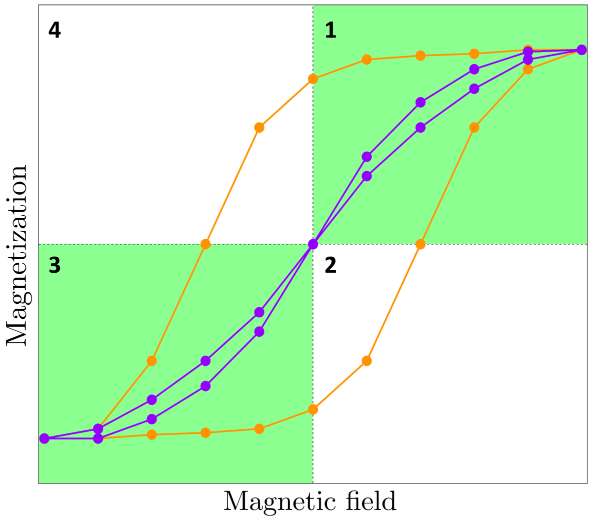

Supplementary Figure 1.3 | Full vs pinched (butterfly) hysteresis. Full hysteresis curves (orange) present at least one point either in quadrants 2,4 (*i.e.* different signs for Magnetic field and Magnetization) or in the x and/or y axes. Pinched (butterfly) hysteresis curves (violet) present points only in quadrants 1,3 (*i.e.* same signs for H and M) and, sometimes, also at the origin of coordinates.

-The related parameter "T_{hyst}" takes the highest temperature value at which hysteresis is reported. In contrast with T_{B3}, this quantifies the temperature up to which the system maintains long-term magnetic memory.

-The parameters of the type "Magneto Structural cluster" are categorical and are extracted from statistical data processing as defined in Supplementary Section 6.

One of the main problems for the data extraction during the construction of the dataset was that different criteria are chosen by different groups to characterise the hysteresis. For example, the hysteresis is measured only at 2 K in many studies, resulting in an overrepresentation of T_{hyst} = 2 in the dataset; while in many other cases the hysteresis is measured up to the highest possible temperature. Therefore, the very same compound could then present different T_{hyst} depending on an arbitrary choice by the researchers, decreasing the quality of the dataset. Another difficulty is that the information of the applied magnetic field sweep rate which directly affects T_{hyst} is missing in some articles. Note however that the works where data from more than a single sweep rate are reported demonstrate that the order of magnitude of T_{hyst} is very robust unless ultra-fast magnetic field pulses are employed. These extreme cases are discarded from our dataset (we only include sweep speeds (v) below 0.3 T/s), meaning that even when mixing data from different sweep rates within this range, the data points will consistently be in the right region of the graphs (e.g. cases with T_{hyst} = 3K will not be mistaken for T_{hyst} = 13K or T_{hyst} = 30K, nor vice versa). In addition, in some articles, not only a full hysteresis, presenting coercive field and magnetic remanence, is measured up to a certain temperature but also a pinched (butterfly) hysteresis is measured up to a higher temperature. This introduces some noise in the dataset, but is less problematic since both cases are going to be registered as a SIM with good properties.

Finally, it was not practical to include in the dataset other descriptive parameters such as τ_{switch} ,[1] the temperature at which the relaxation time is 100 s or the temperature at which there is a maximum in the zero-field cooled susceptibility because, while they will hopefully be the standard in the field, the number of publications reporting these parameters within our studied period (2003-2019) is too reduced to extract trustworthy statistical information from them.

1.1 The question of publication bias

Note that published results are generally biassed towards positive results, and this is generally considered as a problem in meta-studies. Publication bias may invalidate the conclusions unless they are robust to possible non-random selection mechanisms.[2] In the present case there are two important questions about this.

In the first place: how did we address the bias in available data towards positive results, and how abundant negative data are within our dataset? We addressed this in the design phase of our study by (a) performing an automated search for articles based on certain keywords (related to the topic, not necessarily with the result), (b) recording all negative data, which we found to be very abundant, especially in lower-impact journals and (c) distinguishing between different categories of negative results. For this it was key to record not just hysteresis data, where an absence of hysteresis is rarely acknowledged explicitly, but also the indirect information provided by ac susceptometry. By following this strategy, we found that the bibliography in this field is in practice very rich in negative results, whether these take the form of absolutely no ac signal, or of just a frequency dependence in ac susceptometry but no out-of-phase peak above 2 K. Even ac peaks at low temperature can be understood as negative results, since typically the compounds with hysteresis also behave well in ac (see Supplementary Figure 21).

Note that it is frequent that a series of compounds is studied in the same publication, changing either the Ln metal or doing systematic modifications in the ligands, and among them only some present good SMM properties, and in the vast majority of cases this is explicitly acknowledged in the text, so it is possible to extract negative data. There are also many cases of studies focused on other properties (e.g. optical), where the magnetic behaviour is recorded but does not determine publication. As we will see, from over 1400 samples, about 600 present no frequency-dependent out-of-phase magnetic susceptibility χ'', compared with 200 with no data, and about 300 each for a maximum in χ'' above 2K or no maximum but a frequency-dependent χ''. In that sense, the problematic data are related to hysteresis, where the vast majority of the samples (>1000) contain no information. While we did not rely on this for our analysis, it would be reasonable to assume that in most cases a lack of information on hysteresis means that the sample presents no hysteresis.

In the second place: what are the consequences? In the present study we were careful not to ask absolute questions which would be affected by publication bias, such as how often, out of a novel 100 complexes, Schiff base ligands are expected to produce certain results in terms of magnetic behaviour, and we do not extract conclusions from how many Dy SIMs have been reported, in absolute numbers, compared with Er SIMs. Instead, we always compare relative frequencies. One can safely assume that the effect of publication bias will affect equally different lanthanides, different chemical families, etc, meaning that relative comparisons should be safe from publication bias. From the dataset we want to answer relative questions such as:
-are $LnPc_2$, or metallocenes, distinctly promising as SIMs, compared with any other chemical families?
-the same for several other chemical families: are {Schiff bases, polyoxometalates, diketonates, radicals, TM near Ln} promising as SIMs, in relative terms? (see below for the definition of the families)
-are complexes of oblate ions (Dy,Tb…) better SIMs than prolate ions (Er…) in terms of ac susceptibility, higher U_{eff}, magnetic hysteresis?

-is U_{eff} as good a predictor for T_{hyst} as often assumed? Is it correlated with τ_0 and/or with Raman?
-are there any coordination polyhedra with high relative frequency of good magnetic behaviour?

Supplementary Section 2. Classification in chemical families

Lanthanides are a group of *f*-block elements with atomic numbers ranging from 57 (lanthanum) to 71 (lutetium). Most of the Ln elements exhibit the oxidation state of +3. Our dataset only includes the trivalent Ln^{3+} (Pr, Nd, Sm, Gd, Tb, Dy, Ho, Er, Tm and Yb) ions containing complexes. Ln ions possess large coordination numbers (CNs) due to their large ionic radii. The geometrical arrangement around these trivalent ions basically depends on the steric properties of the coordinated ligands; thus a suitable design of the ligand molecules leads to an easy tuning of the CNs. In particular, CNs between 2 and 12 are documented for Ln ions. Note that in this work we consider one rigid aromatic ring as equivalent to a contribution to CN/ring = 1 when it is of the cyclopentadienyl/cyclooctatetraenyl kind, whereas we consider a contribution of CN/ring = 4 when it is of the phthalocyaninato kind. In the low CN cases, the coordination ligands are usually bulky ligands, e.g. bis(trimethylsilyl) amine gives CN = 3; whereas cyclopentadienyl ligands need to be smartly substituted to achieve the same steric impediment. In contrast, in the case of complexes with high CN, the ligands are usually small bidentate ligands, such as nitrate and/or macrocyclic ligands. In the present work, we found that the most frequent are CN = 8 and CN = 9. This coincides with what is known for Ln ions, namely, Ln ions tend to spontaneously favour these CNs, typically with distorted square antiprismatic coordination (CN = 8) or distorted tricapped trigonal prism coordination (CN = 9).

Ln-based SIMs are interesting because the 4*f* electrons are less exposed to ligand field effects and exhibit larger spin-orbital coupling if compared with the *d*-shell. The first Ln-based mononuclear single molecule magnets (SMMs) were generated by Ishikawa and co-workers in 2003 using two macrocyclic ligands to sandwich the Ln^{3+} ion in a double-decker fashion.[3] They can also be prepared by using a range of acyclic ligands, such as polyoxometalates (POMs),[4–6] Schiff bases,[7,8] radicals,[9–17] and ketones.[18–20] Between 2003 and 2019, several hundreds of articles referring to Ln-based SIMs have been published. Among them, the vast majority focused on the chemical approaches in designing lanthanide-based SIMs with superior properties. A fundamental key parameter of the magnetic properties of SIMs is the molecular symmetry which can be controlled by: (a) the ligand design and modification, (b) the substitution of the coordination elements as a means to alter electrostatic potential and/or Ln to coordination atom bond lengths, and (c) the peripheral ligand functionalization/substitution. Here, we classify the collected complexes into 9 categories according to the type of coordination ligands or the chemical strategy used for the design of the magnetic complex. These 9 categories (Chemical Family) are listed below and will be briefly described in this section.
1) $LnPc_2$ family
2) POM family
3) Schiff Base family

4) Metallocene family
5) Diketonate family
6) Radical family
7) TM near Ln family
8) Mixed ligands family
9) Other families

Note that endohedral metallofullerenes, nowadays a very promising SIM family, have not been classified as a separate family in the present study merely because at the point where we started designing the data collection, metallofullerenes were still quite scarce and not yet established as a SIM family. They could be included in a future update of the dataset. Radical-bridge dimers are now recognized to be strong candidates but were not included in the study at all because we considered that they introduce extra degrees of freedom that would only apply to a minority of cases. They would require their own study, which at the present time cannot be statistics-based.

2.1. $LnPc_2$ family

The first category is constituted by "double-decker complexes" related to the classical $LnPc_2$ family, namely, the Ln ion in the complex is octa-coordinated by nitrogen atoms from two Pc (or their related functionalized complexes, or porphyrin-like, or even tetraaza[14]annulenes) ligands. As we will see below, this criterion has priority over the presence of a spin S = 1/2 (radical ligand, in this case corresponding to oxidised or reduced Pc ligands) and also over the presence of diamagnetic transition metal ions in the vicinity (in this case often corresponding to multiple deckers which coordinate Cd^{3+}). In both cases, these complexes are classified as the $LnPc_2$ family. Complexes composed of phthalocyanine ligands or porphyrins with nitrogen-based donating atoms have shown very important roles in Ln-based SIMs. There are several reasons for choosing phthalocyanines and porphyrins for SIM design: a) these tetrapyrrole macrocyclic ligands containing four isoindole or pyrrole nitrogen atoms have the ability to strongly coordinate to Ln ions; b) special features such as intramolecular π-π stacking interaction and the intrinsic nature of their macrocyclic rotation; and c) their structural characteristics of those sandwich-type complexes since the ligand field constructed by this type of ligands with a C_4-symmetric axis (pseudo-D_{4d} symmetry) is very important for the zero-field splitting of the ground state into the magnetic sublevels. The combination of the large magnetic anisotropy with strong spin-orbital interactions leads to the SIMs behaviours.

The first examples of Ln-based SIMs reported are from the $LnPc_2$ family, which was proposed in 2003 by Ishikawa and co-workers.[3] They successfully demonstrated that slow magnetic relaxation could occur in mononuclear lanthanide complexes, such as those in which a Ln ion is sandwiched between two Pc ligands, formulated as $(Bu_4N)[LnPc_2]$ (Ln^{3+} = Tb^{3+} or Dy^{3+}, Bu_4N = tetrabutylammonium) (Supplementary Fig. 2a). Later on, a massive synthetic effort has led to an ever-increasing number of compounds from the $LnPc_2$ family, which includes the introduction of a wide range of substituents at the periphery of the Pc macrocycles without significantly interfering with the metal binding properties of the ligands.[21–25] Some structure representations of examples from this family studied in this work

are shown below (Supplementary Fig. 2), as the sandwich complex $[Bu_4N][DyPc(OTBPP)]$, (Supplementary Fig. 2b) in which one of the nitrogen atoms of one porphyrin pyrrole is replaced by an oxygen atom. Compared with the typical $LnPc_2$ complex, the atom replacement significantly enhances the effective energy barrier of the SIMs.[25] Another example is the use of tetraazaporphyrins (or porphyrazines) in place of the bulkier Pc ligands, giving rise to a series of neutral double-decker complexes that show analogous magnetic features as their Pc counterparts (Supplementary Fig. 2c).[26]

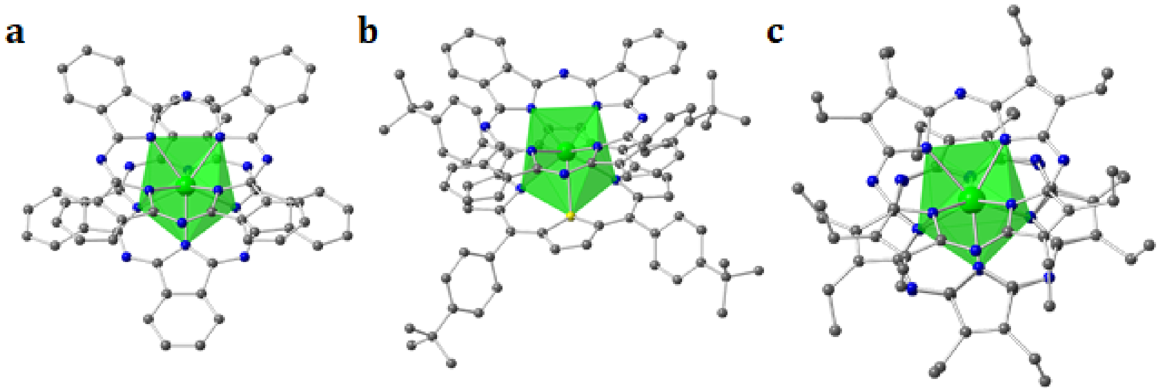

Supplementary Figure 2 | Combined polyhedral and ball-and-stick models of the coordination spheres around Ln ions of some cases from the $LnPc_2$ family. a, $[Pc_2Ln]^-$ from reference [[3]]. **b**, The sandwich-type mixed phthalocyaninato with core-modified porphyrinato double-decker complexes $[DyPc(OTBPP)]^+$ or $[Dy(Pc)(STBPP)]^+$ from reference [[25]]. **c**, $[Ln(OETAP)_2]^+$, where OETAP is octa(ethyl)tetraazaporphyrin.[26] (Color code: grey sphere, C; green sphere and polyhedron, Ln; blue sphere, N.)

2.2. POM family

The second representative family consists of polyoxometalates (POMs). This family contains all compounds where Ln^{3+} ions coordinate with POM ligands, including the cases where the coordination sphere is completed with other ligands. POMs are molecular metal-oxo clusters with early transition metals (W, Mo, Nb, Ta or V) in their highest oxidation states. The ability of these inorganic species to incorporate almost any kind of metal or non-metal addenda heteroatoms, together with their enormous molecular and electronic structural diversity, makes them of relevance in the molecular magnetism field. One relevant feature of POM ligands is that their diamagnetic structures can encapsulate Ln ions with coordination geometries similar to those of bis(phthalocyaninato)lanthanide complexes from $LnPc_2$ family.[3] More recently, POMs were used as extremely versatile inorganic building blocks for the construction of SMMs based either on 3*d* or 4*f* metal ions.[27] Some representative cases of complexes included in this study are shown below in Supplementary Fig. 3. The first example from the POM family exhibiting SIM behaviour is $[ErW_{10}O_{36}]^{9-}$ (Supplementary Fig. 3a).[4] Later on, two families of POM-based SIMs with formula $[Ln(W_5O_{18})_2]^{9-}$ and $[Ln(\beta_2\text{-}SiW_{11}O_{39})_2]^{13-}$ (Supplementary Fig. 3b) are reported in 2009, both of which show slow relaxation of the magnetisation, typical of the SIM-like behaviour.[5] Another well-known

series of complexes is $[LnP_5W_{30}O_{110}]^{12-}$ (Supplementary Fig. 3c), in which its unusual C_5 axial symmetry allows the study of new SIMs having 5-fold symmetry. The Dy^{3+} and Ho^{3+} derivatives exhibit SIM behaviour.[6]

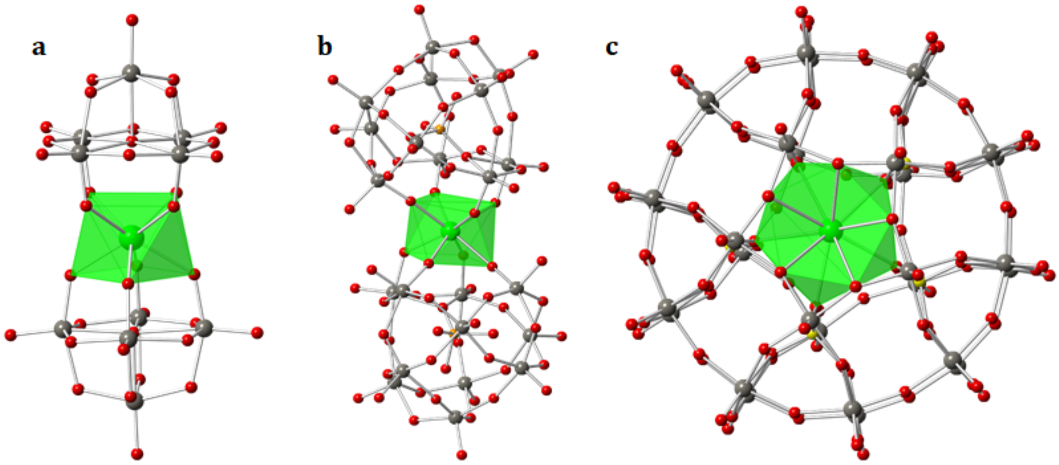

Supplementary Figure 3 | Combined polyhedral and ball-and-stick models of the coordination spheres around Ln ions of three representative cases from the POM family. **a**, $[ErW_{10}O_{36}]^{9-}$ from reference [4]. **b**, $[Ln(\beta_2\text{-}SiW_{11}O_{39})_2]^{13-}$ from reference [5]. **c**, $[LnP_5W_{30}O_{110}]^{12-}$ from reference [6]. (Color code: grey sphere, W; green sphere and polyhedron, Ln; red sphere, O; yellow sphere, P; orange sphere, Si.)

2.3. Schiff base family

The third family is based on Schiff base ligands. This includes all samples where the Ln^{3+} ion coordinates to only Schiff base ligands; in addition, we included the cases where the strategy pursued by the authors (as stated in the title) relies on Schiff base ligand, even if other small ligands are used to complete the coordination sphere. Schiff base ligands are polydentate macrocyclic or macro-acyclic ligands, which typically contain both nitrogen and oxygen donors. However, the donor atom can be varied between sulfur, phosphorus, nitrogen, and oxygen. Due to their facile synthesis, Schiff base ligands are considered to be "privileged ligands", which can easily make a coordination bond with many different metal ions and stabilize them in various oxidation states. In addition, when two equivalents of salicylaldehyde are combined with a diamine, a particular chelating Schiff base is produced, which is called salen ligands. Salen ligands present four coordinating sites (tetradentate) and two axial sites that are open to ancillary ligands, thus similar to porphyrins but with an easier preparation process.

Schiff bases derived from condensation reactions of aromatic aldehydes with primary amines have been the subject of extensive research because of their enormous versatility with respect to the formation of metal complexes with sophisticated discrete or expanded architectures and functional properties. The choice of initial reagents for the condensation determines the ligand coordination fashion and allows one to utilize both chelate and bridging functions of the obtained Schiff base. Schiff base complexes continue to intrigue chemists regarding their structure and reactivity. Their geometries are strongly influenced by the ligands and tend to be five- or six-coordinate. The first case listed here comprises two mono-deprotonated Schiff

base $[LH]^-$ ligands, showing SIM behaviour and with a U_{eff} of 44.4 K in presence of a dc field (Supplementary Fig. 4a).[28] Another case from this family is the Dy^{3+} complex with tridentate NNO ligands of N-[(imidazol-4-yl)methylidene]-DL-alanine (Supplementary Fig. 4b), which shows an out-of-phase signal with frequency-dependence in ac susceptibility under a dc bias field of 10^3 Oe, indicative of field induced SIM.[29] One other representative case from this family is the Salen-type mononuclear Ln^{3+} complex $[Ln(3\text{-}NO_2\text{-salen})_2]^-$ (Supplementary Fig. 4c), which shows slow magnetisation relaxation processes associated with SIM behaviour.[30]

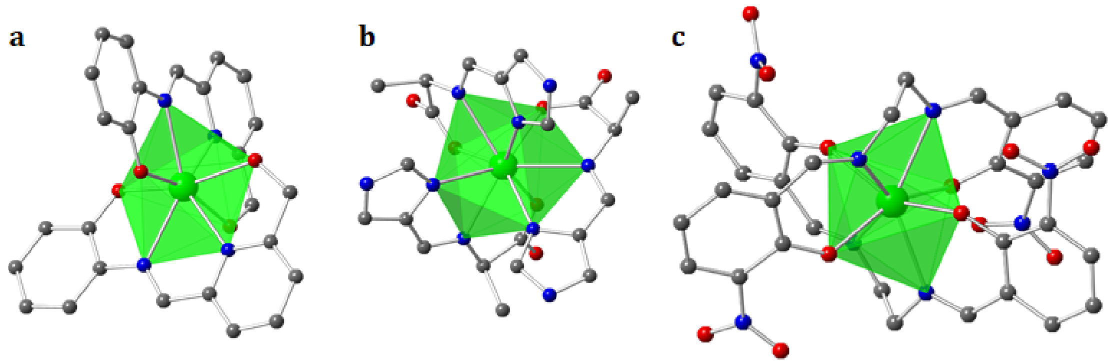

Supplementary Figure 4 | Combined polyhedral and ball-and-stick models of the coordination spheres around Ln ions of some cases from the Schiff base family. a, $[Ln(LH)_2]^-$ where H_2L = 2-((6-(hydroxymethyl)pyridin-2-yl)-methyleneamino)phenol.[28] **b**, '*fac*'-$[Dy^{III}(HL^{DL\text{-}ala})_3]$, where $H_2L^{DL\text{-}ala}$ is N-[(imidazol-4-yl)methylidene]-DL-alanine.[29] **c**, $[Ln(3\text{-}NO_2\text{-salen})_2]^-$, where Ln can be Dy, Er or Yb, and 3-NO_2-salen^{2-} = N,N'-bis(3-nitro-salicylaldehyde)ethylenediamine dianion.[30] (Color code: grey sphere, C; green sphere and polyhedron, Ln; red sphere, O; blue sphere, N.)

2.4. Metallocene family

The fourth family is based on the small aromatic ligands derived from conjugated hydrocarbon ligands, typically cyclopentadienyl or cyclooctatetraene anions. We only include in this classification the complexes where the coordination sphere is completed by this kind of ligands, in contrast with cases with an extra "equatorial" coordination site. Compared with heteroatomic donor atoms such as oxygen and nitrogen, which have limited orbital overlap with the shielded 4*f* orbitals, the aromatic ligands allow the perturbation of the crystal field of the lanthanide ions through the use of an electron π-cloud. Thus, it can further control over the anisotropic axis and induction of *f-f* interactions, making donor atoms as conjugated hydrocarbons.[31] Here we list some examples by employing delocalized ligands to design SIMs with prominent uniaxial anisotropy. An Er^{3+} ion sandwiched by two aromatic ligands, pentamethylcyclopentadienide anion ($C_5Me_5^-$, Cp*) and cyclooctatetraenide dianion ($C_8H_8^{2-}$, COT) (Supplementary Fig. 5a), displays a butterfly-shaped hysteresis loop at 1.8 K up to even 5 K.[32] Another example is a bis-monophospholyl Dy^{3+} SIM, $[Dy(Dtp)_2][Al\{OC(CF_3)_3\}_4]$ (Supplementary Fig. 5b), which shows an effective energy barrier to magnetisation reversal of 1760 K (1223 cm^{-1}) and magnetic hysteresis up to 48 K.[33] The use of planar cyclooctatetraenide (COT''^{2-}) ligands allows the access to the sandwich type complex

$[Dy(COT'')_2]Li(DME)_3$ (Supplementary Fig. 5c), which exhibits slow relaxation of the magnetisation indicating its SIM behaviour.[34]

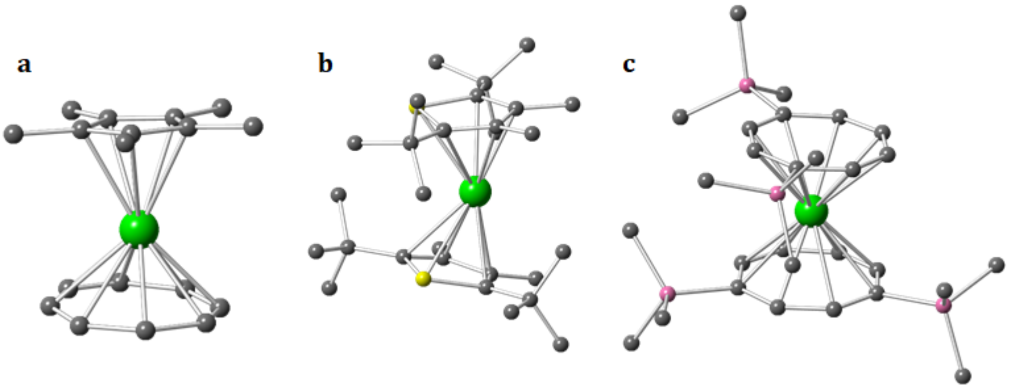

Supplementary Figure 5 | Ball-and-stick models of the coordination spheres around Ln^{3+} ions of some cases from the metallocene family. a, (Cp*)Er(COT), where Cp* = $C_5Me_5^-$ and COT = $C_8H_8^{2-}$, from reference [32]. **b**, $[Dy(Dtp)_2][Al\{OC(CF_3)_3\}_4]$, where Dtp = $\{P(C^tBuCMe)_2\}$.[33] **c**, $[DyCOT''_2]^-$, where COT''^{2-} = cyclooctatetraenide rings.[34] (Color code: grey sphere, C; green sphere, Ln; pink sphere, Si; yellow sphere, P.)

2.5. Diketonate family

The fifth family is the diketonate family of complexes, it includes those samples with Ln^{3+} ions coordinated with diketonate ligands and diketonate ligands mixed with other molecules which are not defined in the classification. The diketonate ligands are bidentate and bond through delocalized chelate rings formed through two oxygen atoms. *β*-diketone SIMs have received much attention in recent years, since *β*-diketone can provide a stable bidentate chelating mode to afford eight-coordinated mononuclear lanthanide complexes. There are two different polyhedron coordination geometries for the *β*-diketone complexes, square antiprism with D_{4d} symmetry and triangular dodecahedron with D_{2d} symmetry. After the SMM behaviour of a simple acetylacetonate complex has been reported on several β-diketone complexes, much effort is devoted to the synthesis and investigation of *β*-diketone SIMs. In addition to the coordination geometry, the stability of the SIMs upon heating is also an important topic. Lanthanide *β*-diketone complexes with fluorides as substituent groups, such as hexafluoroacetylacetone (hfac), can make the complexes stable upon heating. By using the *β*-diketonate ligand dibenzoylmethane (DBM) anion, mononuclear Dy complex $[Hex_4N][Dy(DBM)_4]$ (Supplementary Fig. 6a) was obtained, in which slow magnetic relaxation is observed.[18] A typical compound of *β*-diketone is formulated as (cation)[Ln(*β*-diketone)$_4$], in which the Ln^{3+} ion is surrounded by four *β*-diketone forming a LnO_8 environment. The complex shown in Supplementary Fig. 6b, using hfac ligand, exhibits field-induced slow magnetization relaxation.[19] Another case is the use of a sulfonyl amidophosphate (SAPh), acting as a *β*-diketone homologue for the complexation of Ln ion, which gives rise to complex LnL_3Phen (L = $C_6H_5SO_2NP(O)[N(CH_3)(C_6H_5)]_2$) with in-field SIM behaviours (Supplementary Fig. 6c).[35]

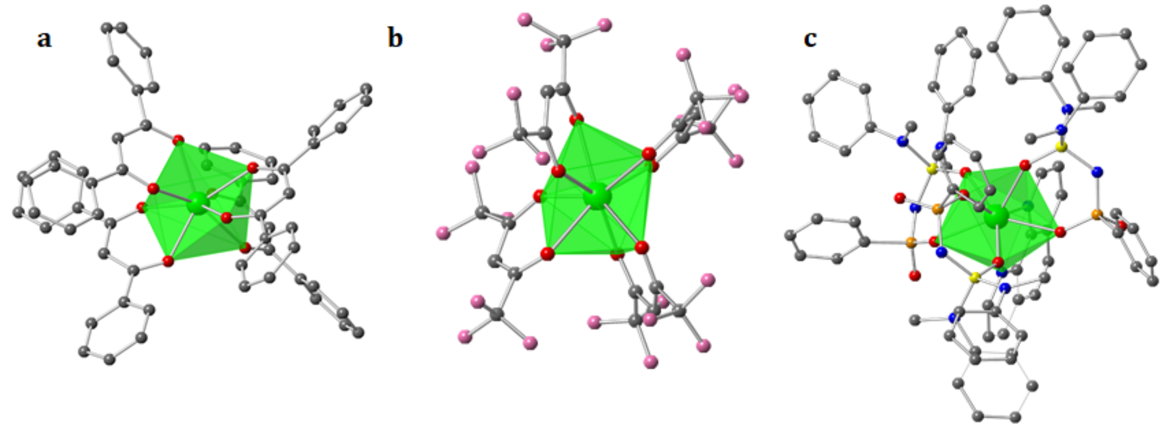

Supplementary Figure 6 | Combined polyhedral and ball-and-stick models of the coordination spheres around Ln ions of some cases from the diketonate family. a, $[Dy(DBM)_4]^-$, where DBM = dibenzoylmethane anion ligand.[18] **b**, {$Dy(hfac)_4$}, where hfac = hexafluoroacetylacetone.[19] **c**, [LnL_3(phen)], where Ln can be Dy or Er, and L is deprotonated bis(methyl(phenyl)amino)phosphoryl)-benzenesulfonamide, $C_6H_5SO_2NP(O)[N(CH_3)(C_6H_5)]_2$, and Phen = phenanthroline.[35] (Color code: grey sphere, C; green sphere and polyhedron, Ln; red sphere, O; yellow sphere, P; pink sphere, F; blue sphere, N; orange sphere, S.)

2.6. Radical family

The sixth family is composed of complexes in which Ln^{3+} ion is coordinated with radical-based ligand(s), such as nitronyl nitroxide and semiquinones. Radical ligands are one of the most efficient bridging ligands for the design of molecular magnetic materials.[36] The radical systems are relatively abundant in our dataset, being more numerous than any other family presented so far. The reason of choosing radical ligands is that they possess 2*p* diffuse spin orbitals that can potentially penetrate the core electron density of the lanthanide ions to reach deeply buried 4*f* orbitals, whose shielded magnetic orbitals are usually a drawback for their use in extended magnetically coupled structures.[37] The strong 2*p*-4*f* heterospin exchange coupling effectively shifts degenerated m_J sublevels to different energies and, furthermore, significantly reduces the probability of resonant quantum tunnelling and lengthens the relaxation time. We will show some examples from this family (Supplementary Fig. 7). The first case is the nitronyl nitroxide radical complex [$Ln(tfa)_3$(NIT-BzImH)], (Supplementary Fig. 7a) in which Ln^{3+} ion is 8-coordinated to one NIT-BzImH and three trifluoroacetylacetonate (tfa) ligands. It shows slow magnetic relaxation suggesting that they behave as SIMs.[12] The second case is a dinuclear Ln^{3+} compound with its formula as {Cp_2Co}{$[Dy(tmhd)_3]_2$(bptz)} in radical anion form (Supplementary Fig. 7b), in which the rare earth ions are isolated by an organic ligand bridged species. It exhibits out-of-phase ac susceptibility signals below 4 K.[17] Another relevant case is a cyclic dimer structure, in which each pyridine substituted radical links two different metal ions through the oxygen of a nitroxide group and the pyridine nitrogen (Supplementary Fig. 7c). It shows frequency-dependent ac magnetic susceptibility, indicating SIM behaviour.[11]

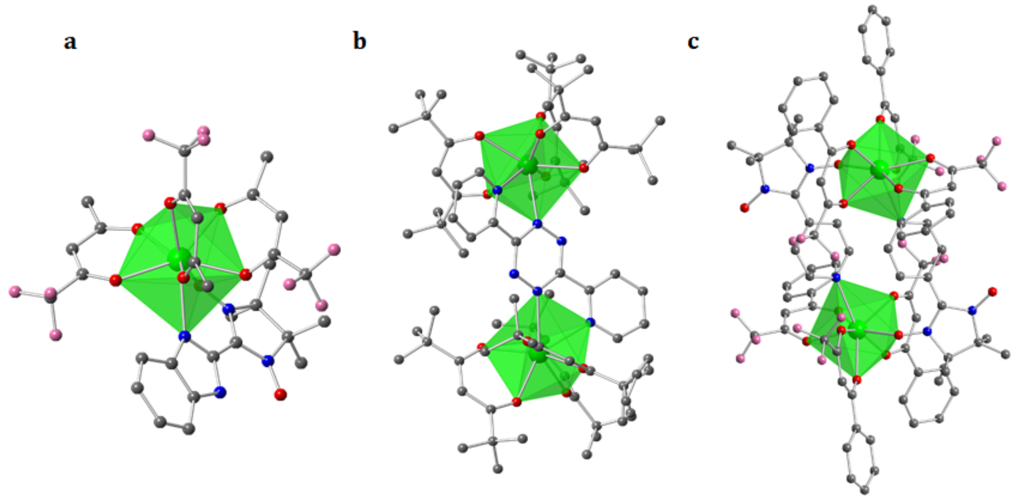

Supplementary Figure 7 | Combined polyhedral and ball-and-stick models of the coordination spheres around Ln ions of some cases from the radical family. a, [Ln(tfa)$_3$(NIT-BzImH)], where tfa = trifluoroacetylacetonate; NIT-BzImH = 2-(2′-benzimidazolyl)-4,4,5,5-tetramethylimidazolyl-1-oxyl-3-oxide.[12] **b**, {Cp$_2$Co}{[Dy(tmhd)$_3$]$_2$(bptz)}, where tmhd = 2,2,6,6-tetramethyl-3,5-heptane dionate and bptz = 3,6-bis(2-pyridyl)-1,2,4,5-tetrazine.[17] **c**, [Ln(Phtfac)$_3$(NITpPy)]$_2$, where HPhtfac = 4,4,4-trifluoro-1-phenylbutane-1,3-dione and NITpPy = 2-(4-pyridyl)-4,4,5,5-tetramethyl-4,5-dihydro-1H-imidazolyl-1-oxyl-3-oxide.[11] (Color code: grey sphere, C; green sphere and polyhedron, Ln; red sphere, O; pink sphere, F; blue sphere, N.)

2.7. TM near Ln family

This family of complexes is defined when a diamagnetic transition metal (TM) ion exists in the coordination sphere of Ln^{3+} ion. There are several Ln-based SIMs containing one or more 3*d* metal ions.[38–41] Most of this type of complexes contain Schiff base ligand or a diketone ligand.[38] For example, Yamashita *et al.* reported an Er-based SIM,[42] where the Er^{3+} ion is coordinated with a Schiff base ligand, which in turn is connected to the diamagnetic transition metal Zn^{2+} through oxygen. Macrocyclic ligands provide discrete metal binding pockets and, therefore, offer a more predictable cluster nuclearity and structure than acyclic analogues can.[38] For example, the [3+3] macrocycle provides three N_2O_2 pockets for 3*d* metal ions and one central O_6 pocket for a Ln ion, making mixed-metal M_3Ln tetrametallic macrocyclic complexes predictable.[42,43] Macrocycles usually provide enhanced stability, solubility and fine-tunability (vary the choice of M and Ln, whilst retaining the M_3Ln core) over acyclic analogues. It's documented that the U_{eff} of Ln-based SIMs can be enhanced by introducing diamagnetic metal ions in the coordination sphere. The diamagnetic ion may induce large electrostatic interaction between the Ln^{3+} ion and coordination atoms, giving rise to the destabilization of excited states and increasing the gap between the ground state and the first excited state.[44–46] There are many compounds that fall into the “diamagnetic TM near the Ln center” category. For instance, the pentagonal-bipyramid (quasi-D_{5h}) [Zn–Dy–Zn]

complex (Supplementary Fig. 8a) exhibits a large thermally activated barrier with long relaxation times.[46] Other cases are $\{[Zn(Me_2valpn)]_2Dy(H_2O)Cr(CN)_6\}_2$ (Supplementary Fig. 8b)[41] and $[Zn(\mu\text{-L})(\mu\text{-OAc})Er(NO_3)_2]$ (Supplementary Fig. 8c)[47] , both exhibit SIM behaviour.

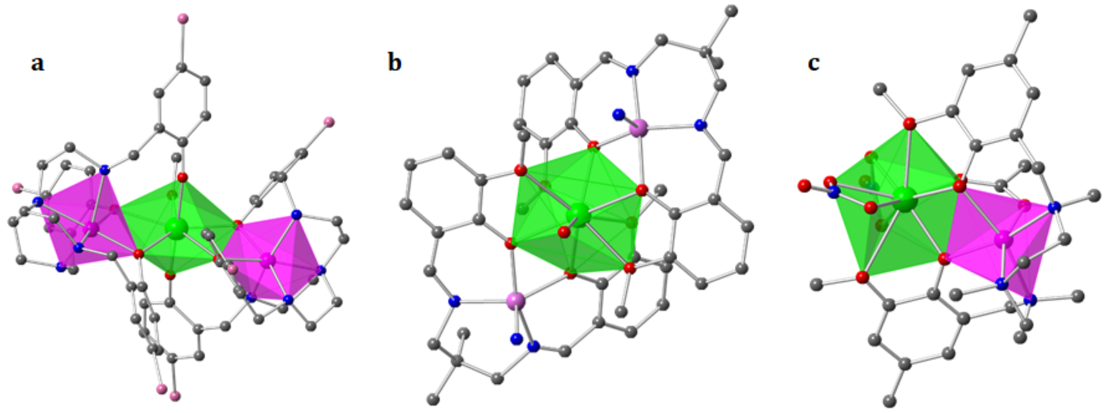

Supplementary Figure 8 | Combined polyhedral and ball-and-stick representations of the coordination spheres around Ln ions of examples from the TM near Ln family. a, $[Zn_2DyL_2(MeOH)]^-$, where L is 2,2',2''-(((nitrilotris(ethane-2,1-diyl))tris(azanediyl))tris(methylene))tris-(4-bromophenol).[46] **b**, $\{[Zn(Me_2valpn)]_2Dy(H_2O)Cr(CN)_6\}_2$, where Me_2valpn^{2-} is dianion of N,N'-2,2-dimethylpropylenebis(3-methoxysalicylideneimine).[41] **c**, $[Zn(\mu\text{-L})(\mu\text{-OAc})Er(NO_3)_2]$, where H_2L is N,N′,N″-trimethyl-N,N″-bis(2-hydroxy-3-methoxy-5-methylbenzyl)diethylenetriamine.[47] (Color code: grey sphere, C; green sphere and polyhedron, Ln; magenta sphere and polyhedron, Zn; red sphere: O; pink sphere, Br; blue sphere: N.)

2.8. Mixed ligands family

The eighth category is defined as mixed ligands. It contains all cases where the Ln^{3+} ion is coordinated with one kind of ligands defined above together with another ligand not defined, thus, mixed ligands. The design strategy of using mixed ligands for high performance SIMs is promising. There are many complexes from this category which possess SIM behaviour. One example is using N,N'-bis(2-hydroxybenzyl)-N,N'-bis(2-methylpyridyl)ethylenediamine and Cl (or Br) as ligands for synthesis of the seven-coordinate complex $[Dy(bbpen\text{-}CH_3)X]$, which produces high performance SIMs (Supplementary Fig. 9a).[48] Another representative case is the half-sandwich organometallic complex $[Cp^*Dy(DBM)_2(THF)]$ (Supplementary Fig. 9b) with a Janus structural motif, where the ligands are composed of THF, DBM^- and $[Cp^*]^-$. It displays slow magnetic relaxation in the absence of an applied magnetic field, indicating SIMs properties.[49] By combination of *β*-diketonate with 6-pyridin-2-yl-[1,3,5]triazine-2,4-diamine ligands, a series of SIMs were obtained and investigated (Supplementary Fig. 9c).[50]

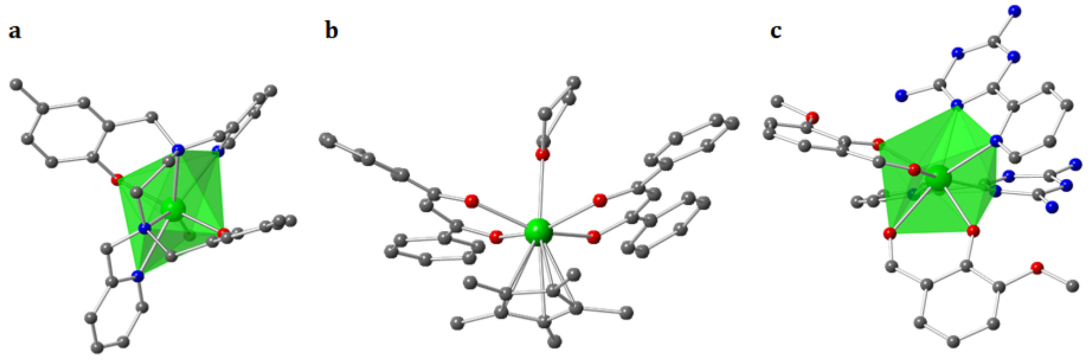

Supplementary Figure 9 | Combined polyhedral and ball-and-stick models of the coordination spheres around Ln ions of some cases from the mixed ligands family. a, [Dy(bbpen-CH_3)X], where X = Cl or Br and H_2bbpen = N,N'-bis(2-hydroxybenzyl)-N,N'-bis(2-methylpyridyl)ethylenediamine.[48] **b**, [Cp*Dy(DBM)$_2$(THF)], where Cp*=$C_5Me_5^-$ and DBM$^-$=dibenzoylmethanoate anion.[49] **c**, [DyLz$_2$(o-vanilin)$_2$]$^+$, where Lz = 6-pyridin-2-yl-[1,3,5]triazine-2,4-diamine and X = Br^-, NO_3^-, $CF_3SO_3^-$, from reference [[50]]. (Color code: grey sphere, C; green sphere and polyhedron, Ln; red sphere, O; blue sphere, N.)

2.9. Other families

The last category is named as "other families". It includes all complexes which fall into the criterion of complex selection but the coordination ligands of Ln ions are not in the ligand families previously defined. Large numbers of complexes included in this work are from this category. For instance, the octahedral dysprosium aluminate complex [Dy(AlMe$_4$)$_3$] shows fast relaxation of the magnetisation via quantum tunnelling (Supplementary Fig. 10a).[51] Also, the alkoxide cage complexes [DyY$_3$K$_2$O(O^{t}Bu)$_{12}$] and [DyY$_4$O(O^{i}Pr)$_{13}$] (Supplementary Fig. 10b) incorporate a small amount of $DyCl_3$ in the synthesis of [Dy$_4$K$_2$O(O^{t}Bu)$_{12}$] and [Dy$_5$O(O^{i}Pr)$_{13}$] to produce {DyY$_3$K$_2$} in a {Y$_4$K$_2$} matrix, or {DyY$_4$} in {Y$_5$}. These complexes show a single dominant relaxation process with very high U_{eff} values.[52] Another relevant cases are the five-coordinate complexes Ln(NHPh^{i}Pr$_2$)$_3$(THF)$_2$, (Ln = Dy and Er), with trigonal bipyramidal geometry, both of which exhibit slow magnetic relaxation under a zero/non-zero dc applied magnetic field (Supplementary Fig. 10c).[53]

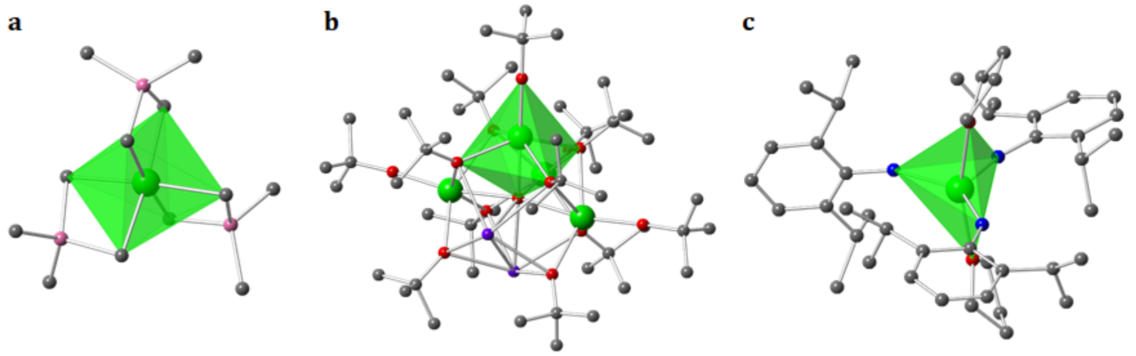

Supplementary Figure 10 | Combined polyhedral and ball-and-stick models of the coordination spheres around Ln ions of three examples from the other families. a, $[Dy(AlMe_4)_3]$ from reference [51]. **b**, $[DyY_3K_2O(O^tBu)_{12}]$ from reference [52]. **c**, $Ln(NHPh^iPr_2)_3(THF)_2$, in which Ln^{3+} can be Dy^{3+} or Er^{3+}, from reference [53]. (Color code: grey sphere, C; green sphere and polyhedron, Ln or Y^{3+}; red sphere, O; blue sphere, N; pink sphere, Al.)

Supplementary Section 3. A graphical, interactive, browsable App

To facilitate a broader use by the chemical community of the data collected in the present study, we developed the tool SIMDAVIS (Single Ion Magnet DAta VISualization): a graphical, interactive, browsable online database of over 1400 samples. Employing SIMDAVIS, any user can study the data in different and complementary ways. The four modes of operation, accessible in different tabs within the program, are “ScatterPlots”, “BoxPlots”, “BarCharts” and “Data” table. There is also an information subtab denoted as “Variables” within the “About SIMDAVIS” tab in which the definition of each variable can be found.

The basic use of the “ScatterPlots” tab is the representation of quantitative data against each other, *e.g.* the maximum hysteresis temperature (T_{hyst}) *vs* the effective energy barrier (U_{eff}). This allows a visual estimate on the relation between different experimental and theoretical descriptors of the magnetic behaviour. Other relevant numerical variables in the dataset include T_{B3}, T_{B3H}, the alternate estimate for the effective energy barrier ($U_{eff,ff}$), or the pre-exponential factors τ_0, $\tau_{0,ff}$, for either the simplistic equation or the full fit (see details about the variables in Supplementary Section 1). Furthermore, the “ScatterPlots” tab allows to distinguish the data points plotted according to a number of qualitative (categorical) variables, which can be of chemical nature, such as the chemical family, or which lanthanide ion was employed. Also, you may distinguish the points by some categorical variables of physical nature, such as presence or absence of magnetic memory above 2 K, in form of hysteresis or maximum in the χ'' (categorical variable χ''_{max} in our dataset). It also allows the user to select or deselect the represented data depending on these qualitative variables, to help distinguish quantitative correlations that might be different for different classes of compounds. Finally, there is also an option to fit linear regressions between the two represented quantitative variables for each of the categorical classes. These variations can combine to hundreds of thousands of distinct meaningful plots.

The "BoxPlot" tab allows a different type of representation. One can plot the values of any of the quantitative variables *vs* any of the qualitative variables, for a total of 108 possible variable pairs producing distinct representations. The distribution of a single quantitative variable (*e.g.* U_{eff}) is represented, showing the data points, the median, the low (first or Q1) quartile, upper (third or Q3) quartile, and whiskers. The upper whisker extends from the hinge to the largest value no further than 1.5xIQR from the hinge (where IQR is the interquartile range, or distance between the first and third quartiles). The lower whisker extends from the hinge to the smallest value at most 1.5xIQR of the hinge. This representation is done in parallel for different values of a qualitative variable, *e.g.* "Coordination Elements". An advantage of these boxplots *vs* the more sophisticated scatterplots is a larger amount of data to be represented at any given time. Note that there is virtually no paper that contains simultaneously all the kinds of information recorded in the dataset. For example, only a minority of the papers have historically performed a full fit considering Orbach, Raman, quantum tunneling and/or direct mechanism of relaxation. This means that the scatter plots, by being restricted to samples where two particular quantitative data kinds are well defined, effectively work with less data, so while they enable us to extract more nuanced dependencies, inevitably some information is lost.

In the "BarCharts" tab, the different qualitative data types can be represented vs each other, for a total of 144 variable pairs producing distinct representations. Since qualitative information is available for almost all samples, bar charts contain almost all data points and allow for a quick frequency check of frequencies of different values in the dataset. Again, they provide a complementary mode of analysis of correlations. In our case, rather than the standard bar chart, SIMDAVIS employs stacked bar graphs meaning we can analyze the covariation of two variables, *e.g.* Tb^{3+} is more common in the SIM literature than Er^{3+}, but whereas this is especially true for the chemical families of "$LnPc_2$" and "radicals", the reverse trend is found for the metallocene family.

The "Data" tab contains a mini-menu with two options: it allows the user to download the raw dataset, and it allows the user to browse the data set. The browsing is interactive in different, complementary ways. First, it allows the user to select the columns to show, *e.g.* by default each entry just shows 7 columns of data, namely the sample ID, formula of the compound, its chemical family, the Ln ion, the coordination elements, U_{eff}, and the DOI of the article where the information was obtained, while the other 24 columns are hidden. Second, it allows the user to arrange the information by ascending or descending order of the chosen variable. Finally, it includes a search tool that filters for text strings in real time.

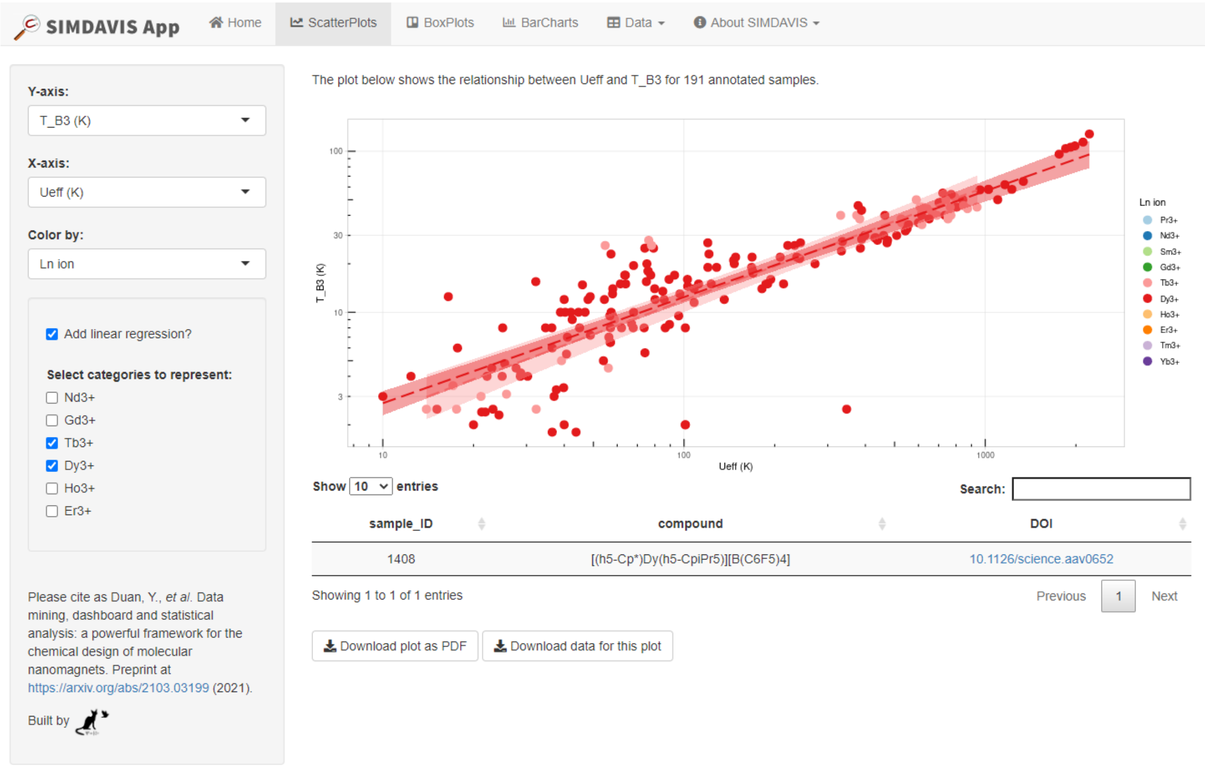

Figure 11 | Screenshot of the SIMDAVIS dashboard. It shows an example plot employing the "ScatterPlots" tab, where the users can represent 9 quantitative physical properties versus another in logarithmic scales, as well as a chemical qualitative variable from a dropdown menu, which contains 12 qualitative categorization possibilities; each data point is identified by a colour corresponding to its category. This permits the interactive exploration of hundreds of potential magnetostructural correlations between chemical variables, measured experimental values and parameters fitted from physical measurements. In the example, T_{B3} *vs* U_{eff} presented. Checkboxes were used to add a linear regression for each category and to hide all metal ions except for Tb^{3+} and Dy^{3+}. This visual estimate on the relation between descriptors of the magnetic behaviour may uncover trends for specific qualitative variables. At any time, the chosen plots can be downloaded as vectorial PDF files. In the example, the data point with the highest T_{B3} was clicked to display its sample ID, compound name and DOI linking to the article, facilitating further analysis.

3.1. Gallery of graphs: chemical variables to optimise the physical properties

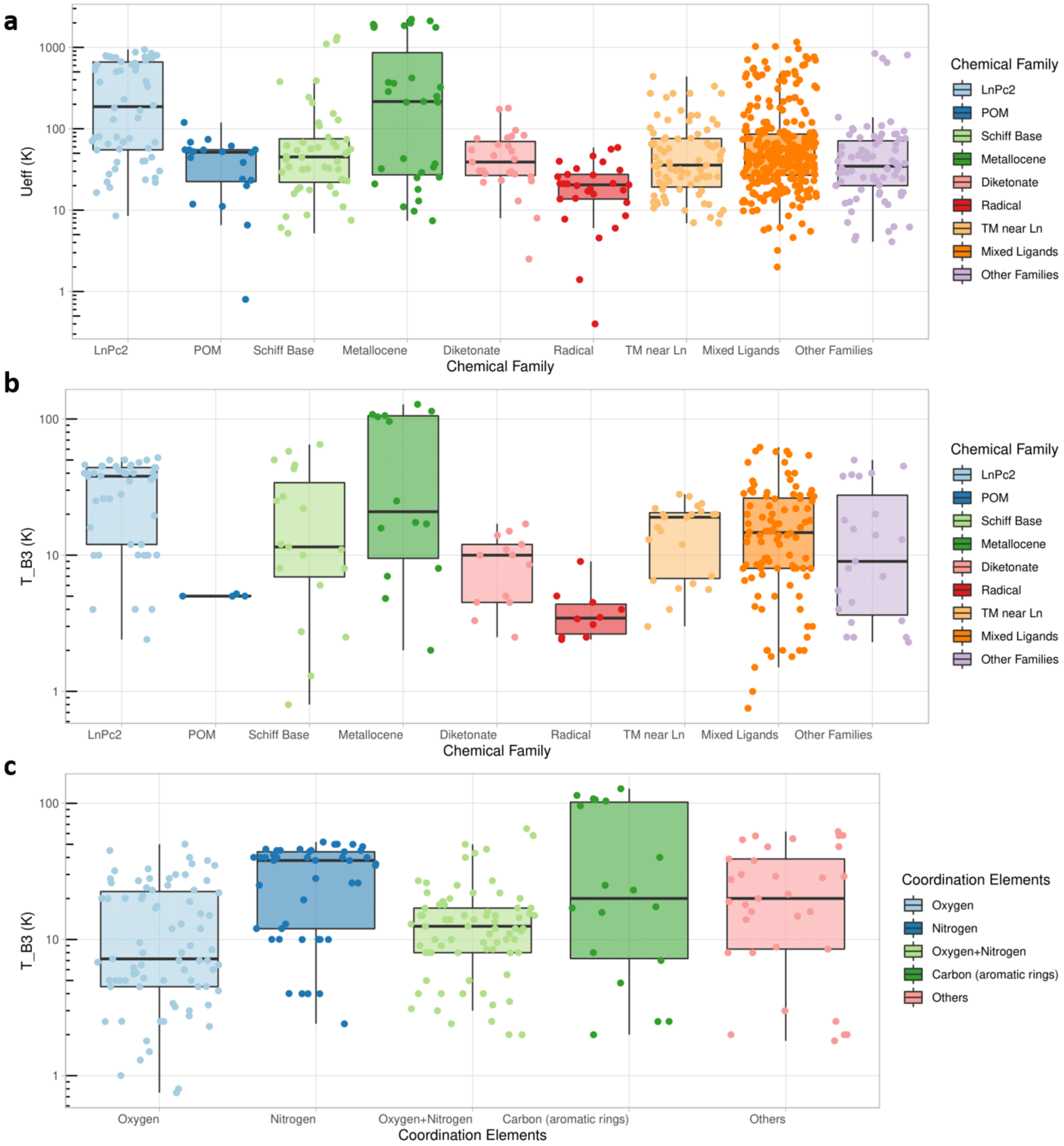

Supplementary Figure 11.1 | Boxplots of physical variables *vs* chemical variables. a, U_{eff} *vs* chemical family. **b**, T_{B3} *vs* chemical family. **c**, T_{B3} *vs* coordination elements.

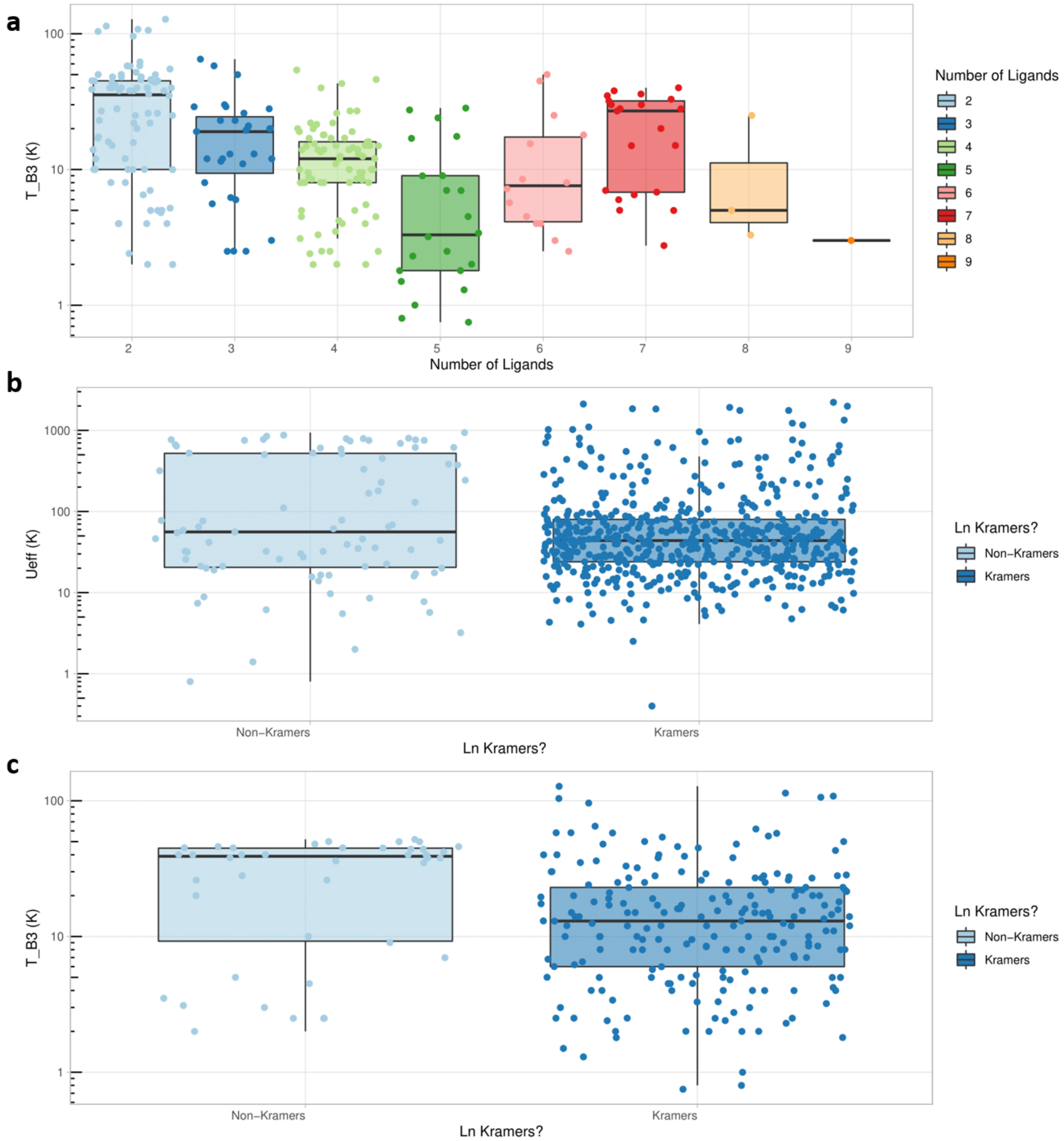

Supplementary Figure 11.2 | Boxplots of physical *vs* chemical variables. a, T_{B3} *vs* number of ligands. **b**, U_{eff} *vs* spin parity of the metal ion. **c**, T_{B3} *vs* spin parity of the lanthanide ion.

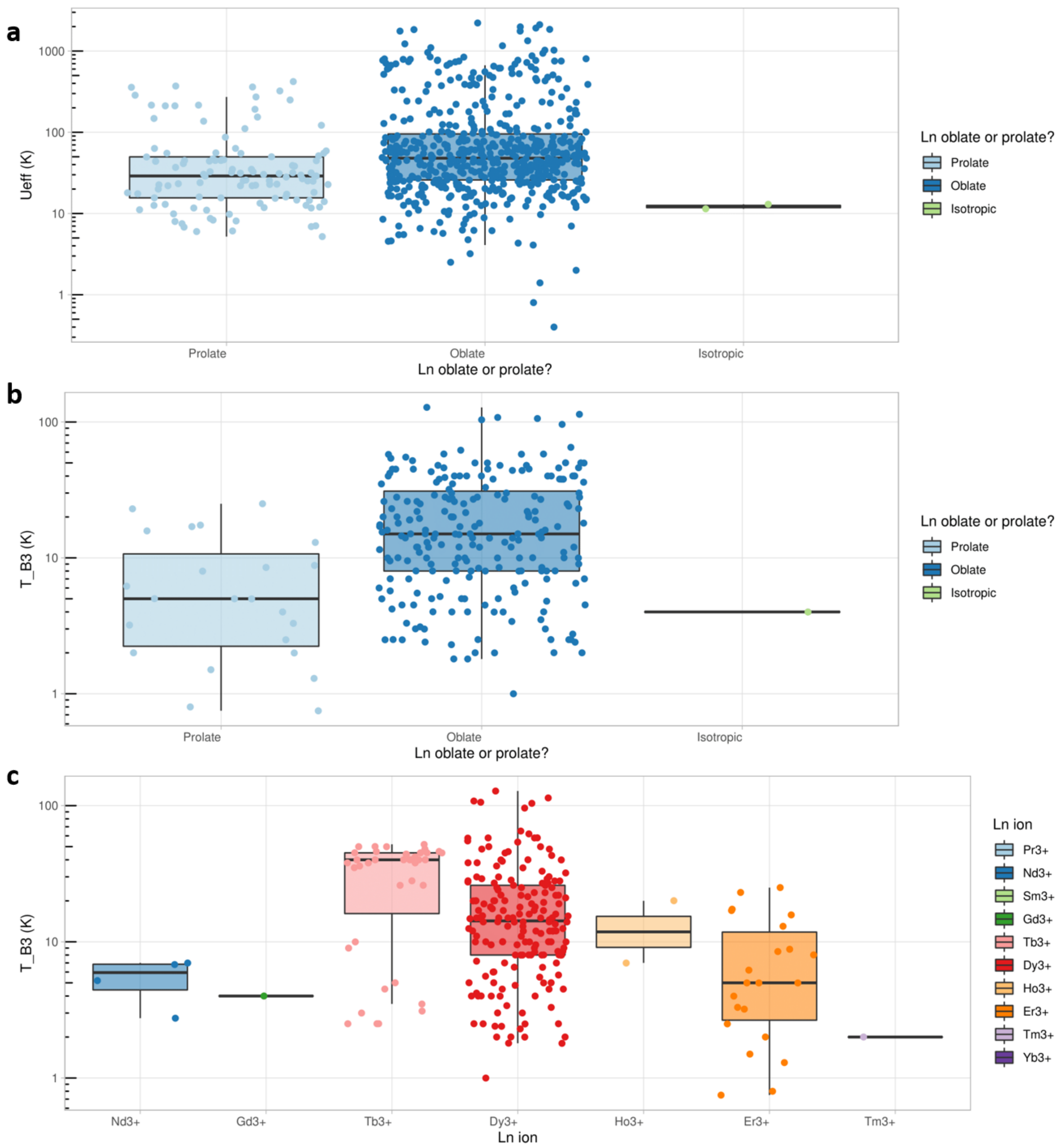

Supplementary Figure 11.3 | Boxplots of physical vs chemical variables. a, U_{eff} *vs* anisotropy of the lanthanide ion. **b**, T_{B3} *vs* anisotropy of the lanthanide ion. **c**, U_{eff} *vs* lanthanide ion.

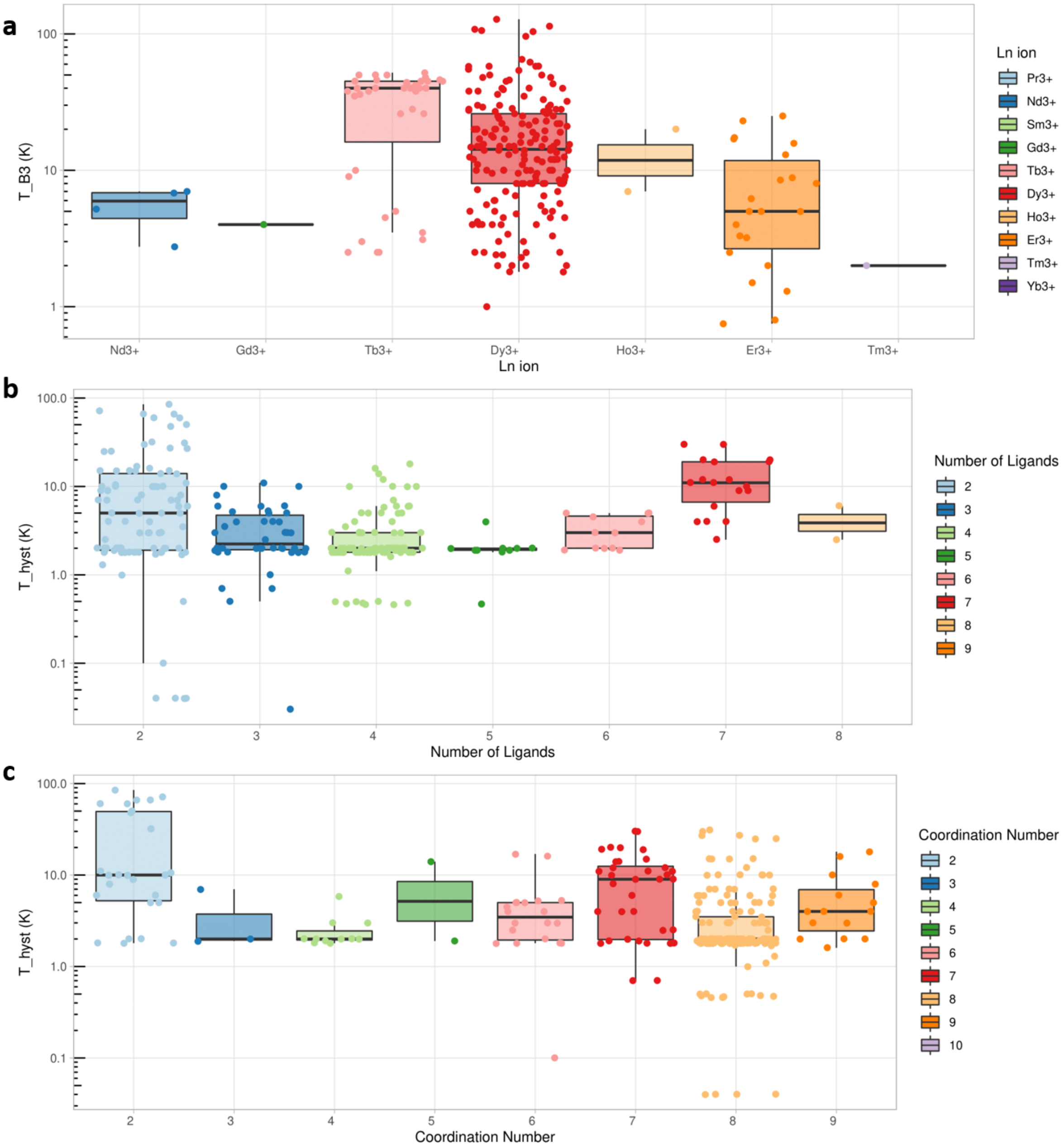

Supplementary Figure 11.4 | Boxplots of physical vs chemical variables. a, T_{B3} *vs* lanthanide ion. **b**, T_{hyst} *vs* number of ligands. **c**, T_{hyst} *vs* coordination number.

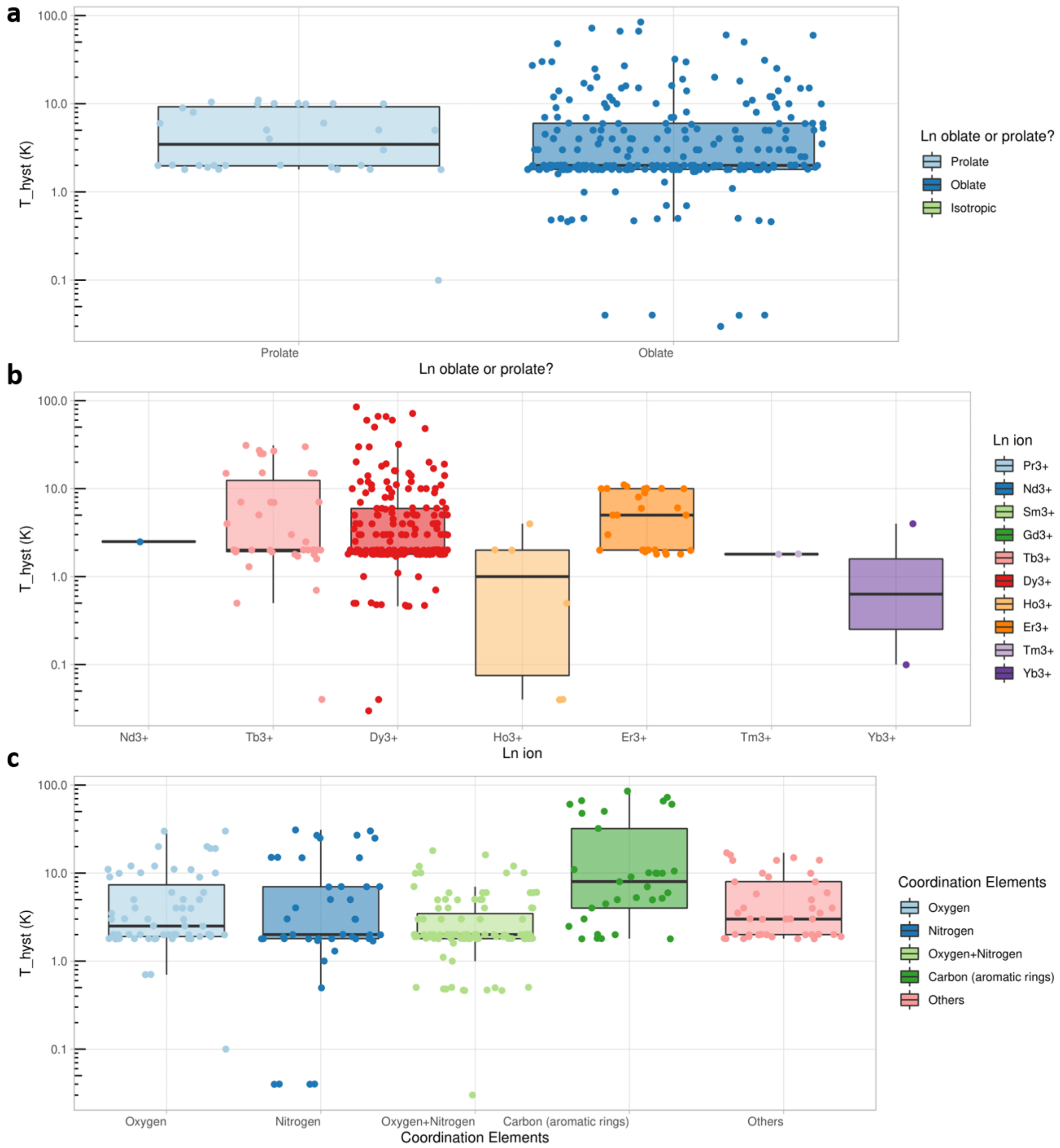

Supplementary Figure 11.5 | Boxplots of physical vs chemical variables. a, T_{hyst} *vs* anisotropy of the lanthanide ion. **b**, T_{hyst} *vs* lanthanide ion. **c**, T_{hyst} *vs* coordination elements.

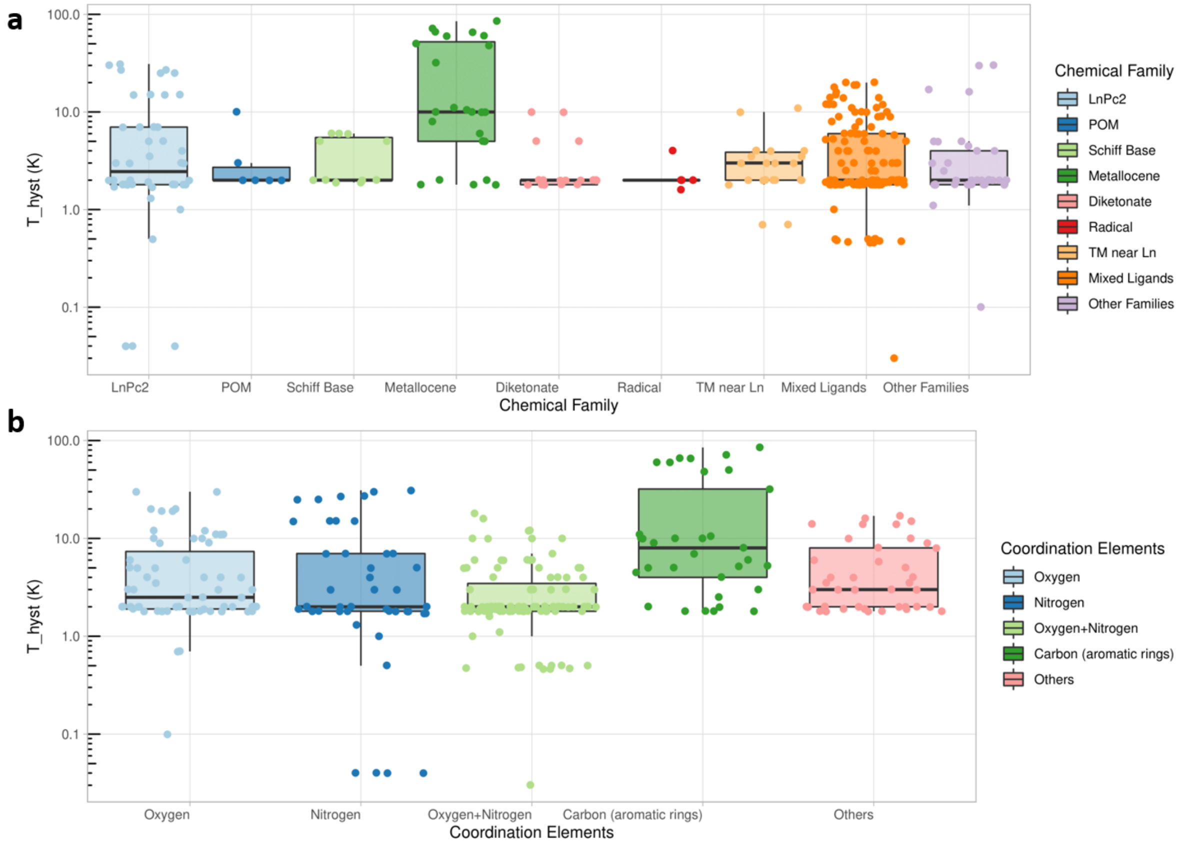

Supplementary Figure 11.6 | Boxplots of physical vs chemical variables. a, T_{hyst} *vs* chemical family. **b**, U_{eff} *vs* coordination elements.

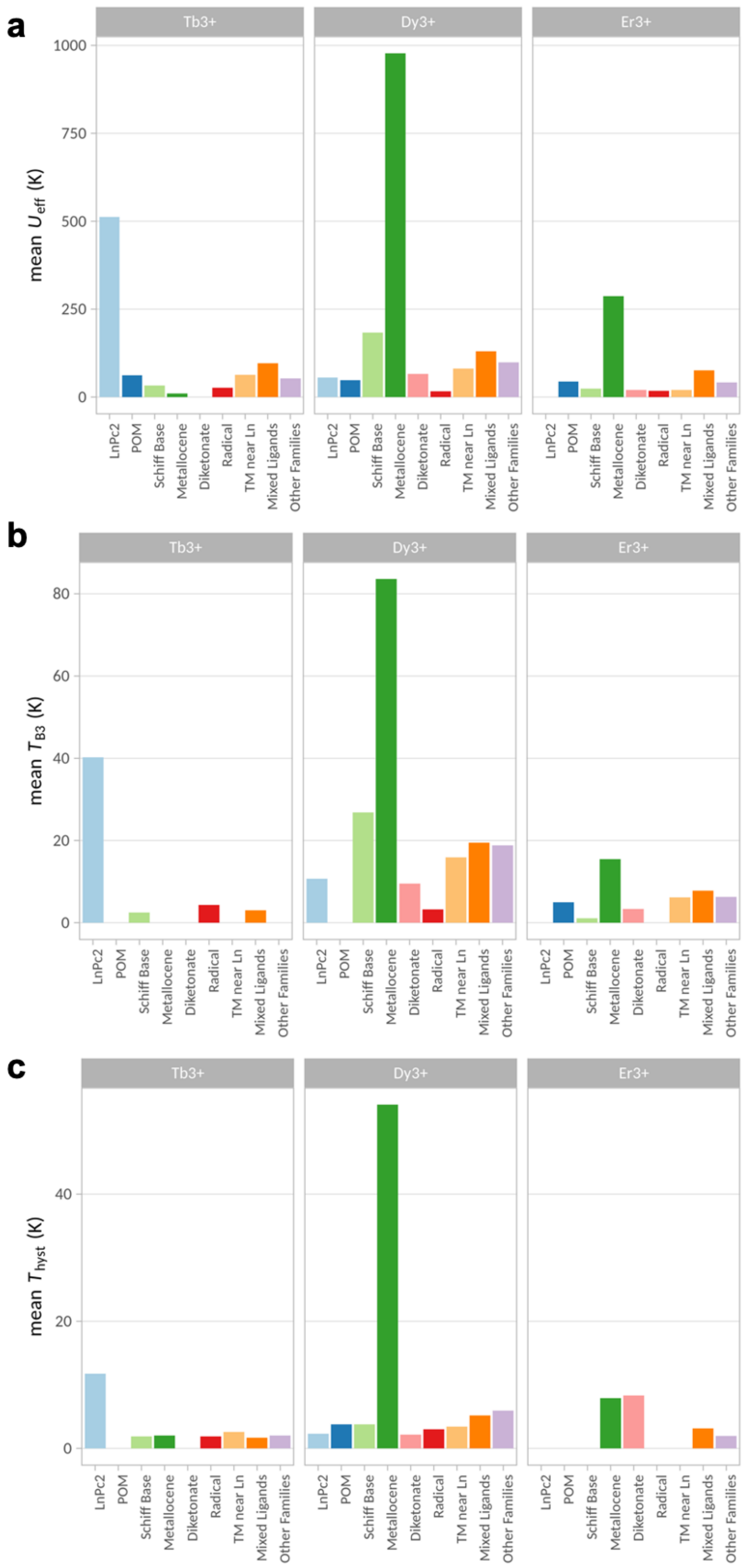

Supplementary Figure 12.1 | Bar chart representations of the relation between Ln ion, chemical family and arithmetic mean of U_{eff}, T_{B3} and T_{hyst}. Bar charts showing the mean

values for every combination of categories between the main metal ions {Tb^{3+}, Dy^{3+}, Er^{3+}} and all chemical categories. **a**,U_{eff}, **b**, T_{B3}, **c**, T_{hyst}.

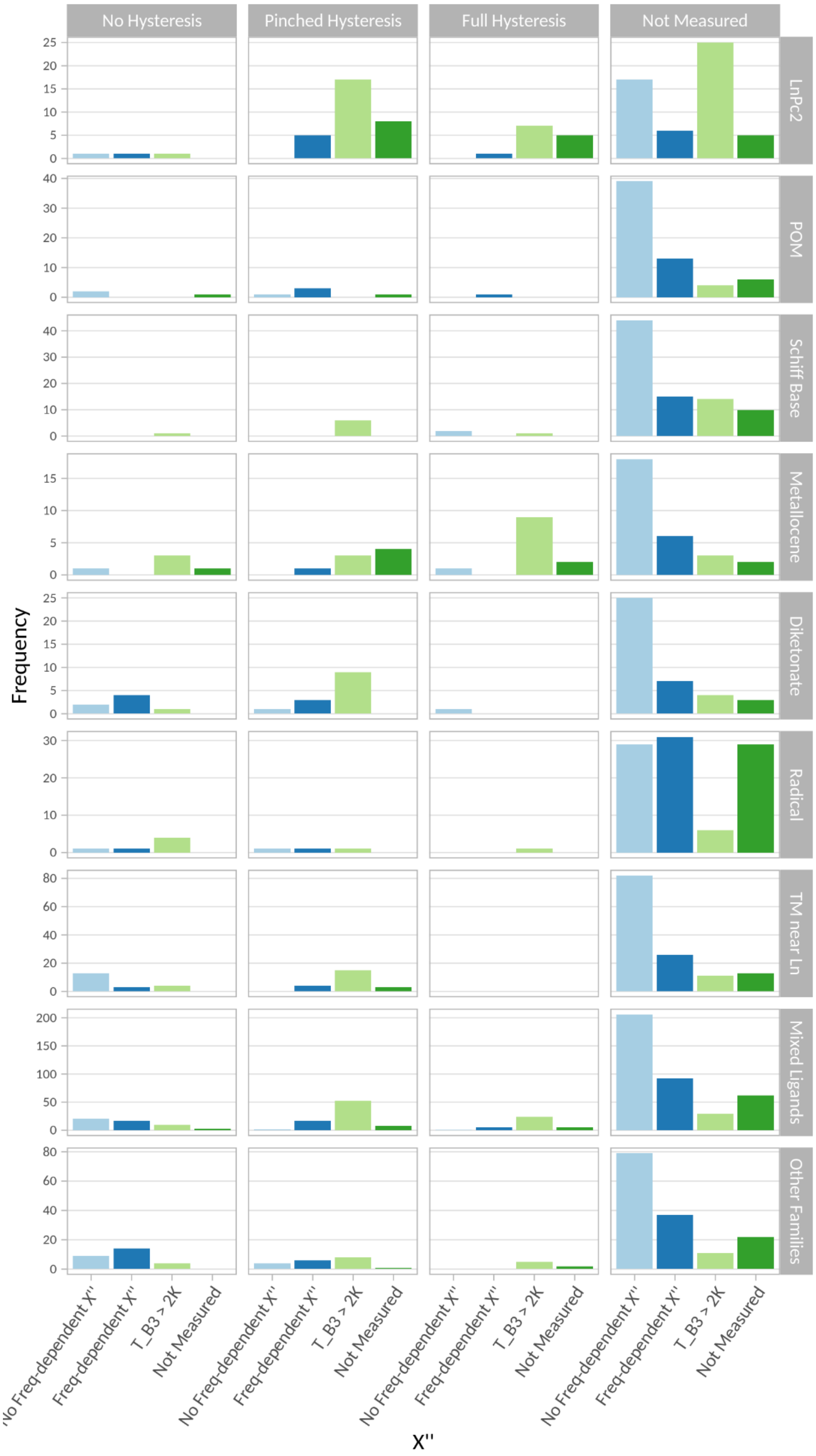

Supplementary Figure 12.2 | Bar chart representations of the relation between chemical family and magnetization dynamics. Bar charts showing the frequency of samples for every combination of categories between the categorical variables "chemical family", "Hyst" and "χ''_{max}". Graphs are normalised to the maximum frequency in each chemical family.

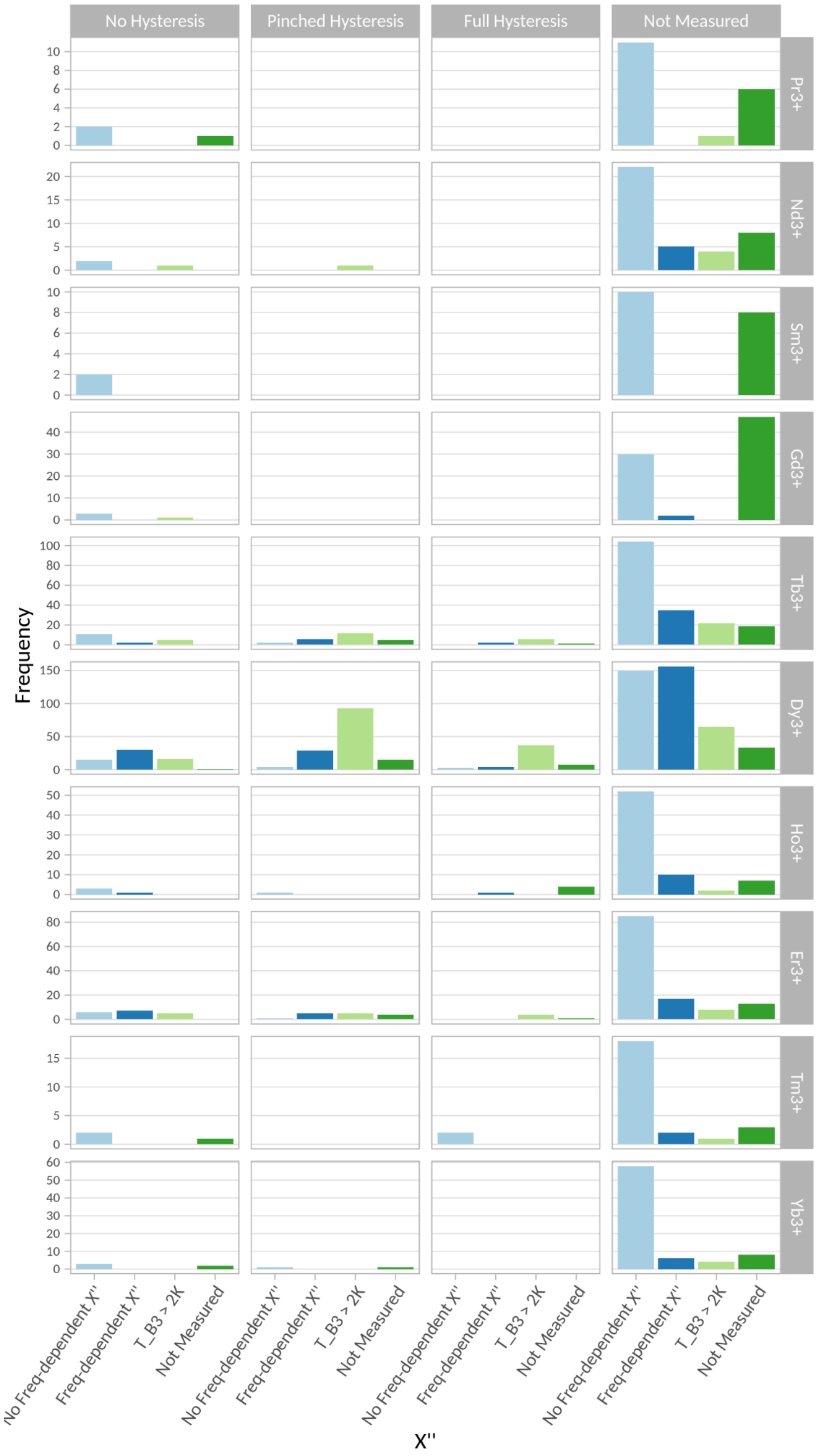

Supplementary Figure 12.3 | Bar chart representations of the relation between lanthanoid ion and magnetization dynamics. Bar charts showing the frequency of samples for every combination of categories between the categorical variables "lanthanoid ion", "Hyst" and "χ''_{max}". Graphs are normalised to the maximum frequency in each chemical family.

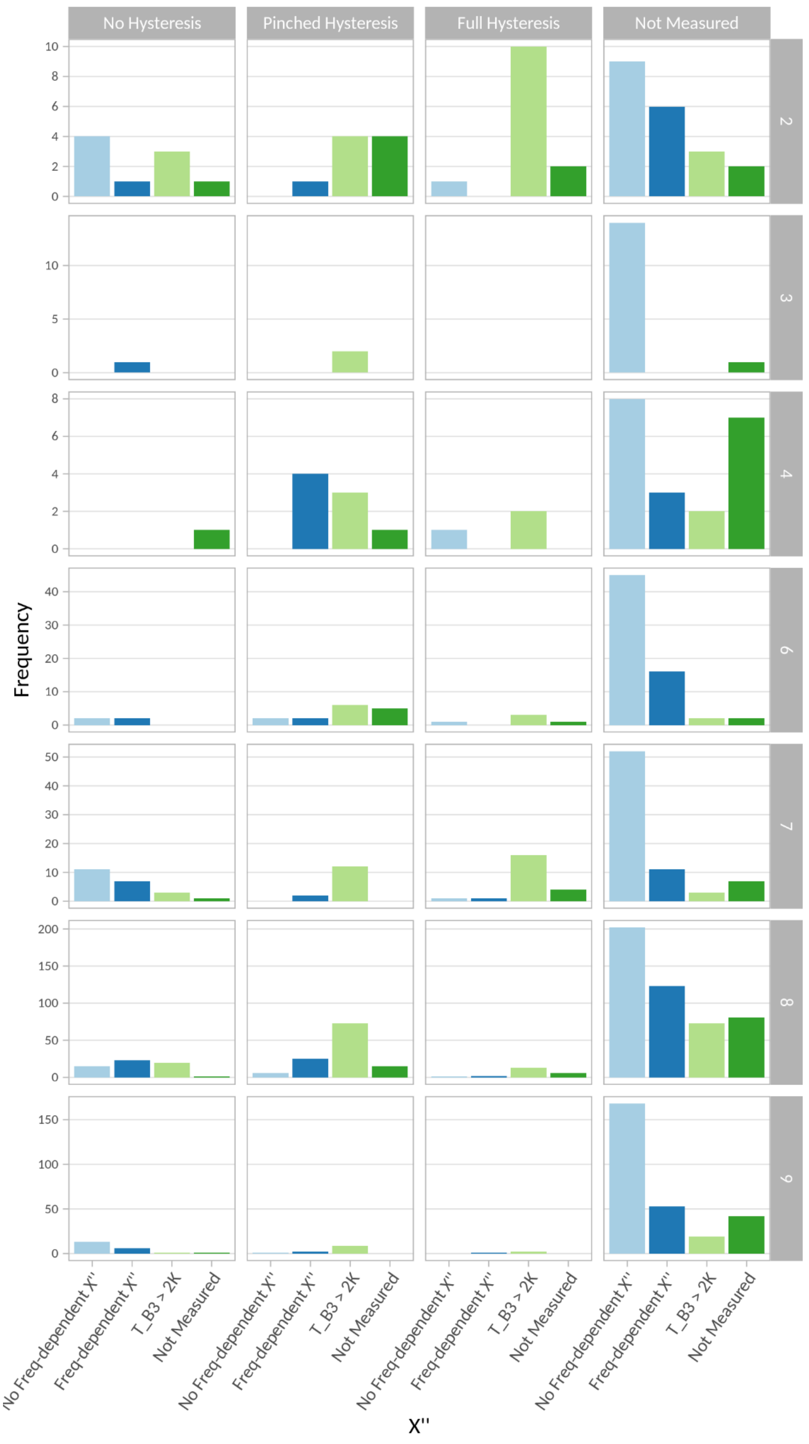

Supplementary Figure 12.4 | Bar chart representations of the relation between coordination number and magnetization dynamics. Bar charts showing the frequency of samples for every combination of categories between the categorical variables “coordination number”, “Hyst” and “χ''_{max}”. Graphs are normalised to the maximum frequency in each chemical family.

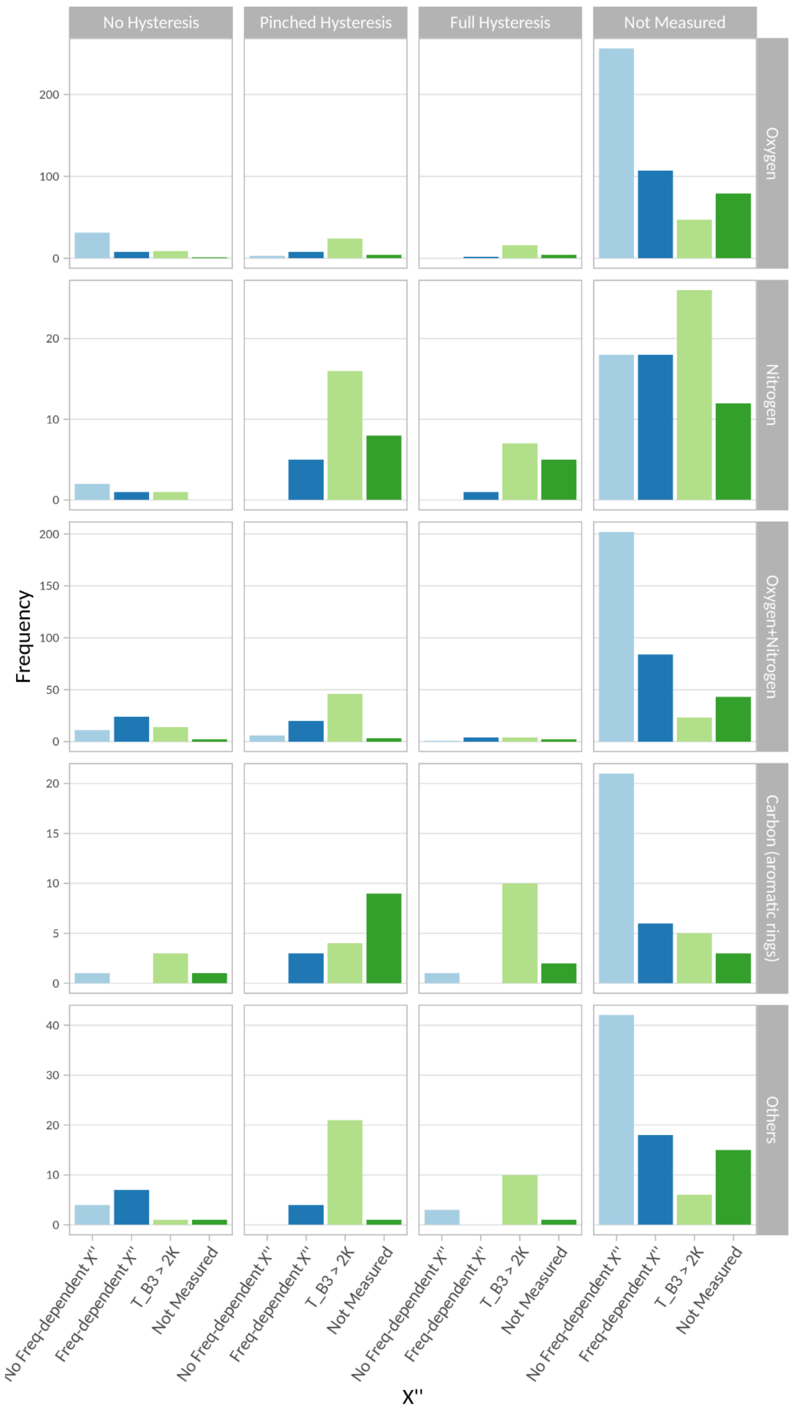

Supplementary Figure 12.5 | Bar chart representations of the relation between coordination elements and magnetization dynamics. Bar charts showing the frequency of samples for every combination of categories between the categorical variables "coordination elements", "Hyst" and "χ''_{max}". Graphs are normalised to the maximum frequency in each chemical family.

3.2. Extended gallery of SIMDAVIS graphs: Arrhenius equation parameters

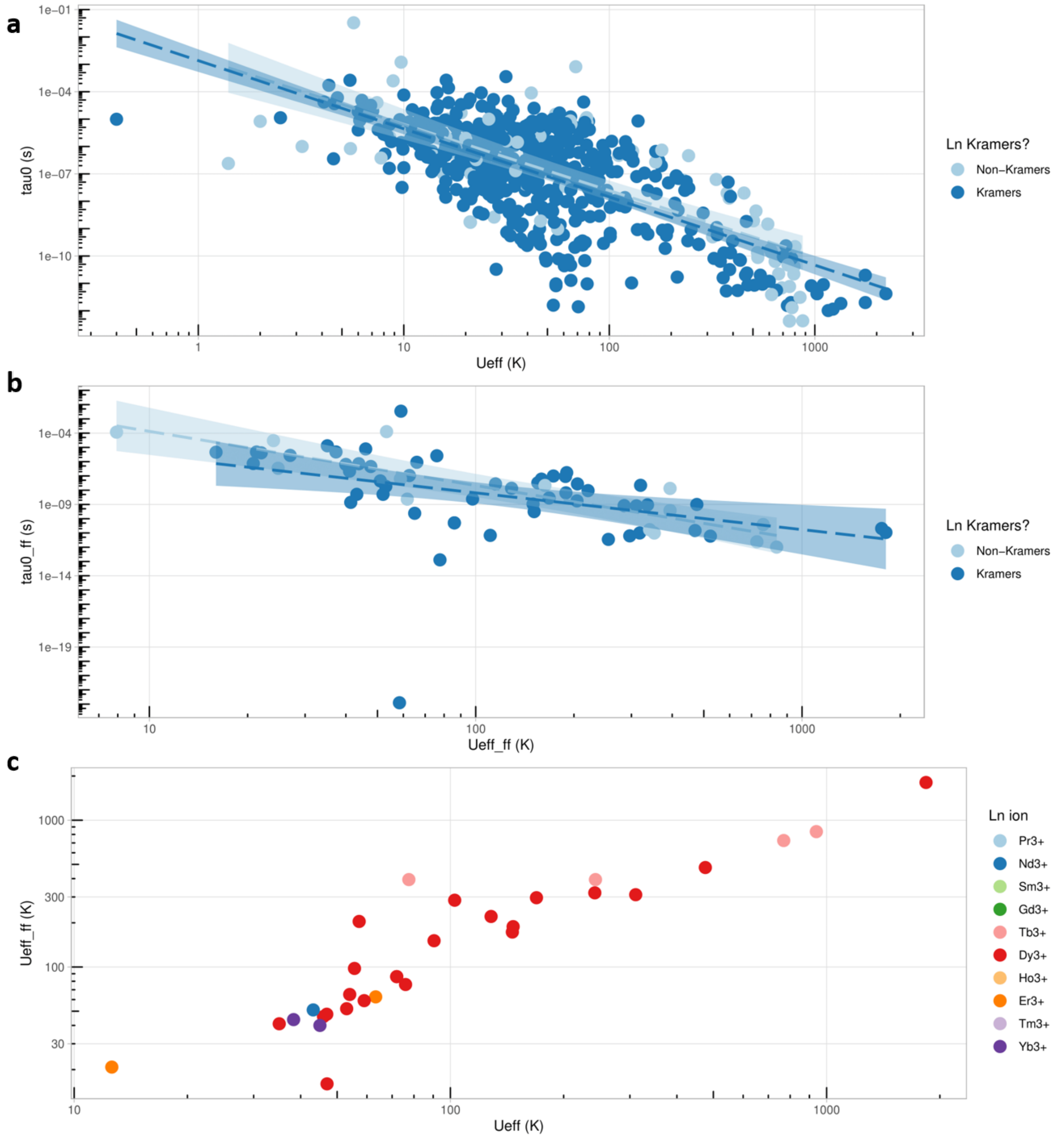

Supplementary Figure 13.1 Scatterplot representations of the relation between U_{eff}, $U_{\mathrm{eff,ff}}$, τ_0, $\tau_{0,\mathrm{ff}}$. **a**, τ_0 vs U_{eff}, or Kramers and non-Kramers ions, with linear regressions; **b**, $\tau_{0,\mathrm{ff}}$ vs $U_{\mathrm{eff,ff}}$, for Kramers and non-Kramers ions, with linear regressions; **c**, $U_{\mathrm{eff,ff}}$ vs U_{eff} for different lanthanide ions, with a visible linear behaviour.

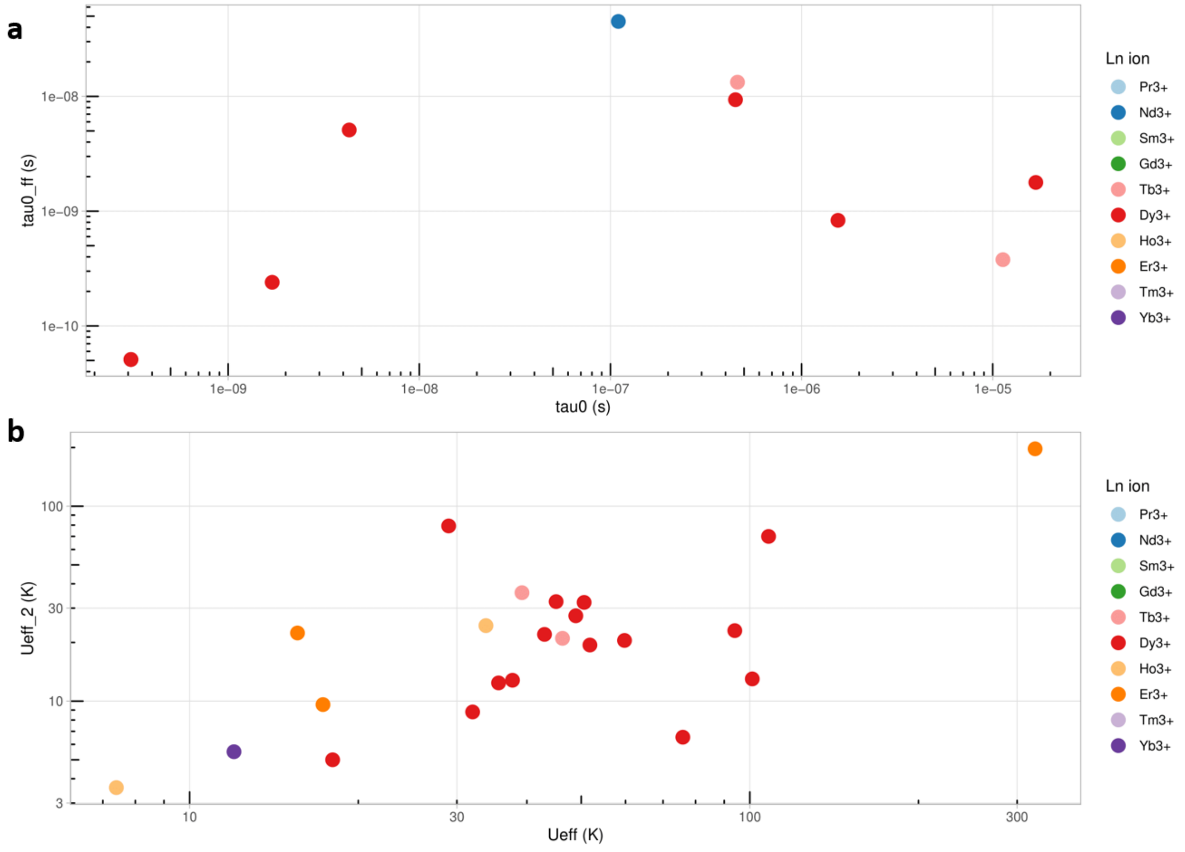

Supplementary Figure 13.2 | Scatterplot representations of $\tau_{0,ff}$, $U_{eff,2}$ vs τ_0, $U_{eff,ff}$. a,: $\tau_{0,ff}$ *vs* τ_0 for different lanthanide ions, with no discernible relation between the parameters; **b**, $U_{eff,2}$ *vs* U_{eff} for different lanthanide ions, with no discernible relation between the parameters.

Supplementary Section 4. Statistical analysis of the chemical variables

All qualitative results presented in this section considered the whole dataset containing data from literature from 2003 to 2019 for the analyses of chemical variables that follow. Additionally, we repeated the study with the data subset in the timeframe 2003-2017 (~1000 samples instead of ~1400), and the conclusions were robust, with no difference resulting from whether one considers the whole data set from 2003-2019 or only 2003-2017 subset. All quantitative numbers given herein are also consistent between the two studies, within a 5% difference. We can therefore conclude that the relations among the chemical variables are stable, *i.e.* no new trend has been revealed since 2017.

4.1. Initial multiple correspondence analysis

Here we are striving to determine the existing statistical correlations among the chemical variables in the studied sample. This is necessary in order to avoid being misled later on by meaningless correlations between chemical design variables and physical behaviour. For example, we find that the variables “number of ligands” and “coordination elements” happen to be strongly correlated with each other, then it is likely that they will both display the same correlations with a given physical behaviour. In particular, one can expect that an “all-nitrogen coordination environment” will be strongly correlated with “number of ligands = 2”, and with “chemical family = $LnPc_2$”, and relatively few other complexes in the dataset present only nitrogens as donor atoms. Therefore, if one obtains a correlation between a desirable physical behaviour and “chemical family = $LnPc_2$”, it would be unwarranted to deduce that this behaviour can be obtained solely by employing an all-nitrogen coordination environment, or solely by preparing complexes with 2 ligands.

Correspondence Analysis (CA) or reciprocal averaging is a multivariate statistical technique that is employed for the graphical analysis of the dependence or independence of a set of categorical variables from data in a contingency table. It consists in summing up the information in the rows and columns so that it can be projected on a reduced subspace, and represent simultaneously the row and the column data, allowing to obtain conclusions about each pair of variables.

CA only requires data to be organised in categories. Since in our case there are more than two variables, we employed Multiple Correspondence Analysis (MCA). Different approaches for MCA have been proposed; we employed the widely used Gifi system.[54] This system consists of a set of multivariate methods developed around the Alternating Least Squares (ALS) algorithm. Among these methods, Homogeneity Analysis provides a model that is equivalent to MCA. ALS’s solution for Homogeneity Analysis is known as HOMALS. We employed the R homals package to obtain the following graphical representations.[55] Results are plotted in Supplementary Figs. 14, 15 and 16.

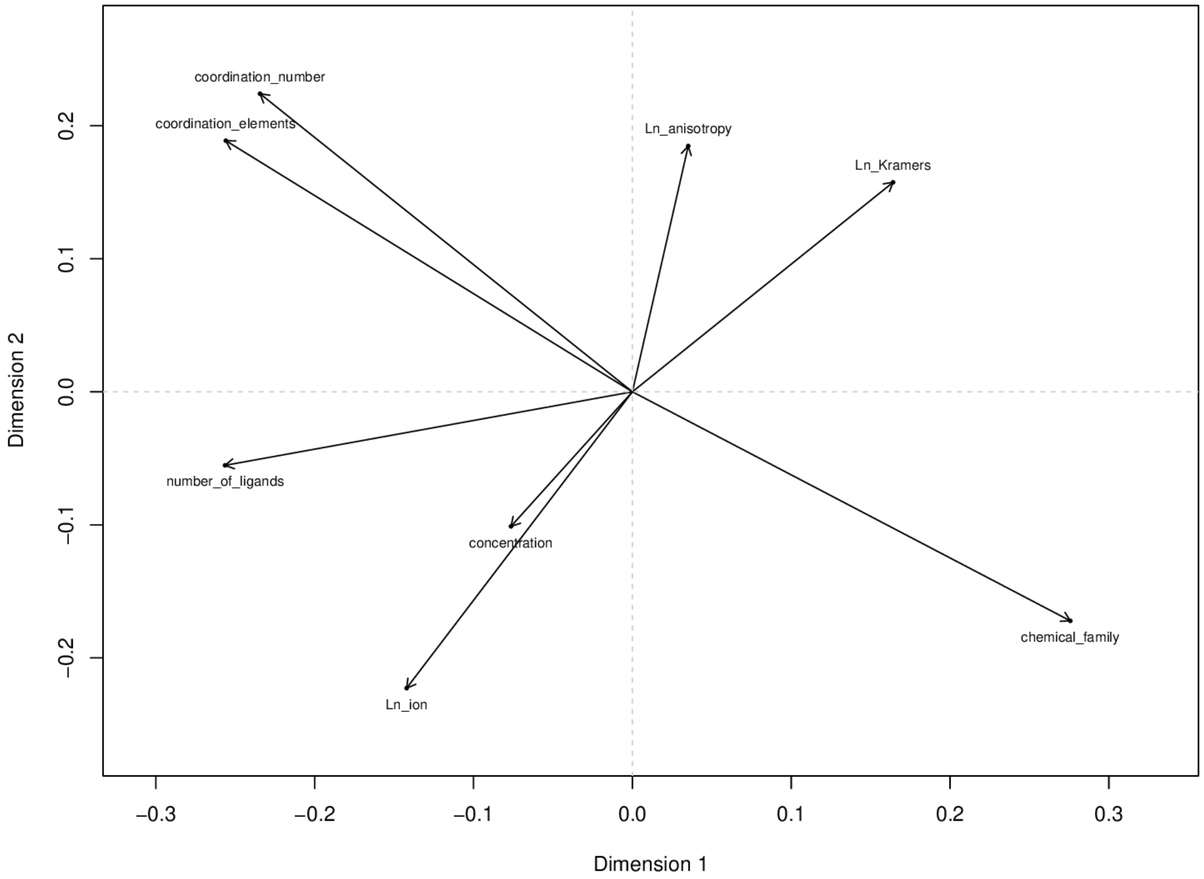

Supplementary Figure 14 | Multiple Correspondence Analysis: minimal representation of each of the variable loadings on the two main dimensions.

The graph in Supplementary Fig. 14 is read as follows: (i) the length of the vector approximates the variation within each variable, (ii) the cosine between two vectors approximates the correlation between two variables, *i.e.* parallel vectors correspond to perfectly correlated variables, (iii) the distance between the endpoints of two vectors approximates the dissimilarity between the two variables, (iv) the projection of each vector allows to order the data points for that variable. These two MCA dimensions will be employed to understand further analysis, in particular clustering studies. Supplementary Figures 15 and 16 are complementary to Supplementary Fig. 14, and allow for a more complete understanding.

We employed the R ade4 package[56] for MCA, which only allows the analysis of categorical variables, and obtained the results collected in Supplementary Table 1 for the correlation ratio of each variable.

Supplementary Table 1 | Correlation ratio of each chemical variable with the two MCA dimensions.

	RS1	RS2
chemical_family	0.81096423	0.6147058
Ln_ion	0.20607706	0.5364464
Ln_anisotropy	0.05097696	0.3121107
Ln_Kramers	0.16243450	0.2918366
coordination_number	0.62057925	0.3528815
number_of_ligands	0.47815893	0.1020615
coordination_elements	0.73301849	0.5353323

To achieve some clarity in the following data-rich representations, we assigned numerical labels to all categorical values as indicated in Supplementary Section 1. Employing these labels and within the same package we obtained the following complementary boxplot representation (Supplementary Fig. 15) that allows us to see the distribution of values, for each variable, along the axis defined by the MCA dimension 1. It is easy to see that the most extreme negative values of MCA dimension 1 are displayed by samples that have carbon or nitrogen as coordination elements, 2 ligands, CN = 2 or 3, as well as those of the metallocene chemical family. As significant positive values in MCA dimension 1, one needs to highlight Gd^{3+}, as well as isotropic complexes which are obviously the same ones.

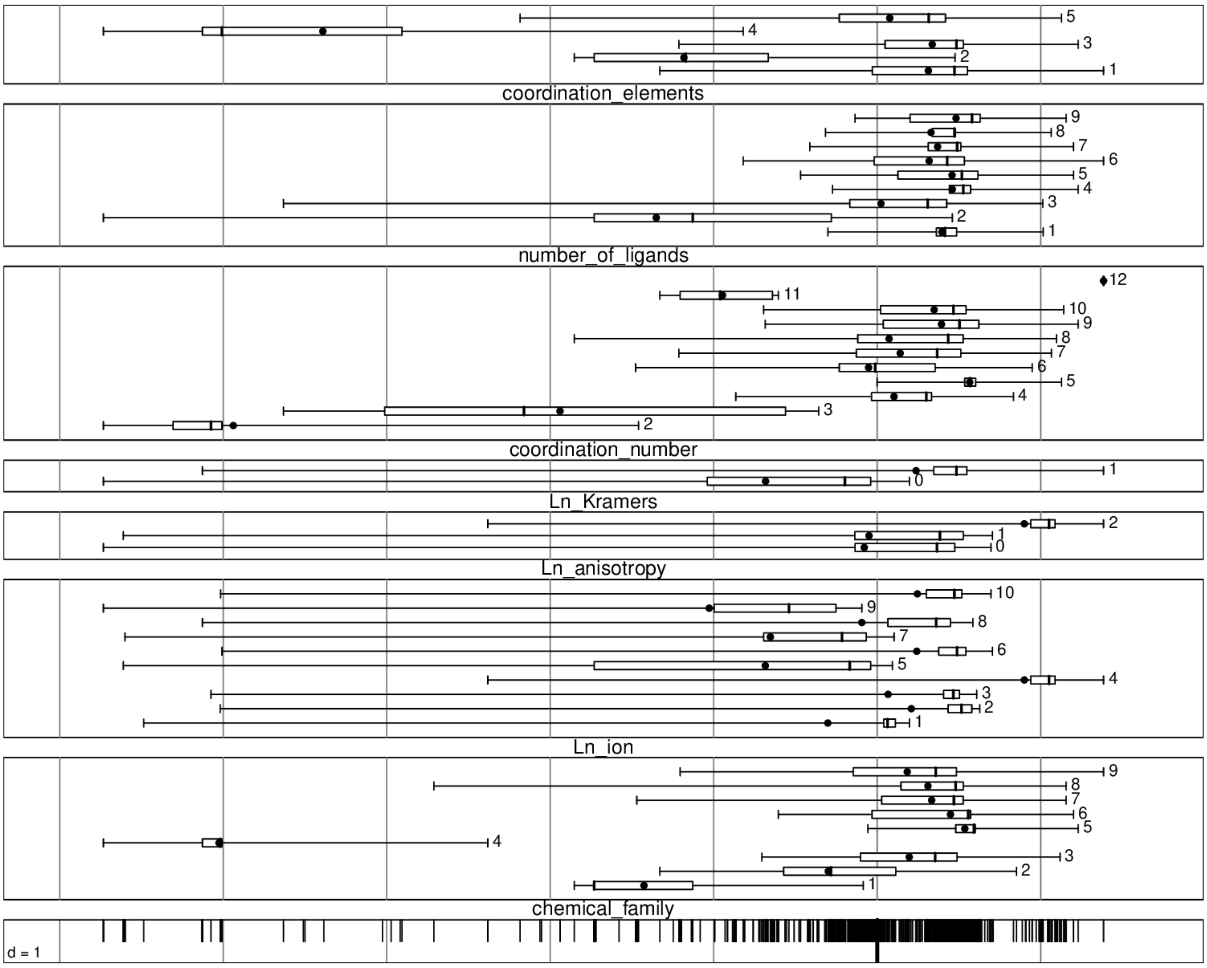

Supplementary Figure 15 | Boxplots for the distribution along the first MCA dimension of the different categorical values for the chemical variables. See the numbering convention for the categories of the variables in Supplementary Section 1.

A further alternative representation, depicting the distribution of values for the different variables in the dataset as subsets of points was also done (Supplementary Fig. 16). This allows to locate the different values for each chemical variable in terms of positive and negative value ranges for the MC dimensions 1 and 2 simultaneously. At the same time, it allows us to observe overlap between chemical variables, *e.g.* CN = 2 or 3 are in overlap with the metallocene family and with coordination by carbon. Similarly, there is a significant overlap between the $LnPc_2$ family, complexes with 2 ligands, Ln = Tb and a coordination sphere formed by nitrogen atoms. As we will see in Supplementary Section 4.2, while this kind of information points into a clear direction, specialised representations and statistical studies will allow us to study parameter association by clustering.

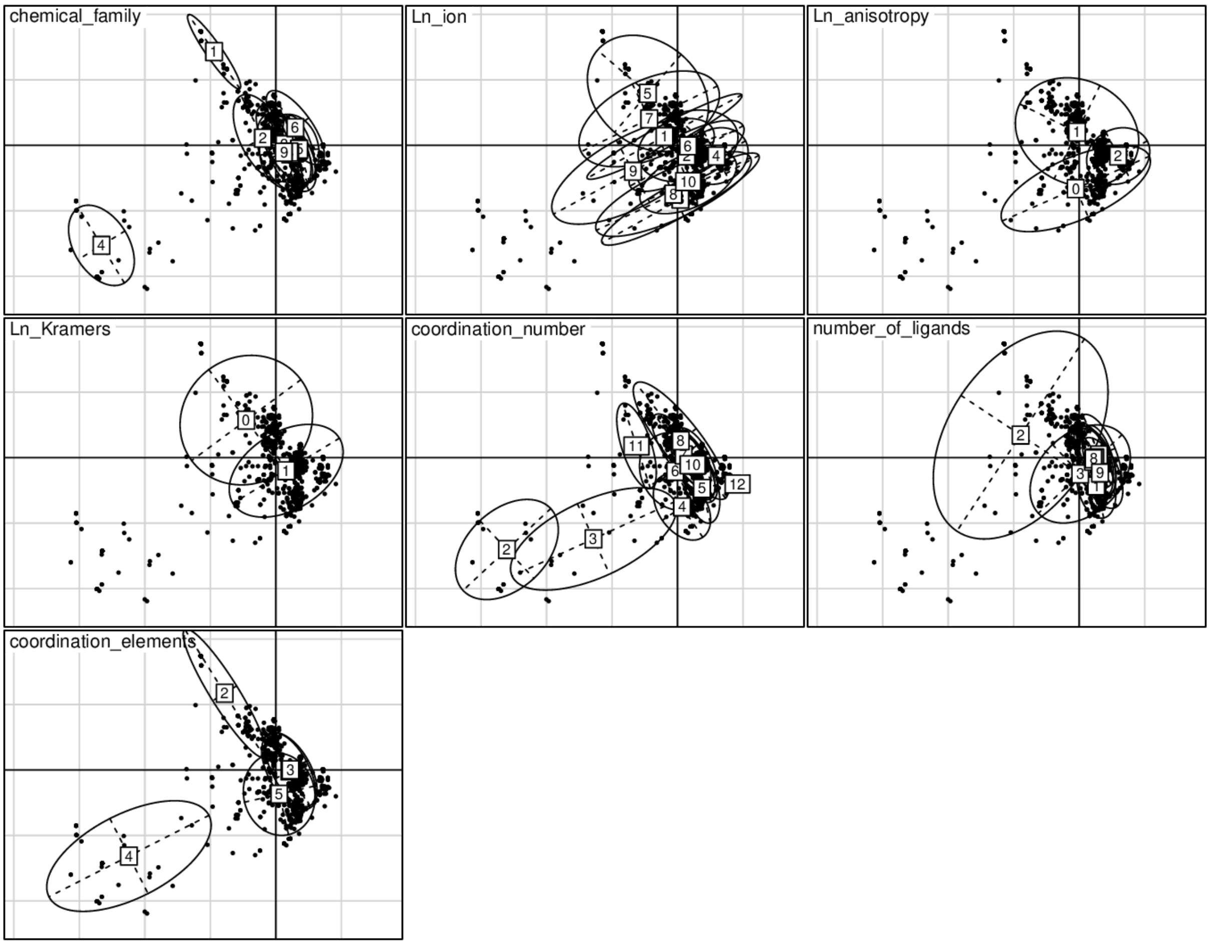

Supplementary Figure 16 | Subsets for the different values of each of the seven categorical chemical variables in the plane of the two MCA dimensions.

4.2. Clustering studies for the chemical variables

Clustering studies were performed as an independent test. In particular, we employ the package FactoMineR in R to perform the MCA.[57] This allows a graphical representation of the distances between individual samples and the relations between variables and their values. The goal in this case is to detect within the data types or profiles of data with similar characteristics. In other words, this procedure groups the values for the chemical parameter sets corresponding to individual measurements in families that present an overall similarity.

Due to the nature of our study, this is a crucial step since in practice chemical parameters are not homogeneously distributed as in a purely combinatorial approach. On the contrary, different research groups have chemical expertise in the preparation of different classes of compounds, and often, also evolving ideas on which design strategies would be more relevant for the desired physical property. This means that different research groups at different times focus on different chemical families and strategies for the molecular design of SIMs. As a result, the overall number of samples (~1400) can be judiciously divided in a small set of hierarchical clusters which share certain common features, as a kind of molecular taxonomy. Again, this will help us put in context our findings: it is to be expected that, when finding a pattern or a magneto-structural trend, we will actually be seeing the behaviour of a cluster

rather than the influence of an isolated parameter. Supplementary Figs. 17 and 18 show two different perspectives on the dendrogram resulting from the clustering.

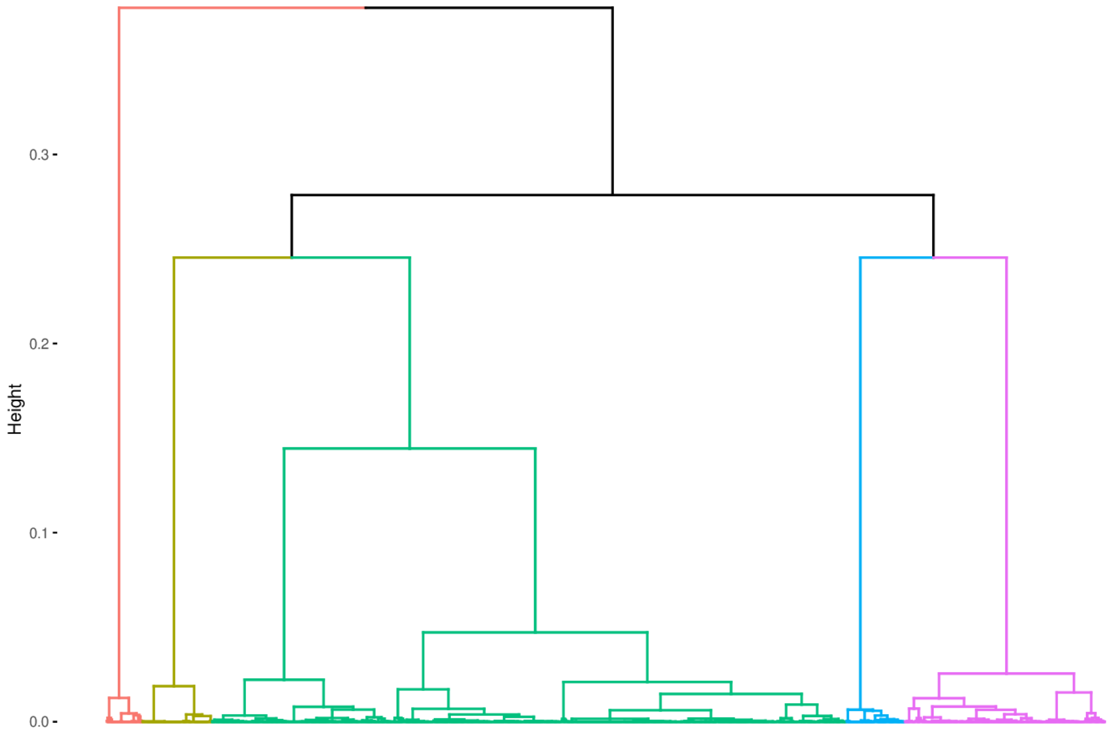

Supplementary Figure 17 | Dendrogram visualisation of the calculated hierarchical clustering on the factor map.

Depending on the height of the dendrogram cut in Supplementary Figures 17 and 18, one can obtain more or less fine-grained clusters. Our dataset contains a main categorisation with 5 tipologies A-E, described below, and we always offer an alternate, finer categorisation within the same hierarchical clustering (mol_cluster_2 in the dataset) with 7 tipologies A-F, which is also included in the Data tab of the the SIMDAVIS dashboard.

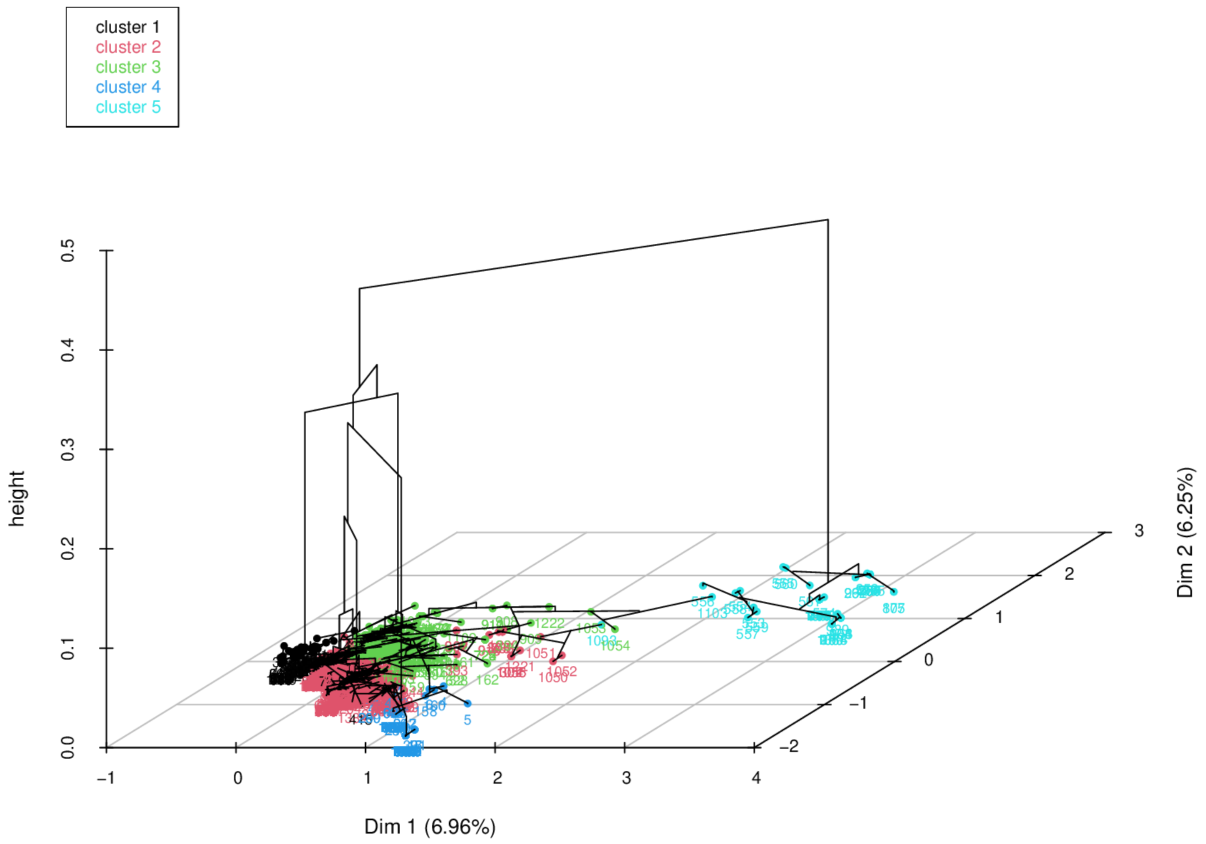

Supplementary Figure 18 | Perspective view of the dendrograms visualisation of the calculated hierarchical clustering on the factor map.

We found the following 5 tipologies (for details see Supplementary Figs. 19.1 and 19.2):

-Cluster A is small (6% of the samples) and corresponds almost perfectly with the set of Gd^{3+} compounds, or, equivalently, of isotropic complexes. Properties that are strongly overrepresented in this cluster compared to the whole sample include belonging to the "radical" chemical family, number of ligands = 5 and a coordination sphere formed by oxygen only.

-Cluster B is the largest one (63% of the samples), and is composed entirely of oblate ions. 90% of the total of all Dy^{3+} samples are inside Cluster B, however there is a large minority (33.3%) of samples inside Cluster B which are based on non-Dy^{3+} ions. Cluster B also includes ~75% of all samples of "mixed ligands", of "other families" and where the coordination sphere is a mixture of Oxygens and Nitrogens, and 70% of the samples where the coordination sphere is all Oxygen.

-Cluster C is the second largest one (20% of the samples), and it is composed almost entirely of prolate Kramers ions. Above 50% are Er^{3+}, and almost 30% are Yb^{3+}. Additionally, coordination number = 8 is strongly overrepresented in this cluster.

-Cluster D is small (7.4% of the samples), includes practically all Pc double-deckers ($LnPc_2$), and indeed corresponds very well with this chemical family. The match is weaker in the case of the categories corresponding to all Nitrogen in the coordination sphere, 2 ligands and 8 atoms in the coordination sphere, in the sense that there is a minority of samples with these

features which are outside this Cluster (Supplementary Fig. 19.2). 60% of the samples in Cluster D are based on Tb^{3+}, 30% are based on Dy^{3+}.
-Cluster E, which is the smallest (3.7% of the samples), corresponds almost perfectly to the metallocene family and, like in Cluster D, the match with all-Carbon coordination, coordination number = 2, and number of ligands = 2 is weaker since some samples with these features are outside this cluster. The samples in Cluster E are more often based on prolate than oblate ions (~40% prolate), but significantly less so than in the total set (~20% prolate). This can be related with the fact that, while ~30% of samples in Cluster D are based on Dy^{3+}, this is much less than for the total dataset (~50% Dy).

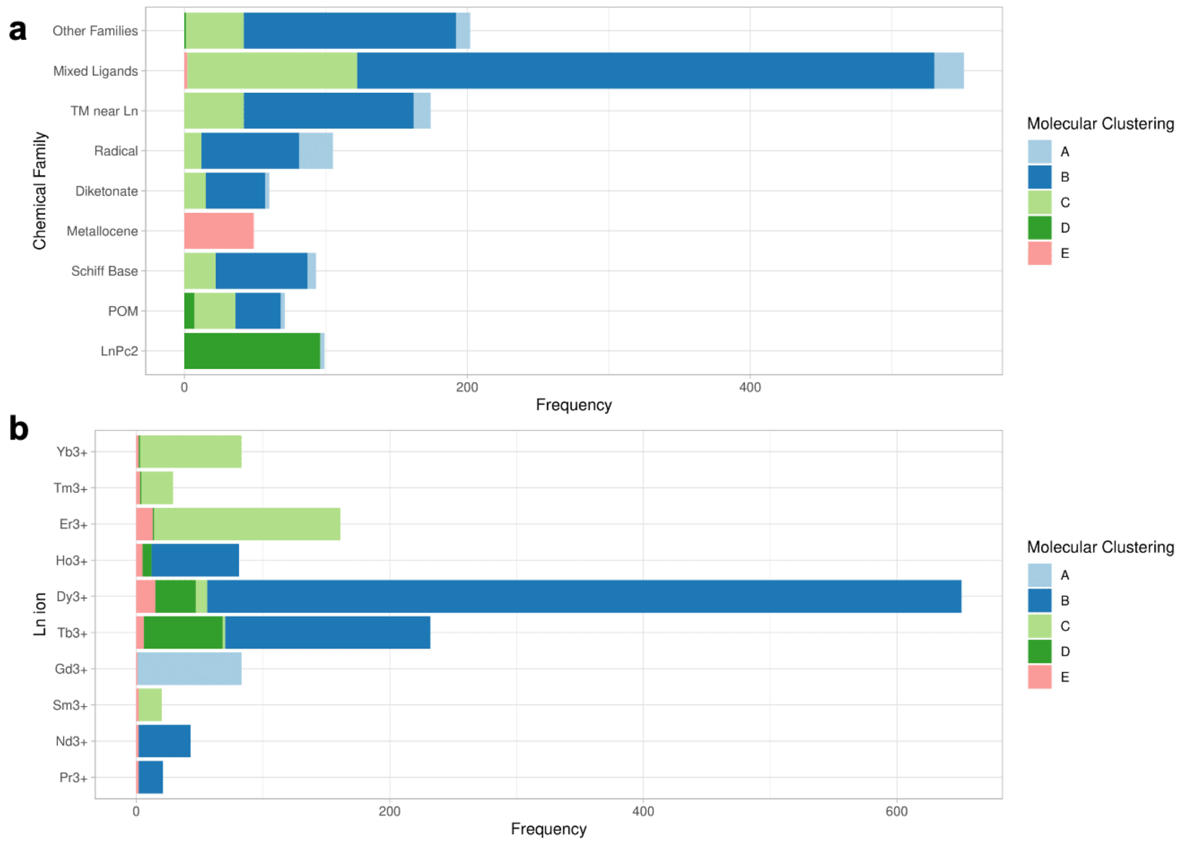

Supplementary Figure 19.1 | Bar charts representing the frequencies of chemical variables, filled according to the molecular clustering described in this section. a, chemical families, **b**, Ln ions.

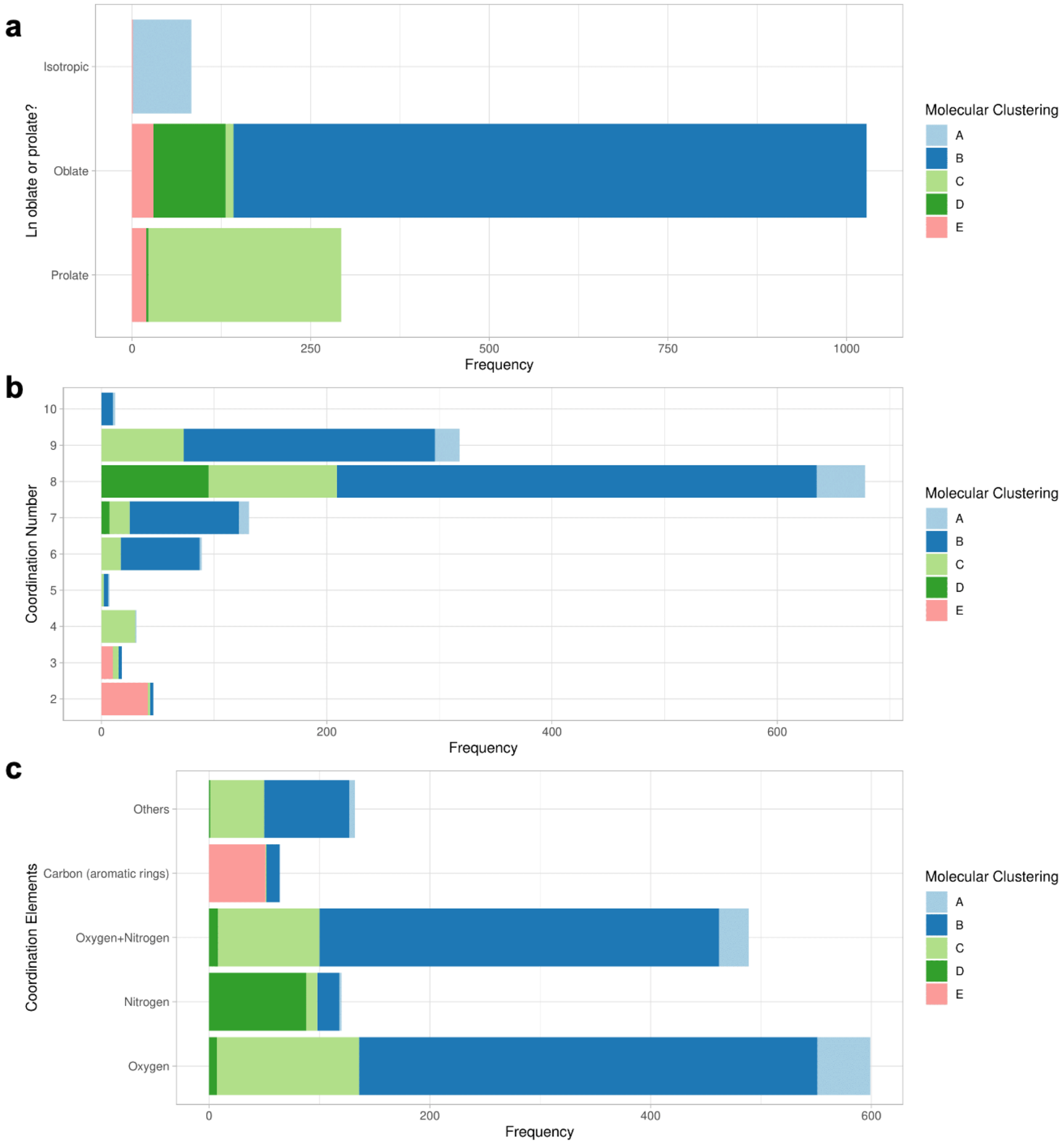

Supplementary Figure 19.2 | Bar charts representing the frequencies of chemical variables, filled according to the molecular clustering described in this section. a, oblate *vs* prolate nature of the ion, **b**, coordination numbers, **c**, coordination elements.

4.3. Lognormal modelling

Finally, we applied a lognormal model to detect possible association between variables. The different factors within the model indicate the different associations between the studied variables and parameters. This model works with the frequencies of each variable in the crosstabulation. These frequencies result from each combination of variables, so Poisson's distribution was employed. For any modelization one needs to declare the link function: a function of the expected value of the dependent variable, taking the form of a linear combination of the independent variables. For lognormal modelling, we do not distinguish between dependent and independent variables, since one is rather interested in associations between variables, but one does have a link function which unites the average frequency with the linear predictor, which in this case is the logarithmic function. Within the chemical variables we have many categorical ones, with many levels each. To avoid a useless complication of the analysis, we start by studying the relation between the lanthanide ion, its anisotropy and spin parity (Kramers or not). This serves solely as a test for the modelling and the integrity of our data, since we know that these are associated beforehand: for every lanthanide ion, its anisotropy type and Kramers nature is well defined, with no exceptions. As expected, our model found that it is sufficient to work with the lanthanide ion, and we discarded the other two variables for the subsequent clustering studies. We proceeded to use lognormal models, introducing the rest of the chemical variables, one by one, together with the lanthanide ion. We found that the chemical family, lanthanide ion and coordination elements are the main variables and sufficient to reasonably explain the frequencies of the rest of the variables.

Supplementary Section 5. Statistical analysis of the physical variables

For all the statistical analyses of the physical variables that follow in Supplementary Sections 5.1 and 5.3, additionally to the analyses performed for the complete dataset (data from years 2003-2019), we repeated the study with the data subset in the timeframe 2003-2017 (~1000 samples instead of ~1400). All qualitative results presented here are robust whether one considers the whole dataset 2003-2019 or the 2003-2017 subset. Each of the weak numerical correlations indicated in Supplementary Fig. 20 are stable within a 0.2 window, and in particular all correlations higher than 0.9 are stable with deviations within 0.05 window between both subsets. All values for the Akaike Information Criterion (AIC) in Supplementary Tables 2, 3, 4 are higher on average by about a factor of 1.5-2, as expected, since they are roughly proportional to the number of samples.[58] We can therefore conclude that the quantitative and qualitative relations among the physical variables are stable, *i.e.* no new significant trends have been revealed recently.

5.1. Overview of the main statistical relationships

Here, our goal is to identify statistical relationships among the physical variables. At first, we want to confirm whether the simple model parameters U_{eff}, $U_{\text{eff,2}}$, τ_0 are good statistical predictors (*i.e.* present a high correlation with) of the experimental behaviour. In particular, whether they are good predictors of T_{B3}, T_{B3H}, T_{hyst}, H, $\chi_{\text{im,max}}$ and "Hyst". Next, we want to determine whether the predictive power is different between U_{eff} *vs* $U_{\text{eff,ff}}$, and τ_0 *vs* $\tau_{0,\text{ff}}$. Supplementary Fig. 20 represents graphically all physical variables, with their relationships in pairs; $U_{\text{eff,2}}$ was eliminated from the graph since the scarcity of data produced errors in the correlation test.

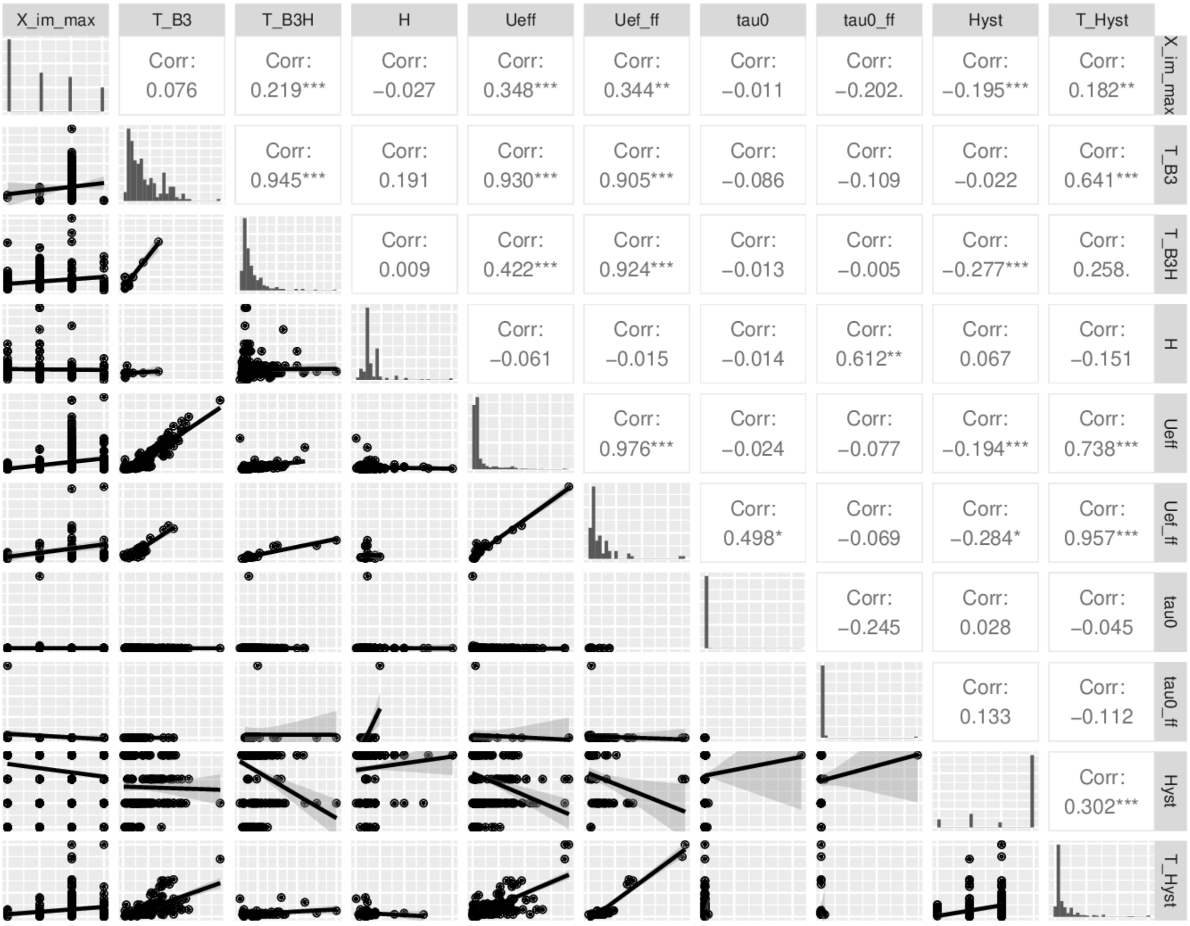

Supplementary Figure 20 | Statistical relationships among the physical variables. The diagonal shows the frequency of each value (range) for each of the physical variables. Below the diagonal we see graphical representations to visually show the relation between every pair of variables, and above the diagonal we find the quantification of each correlation, in the case of numerical variables, or an alternate boxplot, in the case of categorical variables.

Let us first go over this bidimensional array of statistical representations and analyses, and later on we will focus on the most significant pieces of information.

Keeping in mind the definition of $\chi_{\text{im,max}}$ (Supplementary Section 1), the properties as SIM are better when $\chi_{\text{im,max}}$ takes higher numbers (0 < 1 < 2), *e.g.* only the samples with $\chi_{\text{im,max}}$ = 2 present values for T_{B3}, while $\chi_{\text{im,max}}$ = 3 means having no information. The most frequent

value is $\chi_{im,max}$ = 0 (Freq-independent χ''), with the rest being similarly abundant. For T_{B3} and T_{B3H}, higher temperature values are associated with higher values of $\chi_{im,max}$. Higher effective energy barriers U_{eff}, $U_{eff,2}$, $U_{eff,ff}$ were estimated for $\chi_{im,max}$ = 3 (corresponding to cases where the SIM properties are not necessarily bad, but just were not characterised via χ'' vs T plots) and especially for $\chi_{im,max}$ = 2 (as expected), compared with systems with worse properties, where almost no difference is found between $\chi_{im,max}$ = 0 and $\chi_{im,max}$ = 1. As in the case of the effective energy barriers, higher hysteresis temperatures T_{hyst} were reported for $\chi_{im,max}$ = 3, and especially for $\chi_{im,max}$ = 2, as expected, compared with systems with worse properties.

The temperatures of the maximum in χ'' at 10^3 Hz are labelled as T_{B3} (when no magnetic field is applied) and T_{B3H} (when a magnetic field H is applied). There are more cases reported in the presence of a magnetic field. In both cases, there is a distribution similar to an inverse exponential, meaning higher temperatures are less frequent, but this is more marked for T_{B3H}: when a magnetic field is applied to detect a maximum in χ'', high temperature values for this maximum are rare. Higher values of the magnetic field H do not correlate with higher values of T_{B3H}. There is a marked difference in the correlations of the temperatures T_{B3}, T_{B3H} with the effective energy barriers U_{eff}, $U_{eff,ff}$. While there is a very strong correlation between T_{B3} and U_{eff}, for T_{B3H} the correlation only exists for $U_{eff,ff}$. This is consistent with the presence of other relaxation mechanisms in these cases, such as the QTM, which are quenched in presence of a strong field. In contrast with the effective energy barriers, there are no significant correlations between T_{B3}, T_{B3H} and τ_0, $\tau_{0,ff}$. This might be seen as puzzling, since each pair of effective energy barriers and preexponential times are extracted from a single fit. The reason for this behaviour can be understood by recalling that the search for correlation in Supplementary Figure 20 is linear, while the proper way to find the correlations in this case is via a logarithmic plot (Supplementary Section 5.3 and Supplementary Figs. 24.1 and 24.2). Since these values vary over several orders of magnitude, especially in the case of τ_0, $\tau_{0,ff}$, a minimum square root analysis over the linear data is practically determined by the small and noisy cloud of data points with maximum values rather than the whole data range. Finally, there is a systematic qualitative improvement of the hysteretic behaviour with higher values of T_{B3}, T_{B3H}. In other words, high values of T_{B3}, meaning short-term (millisecond) magnetic memory up to a high temperature, are frequently associated with the presence of hysteresis. Moreover, within cases with hysteresis, short-term magnetic memory up to a high temperature is associated with the presence of full hysteresis rather than pinched (butterfly) hysteresis. While this cannot be clearly seen in Supplementary Fig. 20, it is evident in Supplementary Fig. 21. In contrast, the quantitative correlation of the hysteretic temperature T_{hyst} with higher values of T_{B3}, T_{B3H} is relatively weak in the case of T_{B3}, and nonexistent for T_{B3H}.

H is the external magnetic field applied to measure T_{B3H}. It presents very weak (and negative) correlations with the effective energy barriers (both U_{eff} and $U_{eff,ff}$). High values of U_{eff} are visibly correlated with the sample presenting hysteresis (Hyst = 1), and within it, with samples presenting coercivity (Hyst = 2). These correlations are stronger in the case of $U_{eff,ff}$. The attempt times τ_0 and $\tau_{0,ff}$ are however very poorly correlated, both with their corresponding barriers and with the other properties. As we will see in Supplementary

Section 5.3, this is probably due to the high dispersion in values: at least a qualitative correlation is visible when one works on a logarithmic scale.

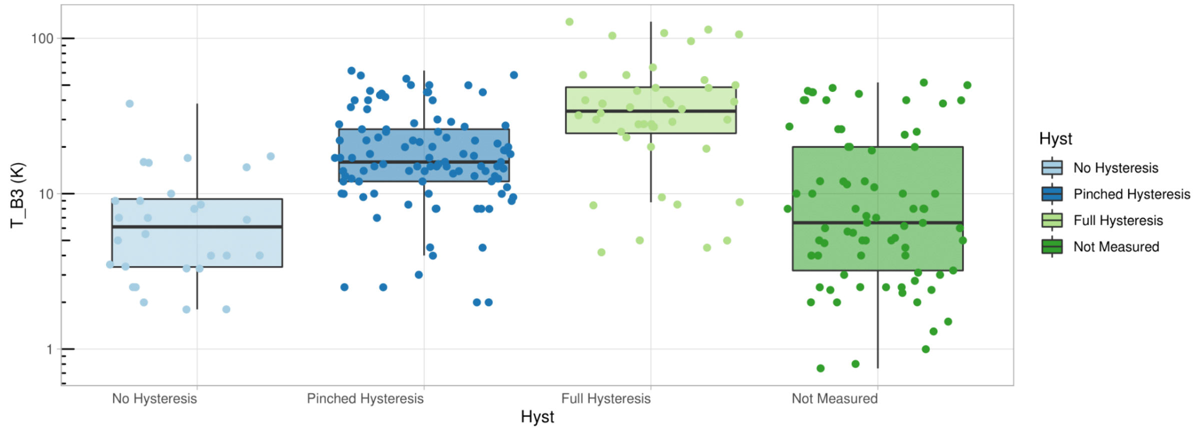

Supplementary Figure 21 | Boxplots of T_{B3} grouped by categories of hysteretic behaviour.

5.2. Simple frequency distributions

Let us start by examining the individual frequency distributions of T_{B3}, T_{B3H}, U_{eff}, $U_{eff,ff}$ and T_{hyst}, *i.e.* the five single-variable bar chart representations of their distribution of values throughout the population (diagonal graphics in Supplementary Fig. 20). In all cases, an initial visual inspection evidences a roughly inverse exponential decay, or perhaps a gaussian distribution centred around very small values. This can be interpreted as a mostly random distribution of values, a signal that the studied population is large and mostly unbiased. An interesting exception can be found in T_{B3}, which takes a bimodal distribution. This has different possible interpretations, but likely just means that a fraction of the research in the field was focused on types of compounds where the median of T_{B3} is at or above the maximum values of the other chemical families, *i.e.* the $LnPc_2$ family, see Supplementary Fig. 11.1, where it can be compared with the much less marked difference in the case of U_{eff}, where the distribution of the $LnPc_2$ family is less skewed to very high values.

5.3. Correlations between physical variables

As can be seen in Supplementary Fig. 20, the highest correlations involve U_{eff} and $U_{eff,ff}$. U_{eff} is highly correlated with T_{B3} and with T_{hyst}. $U_{eff,ff}$ is highly correlated with U_{eff}, T_{B3} and T_{B3H}. Furthermore, we apply Pearson's test to verify the correlation between U_{eff} and $U_{eff,ff}$. We obtained a very robust correlation between the two variables, with a p-value $< 2.2\cdot10^{-16}$, a 95% confidence interval for the correlation of 0.951-0.989 and an estimated correlation of 0.976. The same procedure is applied to U_{eff} and $U_{eff,2}$, obtaining results that are robust but substantially less so: p-value $< 1.113\cdot10^{-7}$, with a 95% confidence interval for the correlation of 0.701-0.941 and an estimated correlation of 0.864.

We apply the Akaike Information Criterion (AIC), a well-established method that evaluates how well a statistical model fits the data it was generated from. This method allows to compare the quality of a series of candidate models using the same data, so that the AIC

estimates the quality of each of the models relative to the others. As the models are used to represent the process that generated the data, this representation will be losing some information because of the flaws of the model, and the AIC estimates the relative amount of information lost by each candidate model. This means, the preferred model will have the lowest AIC value in a given set of candidate models. To implement AIC, we employ the R functions lm, glm with family = binomial (stats package from R base)[59], and multinom (nnet package).[60] The results can be found in Supplementary Tables 2, 3 and 4.

Supplementary Table 2 | AIC modelling experimental physical (response) variables as a function of modelling variables $\boldsymbol{U_{eff}}$, $\boldsymbol{U_{eff,2}}$, $\boldsymbol{\tau_0}$.

Response variable	Data points	Variables included in the model	Significant variable	AIC
χ''_{max}	23	U_{eff}, $U_{eff,2}$, τ_0	-	55.04
		**$\boldsymbol{U_{eff}}$, $\boldsymbol{U_{eff,2}}$**	-	49.04
		U_{eff}, τ_0	-	65.52
		$U_{eff,2}$, τ_0	-	55.62
T_{B3}	4	-	-	-
T_{B3H}	14	U_{eff}, $U_{eff,2}$, τ_0	τ_0	2.64
		U_{eff}, $U_{eff,2}$	-	35.35
		U_{eff}, τ_0	τ_0	0.98
		$U_{eff,2}$, τ_0	τ_0	4.25
		**$\boldsymbol{\tau_0}$**	τ_0	2.27
Hyst	23	U_{eff}, $U_{eff,2}$, τ_0	-	44.49
		U_{eff}, $U_{eff,2}$	-	40.49
		U_{eff}, τ_0	-	40.56
		**$\boldsymbol{U_{eff}}$**	-	36.56
		$U_{eff,2}$, τ_0	-	43.13
T_{hyst}	4	-	-	-

Supplementary Table 2 contains the analysis based on the modelling variables U_{eff}, $U_{eff,2}$ and τ_0. The analysis quantifies the models employing these three variables to explain the different response variables: {χ''_{max}, T_{B3}, T_{B3H}, Hyst, T_{hyst}}. The results are heterogeneous. Depending on the response variable chosen, the best modelling variables can be either {U_{eff}, $U_{eff,2}$}, or τ_0, or U_{eff}. Since very few samples are modelled considering two independent barriers (U_{eff}, $U_{eff,2}$), the data are scarce and the results are not statistically significant.

Supplementary Table 3 | AIC modelling experimental physical (response) variables as a function of modelling variables $\boldsymbol{U_{eff}}$, $\boldsymbol{\tau_0}$.

Response variable	Data points	Variables included in the model	Significant variable	AIC
χ''_{max}	608	U_{eff}, τ_0	-	1381.05
		$\boldsymbol{U_{eff}}$	-	1378.16
		τ_0	-	1542.43
T_{B3}	186	U_{eff}, τ_0	U_{eff}	693.98
		$\boldsymbol{U_{eff}}$	U_{eff}	692.98
		τ_0	-	1051.61
T_{B3H}	261	U_{eff}, τ_0	U_{eff}	770.73
		$\boldsymbol{U_{eff}}$	U_{eff}	768.74
		τ_0	-	818.99
Hyst	601	U_{eff}, τ_0	-	40.56
		$\boldsymbol{U_{eff}}$	U_{eff}	36.56
		τ_0	-	42.04
T_{hyst}	134	U_{eff}, τ_0	U_{eff}	650.3
		$\boldsymbol{U_{eff}}$	U_{eff}	648.32
		τ_0	-	780.94

Supplementary Table 3 contains the analysis including U_{eff} and τ_0, *i.e.* when an Orbach-only model with a single energy barrier is considered, and quantifies the models employing these variables to explain different response variables: $\{\chi''_{max}$, T_{B3}, T_{B3H}, *Hyst*, $T_{hyst}\}$. The results are very robust in this case. U_{eff} is consistently found to be the significant variable and the one producing the lowest AIC value.

Supplementary Table 4 | AIC modelling experimental physical (response) variables as a function of modelling variables $U_{\mathrm{eff,ff}}$, $\tau_{0,\mathrm{ff}}$.

Response variable	Data points	Variables included in the model	Significant variable	AIC
χ''_{max}	68	$U_{\mathrm{eff,ff}}$, $\tau_{0,\mathrm{ff}}$	-	169.58
		**$U_{\mathrm{eff,ff}}$**	-	165.33
		$\tau_{0,\mathrm{ff}}$	-	185.89
T_{B3}	27	**$U_{\mathrm{eff,ff}}$, $\tau_{0,\mathrm{ff}}$**	$U_{\mathrm{eff,ff}}$, $\tau_{0,\mathrm{ff}}$	88.5
		$U_{\mathrm{eff,ff}}$	$U_{\mathrm{eff,ff}}$	91.51
		$\tau_{0,\mathrm{ff}}$	-	137.25
T_{B3H}	23	$U_{\mathrm{eff,ff}}$, $\tau_{0,\mathrm{ff}}$	$U_{\mathrm{eff,ff}}$	74.62
		**$U_{\mathrm{eff,ff}}$**	$U_{\mathrm{eff,ff}}$	73.54
		$\tau_{0,\mathrm{ff}}$	-	116.81
Hyst	67	$U_{\mathrm{eff,ff}}$, $\tau_{0,\mathrm{ff}}$	-	137.57
		**$U_{\mathrm{eff,ff}}$**	-	131.57
		$\tau_{0,\mathrm{ff}}$	-	156.68
T_{hyst}	36	$U_{\mathrm{eff,ff}}$, $\tau_{0,\mathrm{ff}}$	$U_{\mathrm{eff,ff}}$	102.08
		**$U_{\mathrm{eff,ff}}$**	$U_{\mathrm{eff,ff}}$	102.72
		$\tau_{0,\mathrm{ff}}$	-	191.68

Supplementary Table 4 contains the same analysis but including only $U_{\mathrm{eff,ff}}$ and $\tau_{0,\mathrm{ff}}$, *i.e.* a more complete model that should be closer to reality. The modelling employing $U_{\mathrm{eff,ff}}$ is almost as good as in the case of U_{eff}. However, since information about $U_{\mathrm{eff,ff}}$ is available in fewer samples, U_{eff} is statistically preferable.

To visualise these correlations, see Supplementary Figs. 22 and 23. The relations between U_{eff}, τ_0, χ''_{max}, T_{B3H} are represented in Supplementary Fig. 22. Within a wide dispersion, a (log-log) inverse linear relation is apparent between (log-log) U_{eff} and τ_0 (more on this on Supplementary Figs. 24.1 and 24.2) and a (log-log) linear relation is apparent between T_{B3H} and U_{eff}. These graphs also show how samples with higher values of U_{eff} systematically present a maximum in T_{B3}, and in contrast samples where no frequency-dependent χ'' is measured tend to display lower values of U_{eff}. To complete the picture, we represent the relations between U_{eff}, *Hyst*, T_{hyst}, T_{B3}. (Supplementary Fig. 23). A (log-log) linear tendency is apparent when plotting T_{B3} *vs* U_{eff}; this is obscured in the case of T_{hyst} *vs* U_{eff} by the abundance of samples where the hysteresis was characterised only at 2 K. Like in the case of the ac susceptibility, there is a large dispersion of behaviours by samples with longer magnetic memory, in this case meaning the ones presenting whole hysteresis, tend to be grouped around higher values of U_{eff}, with samples presenting no hysteresis tend to present lower values of U_{eff} and samples with pinched (butterfly) hysteresis presenting typically intermediate values.

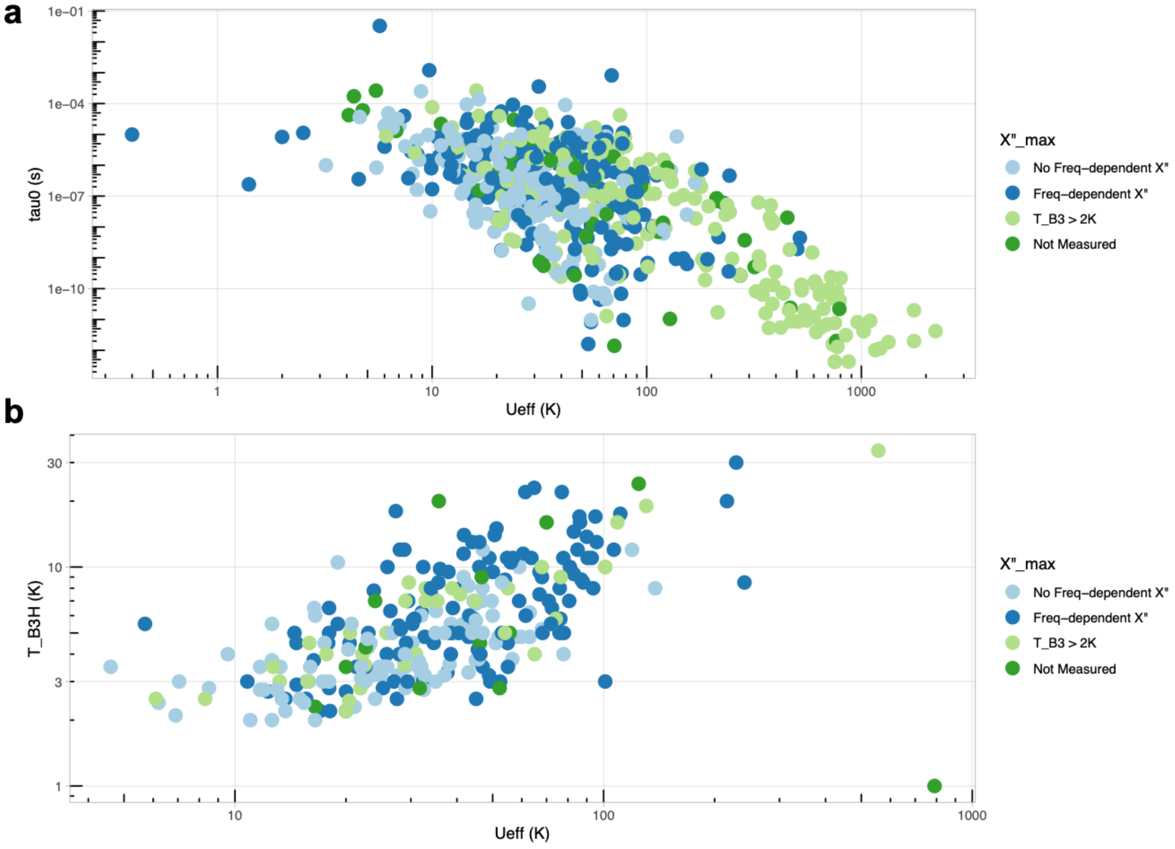

Supplementary Figure 22 | Scatterplots depicting the relation of U_{eff} with τ_0, χ''_{max}, T_{B3H}. τ_0 *vs* U_{eff}, colored by χ''_{max}, **a**; and T_{B3H} *vs* U_{eff}, colored by χ''_{max}, **b**. Note that not all samples will be present in all graphs (see Supplementary Figure 1.1). As a consequence, an (x vs y) plot can only include samples for which x and y are simultaneously present in the dataset.

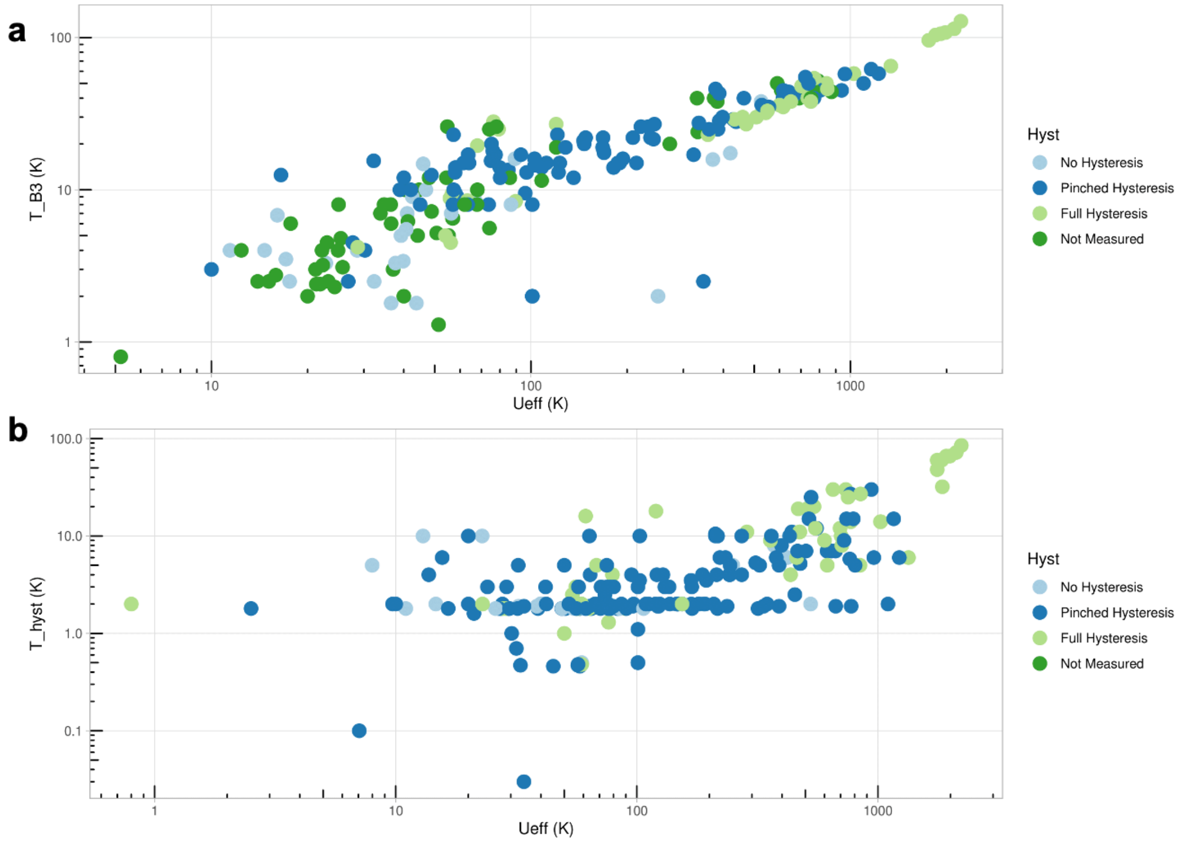

Supplementary Figure 23 | Scatterplots depicting the relation of U_{eff} with *Hyst*, T_{hyst}, T_{B3}. T_{B3} *vs* U_{eff}, colored by Hyst, **a**; T_{hyst} *vs* U_{eff}, colored by Hyst, **b**. Note that not all samples will be present in all graphs (see Supplementary Figure 1.1). As a consequence, an (x vs y) plot can only include samples for which x and y are simultaneously present in the dataset.

The main conclusion of the study is that U_{eff} derived from a simplistic Arrhenius plot is currently the best single predictor for physical behaviour. This means that, whether we are discussing in terms of the presence of maximum in out-of-phase component of the ac susceptibility (χ''_{max}) or the temperature of said maximum (T_{B3}, T_{B3H}), U_{eff} is a better predictor than τ_0 or $U_{eff,2}$ (note that the number of studies with $U_{eff,2}$ is too small). Also the number of studies deriving $U_{eff,ff}$ from a full fit considering the other physical processes is very low. Furthermore, the correlation between $U_{eff,ff}$ and U_{eff} is very high. The combination of the two facts mean that there is no statistical argument for the qualitative observation that $U_{eff,ff}$ from a full fit is a better predictor for T_{hyst}.

5.4 The question of U_{eff} vs $U_{eff,ff}$

A crucial issue is to quantify up to what level the value of U_{eff} and τ_0 are well correlated with the slow relaxation of the magnetisation, or to determine whether one would need to employ $U_{eff,ff}$ instead. Let us proceed with increasing the order of complexity. A visual inspection in

SIMDAVIS shows that, in a few cases where there is simultaneous information on U_{eff} and $U_{eff,ff}$, their values are very similar (Fig. 4a in the main text). Furthermore, this partial information is corroborated by the very similar dependencies of T_{B3} or T_{hyst} *vs* either U_{eff} or $U_{eff,ff}$, as well as in the numerical correlations (see Supplementary Sections 3.2 and 5.3). A categorical analysis (Figs. 4b, c) shows that the data dispersion is large, meaning that it is impossible to predict the experimental behaviour for an individual sample merely from its U_{eff} value. However, it demonstrates that, as expected, samples which present a maximum in the out-of-phase susceptibility χ", or hysteresis, also present higher U_{eff} values. A more thorough numerical analysis (see Supplementary Section 6) confirms these trends.

An in-depth statistical analysis of all physical parameters based on the Akaike Information Criterion (see Supplementary Section 5.3) concludes that U_{eff} derived from a simple Arrhenius plot is the best single predictor for the magnetic behaviour in our dataset. This idea was previously proposed by Ding *et al.*[61] but studied with a much lower sample size. This means that, whether we are discussing in terms of the out-of-phase component of the ac susceptibility or magnetic hysteresis, U_{eff} is a better predictor than τ_0, $\tau_{0,ff}$, $U_{eff,2}$ and, in practice, than $U_{eff,ff}$. Factorial analysis of mixed data (see Supplementary Section 6) also reveals the predictive power of U_{eff} compared with τ_0. Note that this does not contradict previous studies which demonstrated that a variation in the Orbach barrier does not fully explain the differences in retention of magnetisation,[1] since we did not explicitly consider other relaxation mechanisms up to this phase of the work. Our observation could be due to the fact that, very often and especially in older works, a fit considering only the Orbach process was applied to the relaxation times obtained at the highest temperatures. This was done even in systems where at very low temperatures the magnetic behaviour points towards a purely quantum tunnelling or Raman relaxation mechanism. To get further insights on this problem, we explored the available data on the two latter processes in the next section.

5.5 Dependence of τ_0 *vs* U_{eff}

Let us address in more detail the remaining question of whether the effective energy barrier, despite being oversimplified, is meaningful.

In the classical text by Abragam and Bleaney (Published 1970, reprinted 2012, chapter 10, page 561, eq 10.55)[62] offered the following relation for the two-phonon Orbach process, assuming a Debye model for phonons:

$$\frac{1}{\tau_1} = \frac{3}{2\pi\hbar^4\rho\upsilon^5}\left|V^{(1)}\right|^2 U_{eff}^3 \frac{1}{exp\,(U_{eff}/kT)-1} \quad (1)$$

In terms of notation, note that in the book Δ is employed for the energy difference between the starting state |b> and the excited state |c> in the two-phonon Orbach process, before relaxation to the final state |a>. Thus, U_{eff} in our dataset, and in equations (1) and (2), corresponds to Δ in the book.

Two major objections to the validity of this approximation are, (a) there is a consensus that the vast majority of studied compounds present relaxation mechanisms dominated by multiphonon processes i.e. high-order Orbach processes involving successive excitations to higher states followed by a cascade of de-excitations and (b) the Debye model is nowadays known to be a bad match for the local vibrations responsible for relaxation in molecular nanomagnets. Arguably, for our purposes this is still an interesting representation. Despite the first objection, one needs to consider that the two-phonon Orbach process with a single excited state corresponds well with the Arrhenius equation that has been widely employed in the literature to extract parameters τ_0 and U_{eff}. About the second objection, one will just need to remember that part of the deviations of experimental data from this equation that one will find will be precisely due to the failing of the Debye model in magnetic molecules. For more on the repercussions on the failing of the Debye model, see the discussion in Supplementary Section 9.

From (1) we establish a relationship with the Arrhenius equation in the limit $U_{eff} >> kT$ (which is always the case in the experimental data, since at temperatures of the order of U_{eff} there is no slow relaxation of the magnetization):

$$\frac{1}{\tau_1} = \frac{1}{\tau_0} \cdot exp\,(-\,U_{eff}/kT) \quad (2)$$

From combining (1) and (2) we extract the approximate relation:

$$\frac{1}{\tau_0} = R_{Or} \cdot (U_{eff})^3 \quad (3)$$

, where we introduced an Orbach relaxation rate R_{Or} as:

$$R_{Or} = \frac{3}{2\pi\hbar^4\rho\upsilon^5}\left|V^{(1)}\right|^2 \quad (4)$$

We can rewrite Eq. (3) above more generally as:

$$\frac{1}{\tau_0} = R_{Or} \cdot (U_{eff})^n \qquad (5)$$

where n = 3. According to Abragam and Bleaney, reasonable parameters for rare earth elements resulted in an Orbach rate $R_{Or} \approx 10^4$ $K^{-3} \cdot s^{-1}$, and early experimental results were in the range 10^3 $K^{-3} \cdot s^{-1} < R_{Or} < 10^5$ $K^{-3} \cdot s^{-1}$.

Plotting the data available in the dataset in terms of τ_0 *vs* U_{eff} allows one to quantify the deviations, in practice, from the assumptions in eq. 10.55 in Abragam and Bleaney, as commented above. The results from the fits can be found in Supplementary Table 5.

Supplementary Table 5 | Least squares fits of ln(U_{eff}) *vs* -ln(τ_0) and ln($U_{eff,ff}$) *vs* -ln($\tau_{0,ff}$).

Data	Intercept (R_{Or})	Slope (n)
All	839.4	2.437
Prolate	2557.5	2.454
Oblate	504.3	2.506
Tb^{3+}	700.5	2.415
Dy^{3+}	504.0	2.515
Full fit	151.2	2.957

Note that the slopes are identical but the constant term is higher for oblate ions compared with prolate ions. We find R_{Or}(prolate) ≈ 5·R_{Or}(oblate), meaning that, for comparable U_{eff}, τ_0 for oblate ions is on average substantially greater, and relaxation substantially slower, than that for prolate ions (Supplementary Fig. 24.1). This is consistent with the observation that complexes of oblate ions present values of T_{B3} higher than expected considering their U_{eff} (see Supplementary Fig. 11.3). meaning an equivalent U_{eff} relaxation will be substantially slower in oblate ions). Within the two main oblate ions (Dy^{3+} and Tb^{3+}), the slope is slightly higher for Dy^{3+}, meaning a dramatic increase in U_{eff} is somewhat more beneficial for Tb^{3+} compared with Dy^{3+} (Supplementary Fig. 24.2).

The limited (<100) data points of $U_{eff,ff}$, $\tau_{0,ff}$ pairs, where all relaxation processes were considered, present a better agreement on the exponent, with $n \approx 3$ but lower Orbach rates R_{Or} ≈ 150 $K^{-3}s^{-1}$. For the rest of the dataset, given the limitations pointed out above, the coincidence with the expectation from the relationship in Eq. 5 is reasonable. The minor discrepancy with the expected exponent, a value of n that is between 2.4 and 3 instead of n=3, and values of R_{Or} of the order of 10^3, around or below the expected lower limit of the range 10^3-10^4, serves as an independent evaluation of the limitations of a two-phonon Orbach model that also assumes a Debye model for phonons.

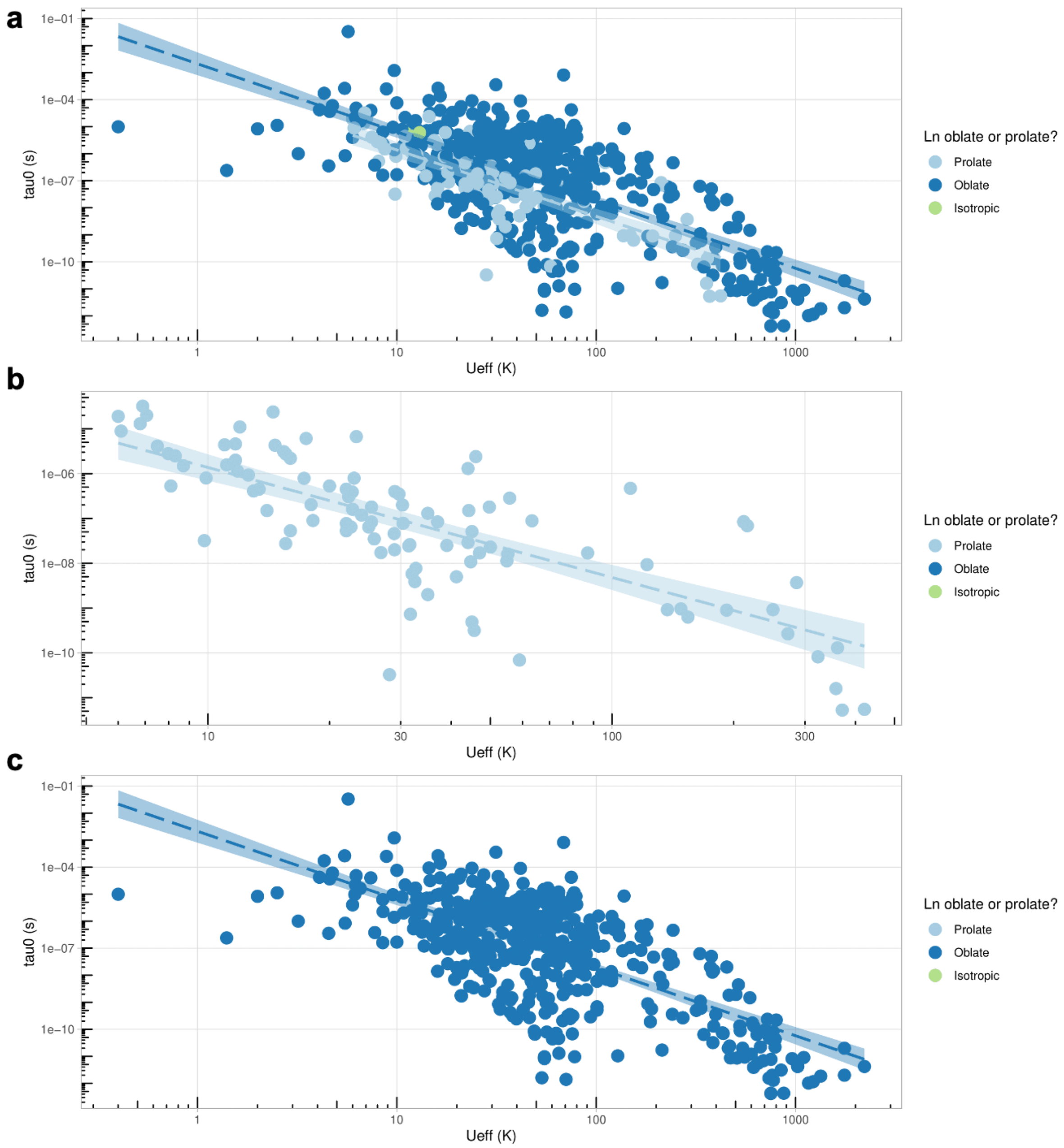

Supplementary Figure 24.1 τ_0 *vs* U_{eff}, for prolate and oblate ions. a, Comparison between both. **b,** Only prolate ions. **c,** Only oblate ions. Note that not all samples will be present in all graphs (see Supplementary Figure 1.1). As a consequence, an (x vs y) plot can only include samples for which x and y are simultaneously present in the dataset.

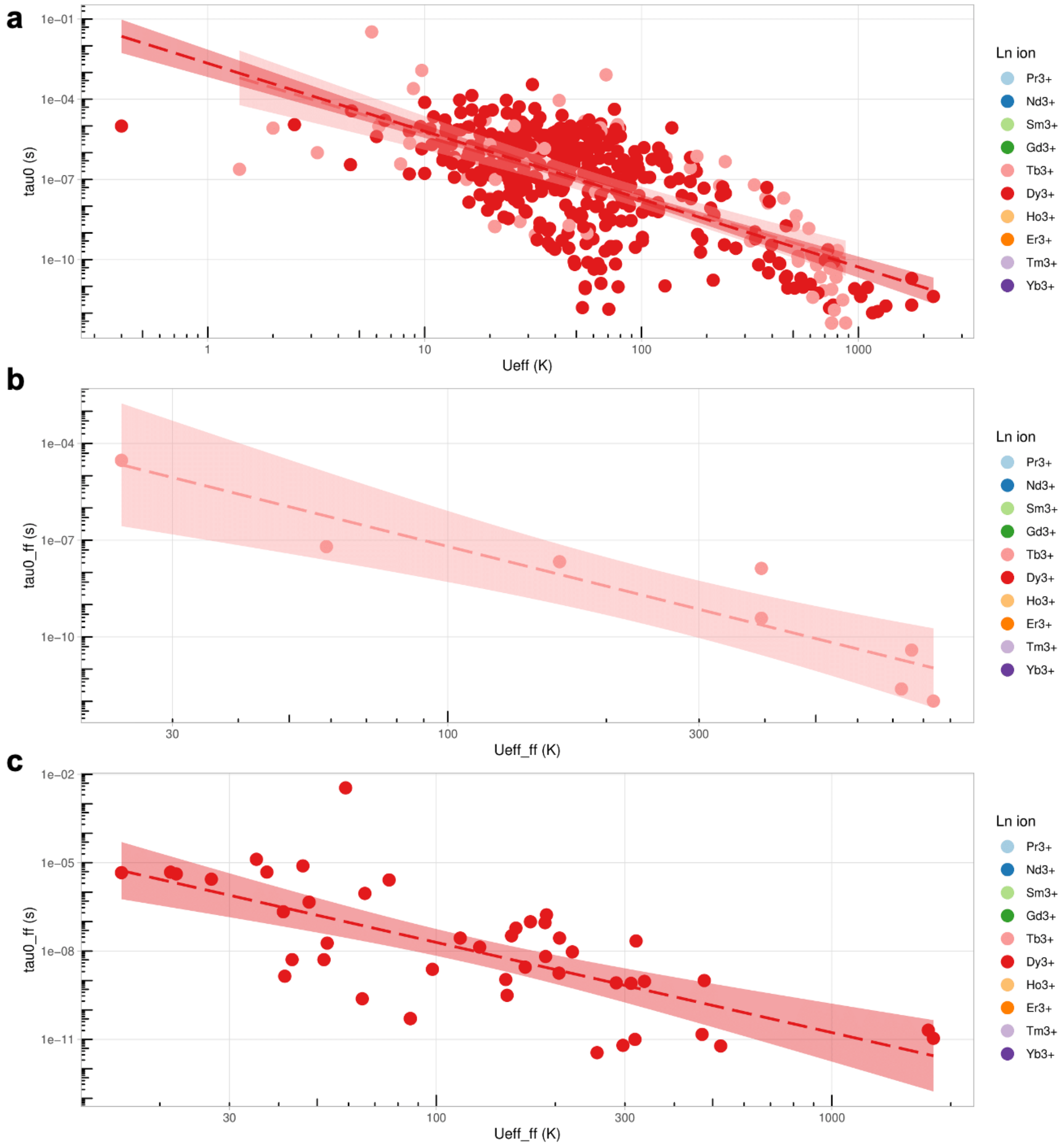

Supplementary Figure 24.2 | τ_0 *vs* U_{eff} and $\tau_{0,ff}$ *vs* $U_{eff,ff}$ for Tb^{3+} and Dy^{3+}. a, Comparison between τ_0 vs U_{eff} for both ions. **b,** $\tau_{0,ff}$ vs $U_{eff,ff}$, only Tb^{3+} complexes. **c,** $\tau_{0,ff}$ vs $U_{eff,ff}$, only Dy^{3+} complexes. Note that not all samples will be present in all graphs (see Supplementary Figure 1.1). As a consequence, an (x vs y) plot can only include samples for which x and y are simultaneously present in the dataset.

Supplementary Section 6. FAMD and magnetostructural clustering

For all the statistical studies of the physical variables that follow in the present section, we performed the analysis two times, to check for consistency and robustness of the results. In particular, we performed the analysis of the full dataset (~1400 samples) and repeated it independently employing only the data subset in the timeframe 2003-2017 (~1000 samples). We found that all major qualitative results presented here are robust and independently obtained whether one considers the whole set 2003-2019 or the 2003-2017 subset. Furthermore, quantitative data were found to be within a 25% deviation, with a shift towards higher values of U_{eff}, T_{B3} and T_{hyst} in the 2003-2019 set when compared with the 2003-2017 subset.

Factorial analysis of mixed data (FAMD) is a factorial method appropriate to analyse data containing both quantitative and qualitative variables. FAMD is a versatile method that acts as Principal Component Analysis for quantitative variables, and as Multiple Correspondence Analysis for qualitative variables. Qualitative and quantitative variables are normalised during the analysis to equilibrate the influence of each in the variable set. In this case this allows us a simultaneous analysis of physical and chemical properties, to perform a hierarchical clustering of samples with the goal of producing a magnetostructural taxonomy in our Ln-based SIMs catalogue. By grouping the samples by taking into consideration their molecular structure and their magnetic behaviour, we can aspire to obtain information on the main relation between form and function. To perform this analysis and data representation we employed R packages FactoMineR[57] and factoextra.[63]

We found that the chemical family, the lanthanide ion and the coordination elements are the best chemical predictors, as U_{eff} among the physical parameters. Only 608 samples in the dataset contain quantitative U_{eff} and τ_0 data. We initially worked just with these 608 samples, and later repeated the analysis with all samples, obtaining the same result.

Let us start with representing the relation between the two main FAMD dimensions and the main physical and chemical variables (Supplementary Figs. 25, 26 and 27). Analysing the contribution from the main chemical and physical variables to the main FAMD dimensions (Supplementary Figs. 25 and 26), one can see that the distinctive traits for both dimension 1 and dimension 2 are the variables “chemical family” and “coordination elements”, with the “Ln ion” choice appears in a distant third place, while “U_{eff}” participates only in dimension 1. This is similar to what was seen in Supplementary Section 4.2. The FAMD factor map (Supplementary Fig. 27) provides additional information on the actual values of the variables presented by the samples and their relation to the two main dimensions.

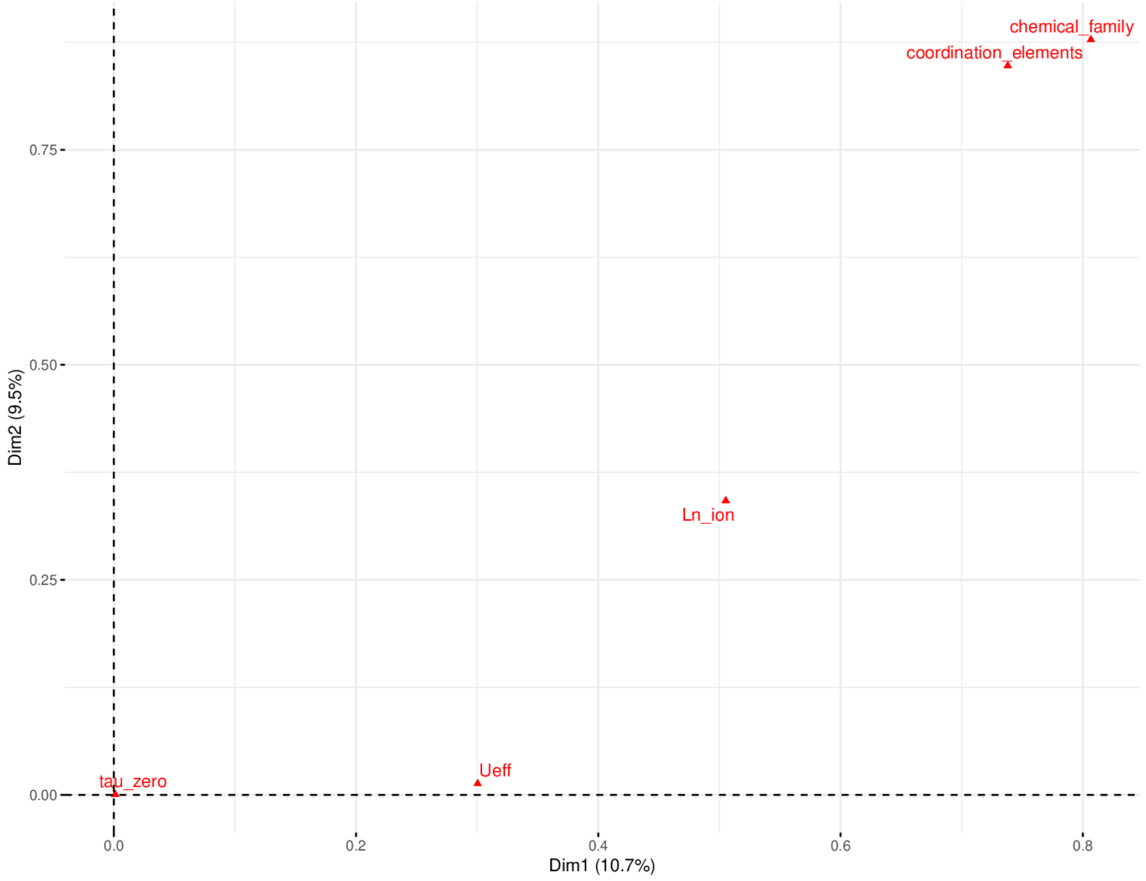

Supplementary Figure 25 | Representation of the physical and chemical variables according to a FAMD method.

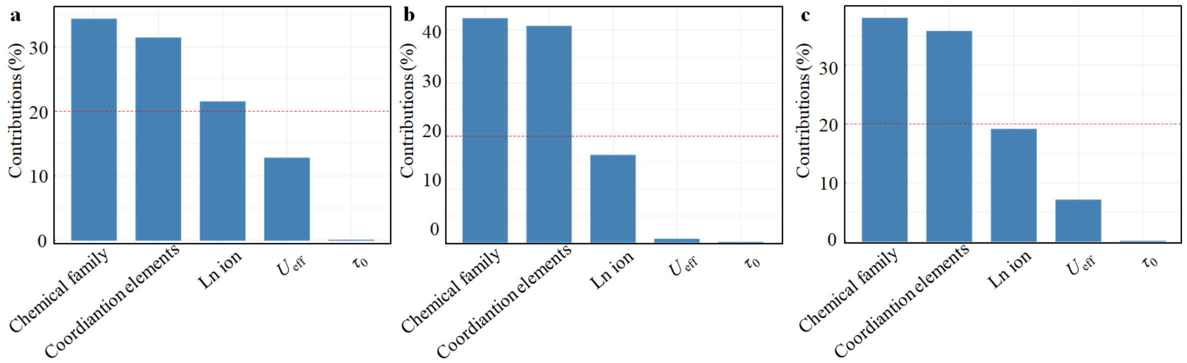

Supplementary Figure 26 | Contribution from the main chemical and physical variables to the main FAMD dimensions. a, Contributions to dimension 1. **b**, Contributions to dimension 2. **c**, Combined contributions to dimensions 1 and 2.

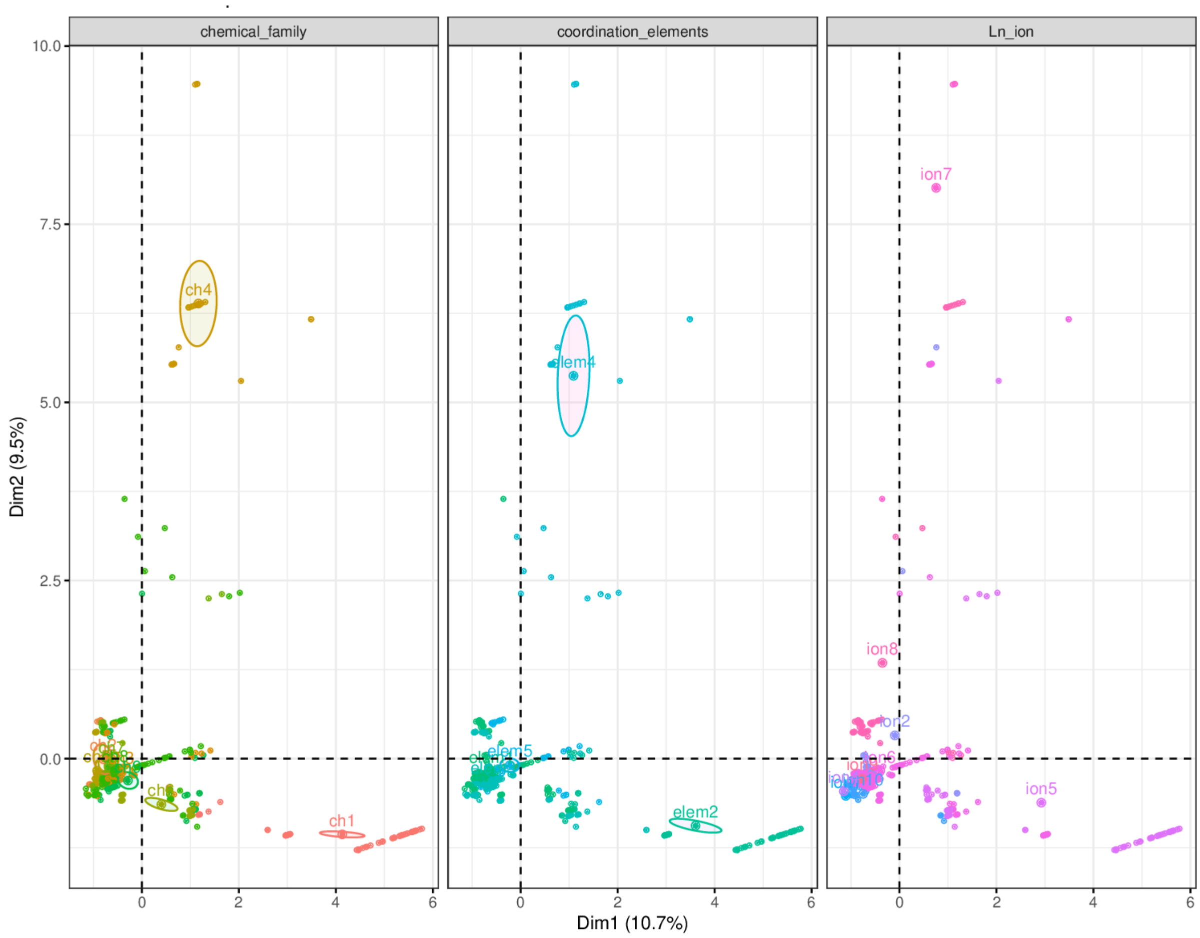

Supplementary Figure 27 | FAMD factor map. The groupings of the different values for the three main chemical variables is shown. See numbering convention for the categories of variables in Supplementary Section 1.

6.1. Magnetostructural clusters

We proceed to analyse the magnetostructural hierarchical clustering. This is comparable with the molecular clustering presented in Supplementary Section 4.2, but considering both physical and chemical variables. Dendrograms are represented in Supplementary Figs. 28 and 29, and a description of the different clusters follows.

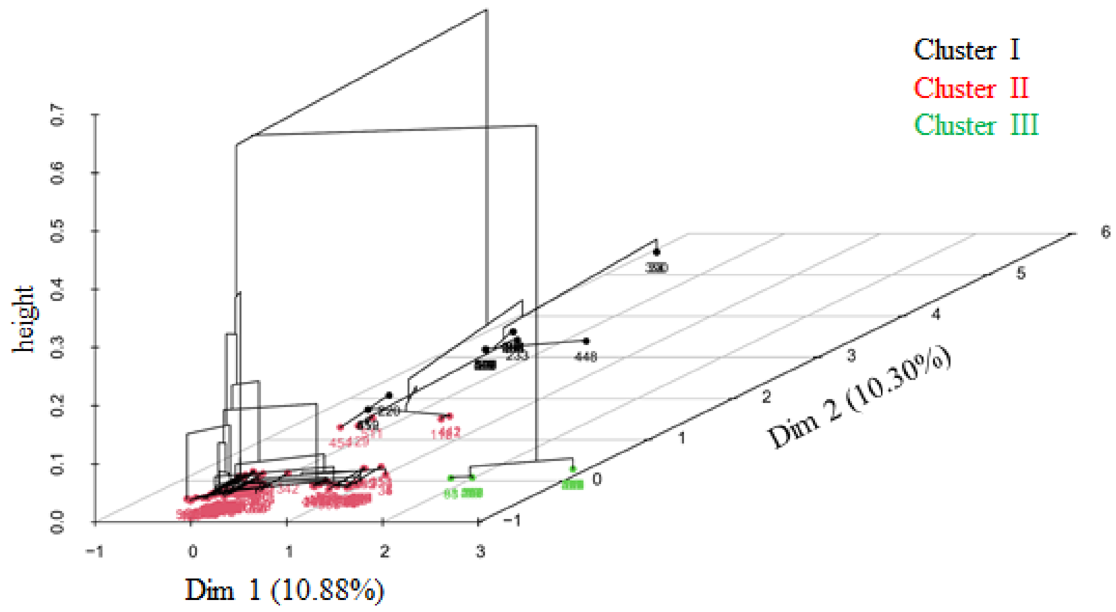

Supplementary Figure 28 | Dendrogram depicting the hierarchical clustering on the factor map. The numbers in the plot represent sample_IDs.

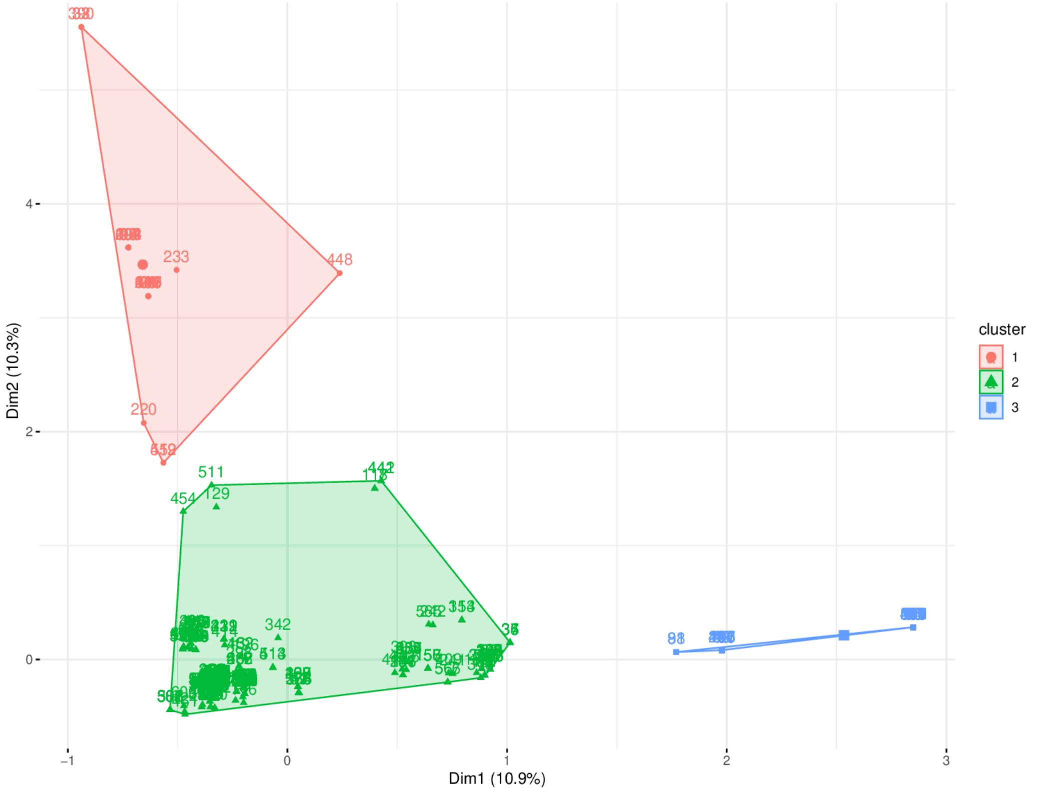

Supplementary Figure 29 | Alternate view of the calculated hierarchical clustering on the factor map. The numbers in the plot represent sample_IDs.

The main results from these analysis are as follows:

-Cluster I: U_{eff} is the most associated variable in cluster I. The average value of U_{eff} (252 K) in cluster I is considerably higher than the general average of U_{eff} (117 K). In addition, the

average value of T_{hyst} in cluster I (9.9 K) is also significantly above the average value of T_{hyst} (5.5 K). Indeed, cluster I is characterised by higher-than-average values of U_{eff} and T_{hyst}.
-Cluster II is characterised by close-to-average values of T_{B3} (18.5 K < 19.2 K) and T_{hyst} (5.2 K < 5.5 K), and lower-than average U_{eff} (87 K < 117 K).
-Cluster III, like cluster I but less intensely, is characterised by higher-than-average values for U_{eff} (353 K > 117 K) and T_{B3} (26 K > 19 K).

A partial clustering taking into account of the first 1000 data points (*i.e.* discarding data from 2018 and 2019) results in a very similar classification, but primarily characterises cluster I by higher-than-average values for U_{eff} and T_{B3} and cluster III by higher-than-average values for T_{hyst} and U_{eff}. As a notable difference, discarding recent data results in a significant decrease in the average value for cluster III down to U_{eff} = 199 K.

This general "magnetostructural" clustering classification, when described strictly from the point of view of the chemical variables, is depicted in Supplementary Fig. 30 and can be simplified to:
·cluster I: metallocene-type sandwiches, carbon as donor atoms, with Ho^{3+} and Er^{3+} as the most abundant ions.
·cluster II: predominantly mixed ligands, *i.e.* a mixture of different coordination ligands, predominantly Dy^{3+} ion, and either only oxygens or a mixture of nitrogen and oxygen as donor atoms. This is by far the most abundant class of compounds with reported U_{eff}.
·cluster III: Tb^{3+} ion (followed by Dy^{3+}), $LnPc_2$ family, nitrogens as donor atoms. These values for these variables are partially overlapping.

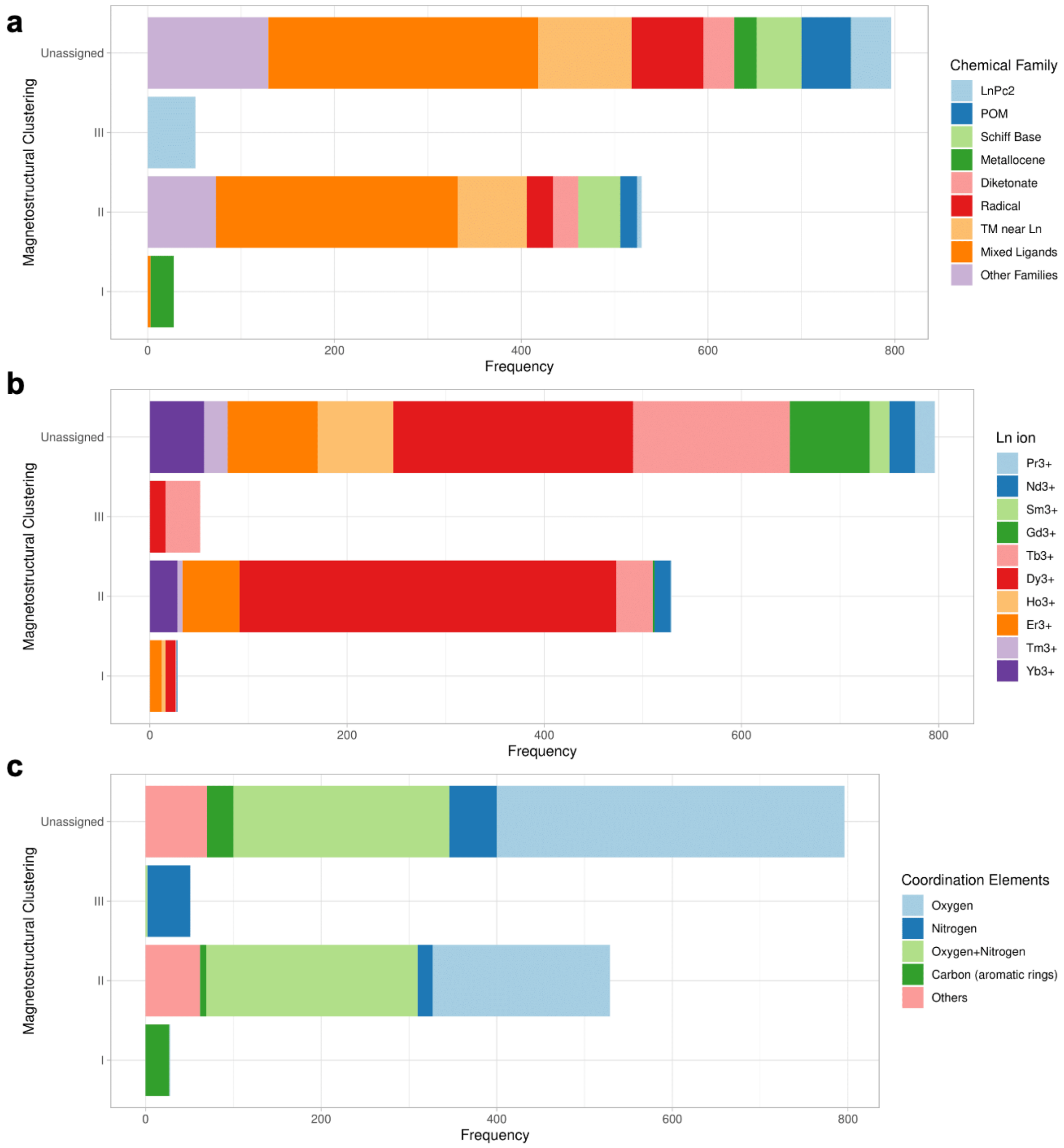

Supplementary Figure 30 | Bar charts for magnetostructural clusters and their relation with the main chemical variables. From top to bottom, the bar charts are filled according to: **a,** chemical family; **b,** lanthanide ion and **c,** coordination elements in the coordination sphere.

The correlation between the magnetostructural clusters I-II-III and the chemical clusters A-B-C-D-E is depicted in Supplementary Fig. 31, and can be summarised as follows; for readability we add the main feature of each cluster in parentheses:

-cluster I is mostly composed of samples from cluster E (metallocene)

-cluster II is a mixture of samples from cluster B (oblate) and C (prolate), and as well some from cluster D, but mostly from B

-cluster III is mainly samples of cluster D ($LnPc_2$)

-samples outside the I-II-III classification (not assigned), meaning samples with no recorded value of U_{eff} are a mixture of all of the A-B-C-D-E cases, notably including all of the cases of cluster A (Gd^{3+} complexes), and are, in order of relative abundance: B, C, A, D, E.

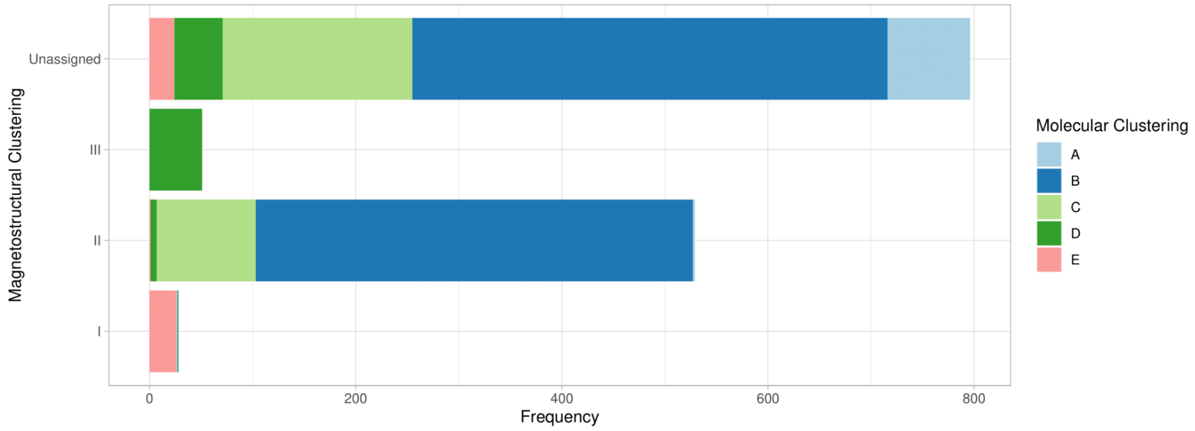

Supplementary Figure 31 | Bar charts for magnetostructural clusters and their relation with the molecular (chemical) clusters. NA stands for not assigned samples, *i.e.* samples that do not belong in any of the 3 magnetostructural clusters I-III.

As in the molecular clustering, depending on the height of the dendrogram cut in Supplementary Figure 28, one can obtain more or less fine-grained clusters. Our dataset contains a main categorisation with 3 tipologies I-III (+ “Unassigned”), described below, and we always offer an alternate, finer categorisation within the same hierarchical clustering (mag_struct_cluster_2 in the dataset) with 8 tipologies I-VIII (+ “Unassigned”), which is also included in the Data tab of the the SIMDAVIS dashboard.

Supplementary Section 7. Extended SHAPE analysis, comparison with reference polyhedra

7.1 Methodology

In order to define the ligand environment to analyse the reference polyhedra, we used PyCrystalField[64] to extract the nearest neighbour ligands to define the reference polyhedra from the material crystallographic information files.[65] PyCrystalField is a software designed to calculate the ligand and crystal field Hamiltonian of a single ion using a point charge model. Prior to this study, PyCrystalField considered only the space-group symmetry of the crystal, which is generally enough for conventional solid-state materials. Due to the diverse and low-symmetry environments that are generally found in molecules, and thus in the SIMDAVIS dataset, we added the ability to identify near-symmetries using continuous symmetry measures.[66] In the present work, this allows analysis of the correlations between the shape of the coordination environment and other variables. In future works, it will also allow intelligent predictions of the single-ion states and quantization axes of low-symmetry crystals and molecules (including single-ion magnets).

Equipped with this new functionality, we wrote a script to batch-process the ligand environments of all materials in the SIMDAVIS database, using the following routine. In cases where the coordination number CN was identified in the original study, we took the n=CN nearest atoms to the central magnetic ion as the coordination sphere. However, in some cases CN was not identified in the literature, for a variety of reasons. In these cases we used the following automated procedure: starting with n=7, we took the nearest n atoms to be the coordination sphere, and calculated their geometrical centroid. If the central magnetic ion was off from the centroid by more than 25% of the greatest bond length, we added another ligand (or group of ligands if the next nearest ligands are all the same ion) and recalculated. This procedure was followed up to n=20. After identifying the appropriate ligand sphere, we used the SHAPE software[67] to identify the closest reference polyhedra, as well as the continuous shape measure (CSM) to it, and added both to the dataset. Note that in the specific case of aromatic ligands, the formal coordination number CN in our dataset may be much less than the number of atomic ligands n because aromatic rings are counted as a single coordination ligand. The script for batch-processing can be found at:

https://github.com/asche1/PyCrystalField/tree/master/Publications/SIM_BatchProcess

Although we do not report the results here, PyCrystalField allows us to estimate the single-ion ground state wavefunction of each material using the point charge model. There are significant challenges in appropriately assigning effective charges to the ligands, and in orienting the coordination sphere in extreme low-symmetry cases. Because solving these issues is an ongoing task, we leave the wavefunction calculations for a future iteration of this project.

7.2 Results

Let us analyse the most frequent coordination polyhedra, and in particular the polyhedra that are most frequent for the most common coordination numbers, namely CN = 7-9 (see Supplementary Figure 32.1). And in this analysis, let us answer the question: are the different coordination polyhedra equally good, in terms of frequently resulting in magnetic memory effects at relatively high temperatures? The results, which we shall discuss below, are in Supplementary Figure 32.2.

First note that, since the data are relatively scarce compared with analyses of previous sections, we were merely looking for differences that can be striking when the results of a polyhedron type are compared with the results of the whole dataset. Moreover, it is important to allow the reader to keep in mind the scarcity of this kind of information, and the possibility of the insights to be overly influenced by a few outlier compounds. For this purpose, when the number of samples < 50 for a given type of polyhedron, in the discussion we include in parentheses the total samples as "ts", and the number of unique doi (articles) associated with this polyhedron as "ud".

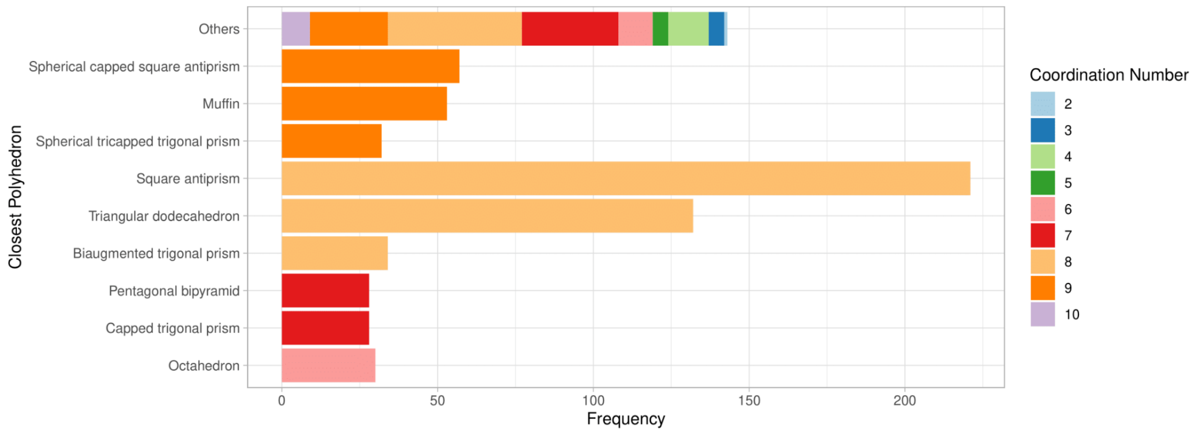

Supplementary Figure 32.1 | Bar charts for closest polyhedra and their relation with the coordination number. One can see the most abundant values for CN, and the most abundant polyhedra.

Let us start with CN=7, where, as we shall see, the sharpest contrast can be found between the different coordination shapes. The most common polyhedra with CN=7 are pentagonal bipyramids and capped trigonal prisms. Capped trigonal prisms (28 ts, 13 ud) tend to present no frequency dependent x'', much less an ac peak or hysteresis. In this sense, capped trigonal prisms presents among the worst magnetic results for any shape in the present dataset, at least when it comes to direct experimental results (its distribution of U_{eff}, τ_0 values, while following this tendency, i.e. slightly lower U_{eff}, higher τ_0, are not markedly different from the rest of the dataset). In contrast with the capped trigonal prism shape, pentagonal bipyramid (29 ts, 18 ud) is apparently the shape with the highest tendency to present an ac peak at T > 2K and f = 1000 Hz and to present hysteresis (see Supplementary Figure 32.2).

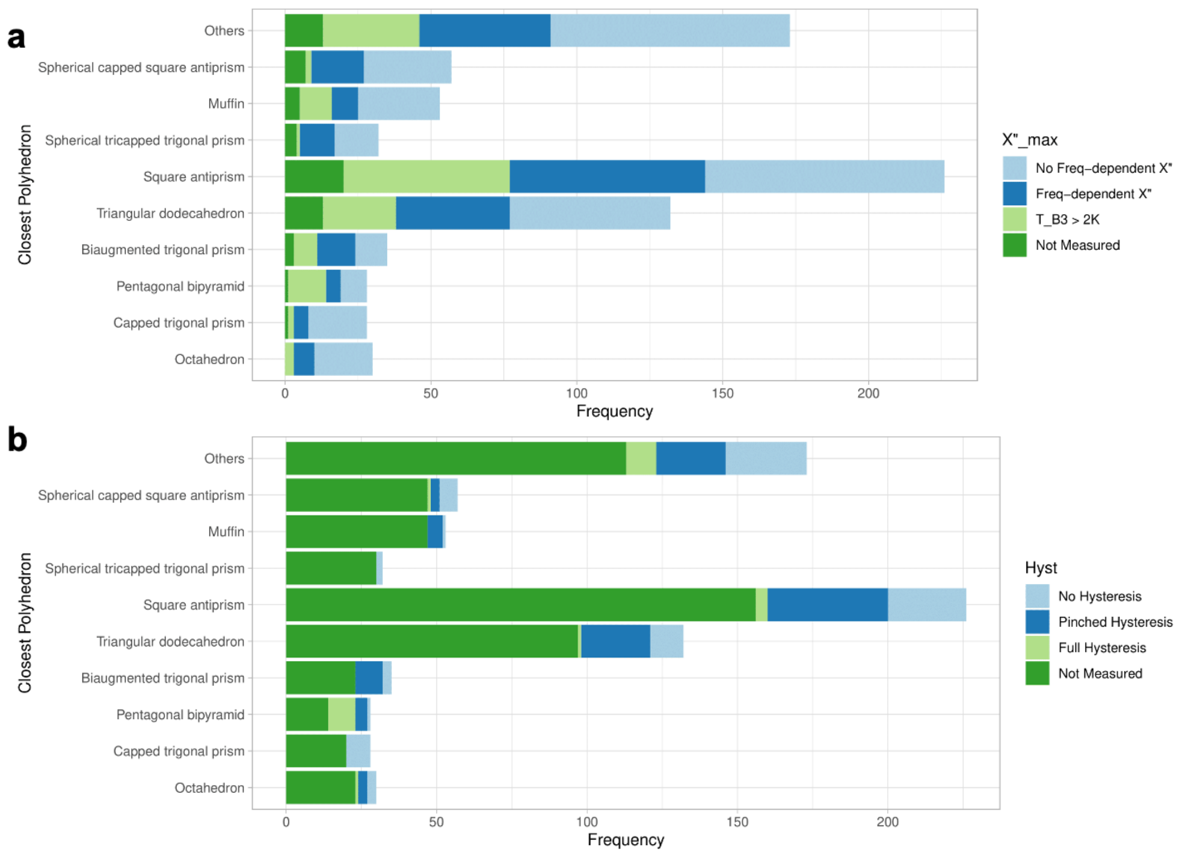

Supplementary Figure 32.2 | Bar charts for closest polyhedra and their relation with the magnetic behaviour. a, out of phase ac susceptibility response and **b,** hysteresis.

Moreover, said ac peak tends to be at very high temperatures compared with other shapes (median T_{B3} > 30 K), with the same observation being true for T_{Hys} (median T_{Hys} > 10 K, when most other shapes present median T_{Hys} in the window 2K< median T_{Hys} < 4K). Both of these observations can be quantified in Supplementary Figure 32.3. As can be seen in Figure 6, in terms of parameterized Arrhenius behaviour, pentagonal bipyramids in our dataset present outstanding values of U_{eff}, with a median value U_{eff} median ~ 400 K that is an order of magnitude above the usual for other coordination shapes (U_{eff} median ~ 40 K). τ_0, as we see elsewhere in the present analysis, is strongly correlated with U_{eff}, and in this case pentagonal bipyramids tend to present values of tau0 that are much below the usual range for other polyhedra (median $\tau_0 \sim 10^{-11}$ s, when most other shapes present median 10^{-8} s < $\tau_0 < 10^{-7}$ s).

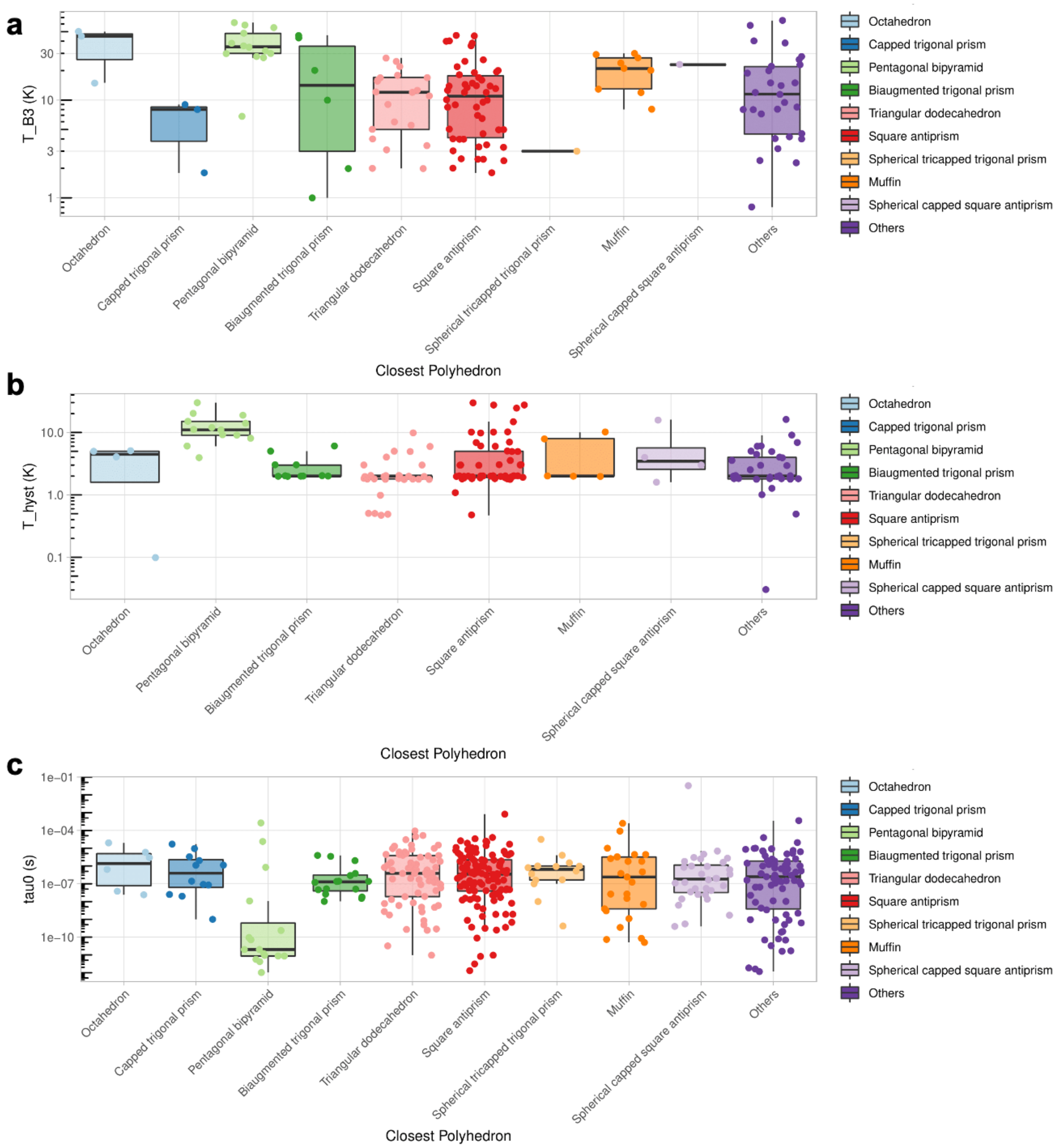

Supplementary Figure 32.3 | Box plots for closest polyhedra and their relation with the magnetic behaviour. From top to bottom: T_{B3}, T_{hyst}, τ_0.

Let us investigate this unusual behaviour presented by the pentagonal bipyramid (PBPY-7) and try to rationalise it with the help of the spherical harmonics (see Supplementary Figure 32.4). First one needs to note that a regular PBPY-7 polyhedron, much like a regular square antiprism, presents only diagonal components in the crystal field Hamiltonian, with all other contributions being zero, either because the ligands are located on a node or due to symmetry cancellation. This has been very often argued to be a positive feature, since it facilitates pure crystal field states, minimising the mixing and thus lowering the transition probabilities between states. However, this cannot be the only reason behind the success of PBPY-7 complexes, since we have seen that square antiprisms are not as markedly good as SIMs. We

need to note, additionally, that a PBPY-7 presents only two types of ligands: perfectly axial ligands and perfectly equatorial ligands. In both cases, this means that the position of each donating atom coincides with an angular maximum of the diagonal spherical harmonics, for B_2^0, B_4^0 and B_6^0. In this context, let us consider vibronic coupling. Vibrations that alter metal-ligand bond distance tend to be high-energy and thus, in practice, are not the ones limiting the working temperature for slow relaxation of the magnetisation. In contrast, vibrations where metal-ligand bond distance is kept constant and only the angles change can be low frequency. It is against these distortions where being exactly at an angular maximum is important, since it means that the first derivative of the spherical harmonics, and thus, the change in any diagonal term in the crystal field Hamiltonian, is zero. We postulate that this special geometrical correspondence allows pentagonal bipyramids to be specially resilient to angular (twisting, wagging, bending) vibrations which, being the lowest in energy, can be present even at low temperatures and facilitate magnetic relaxation most often.

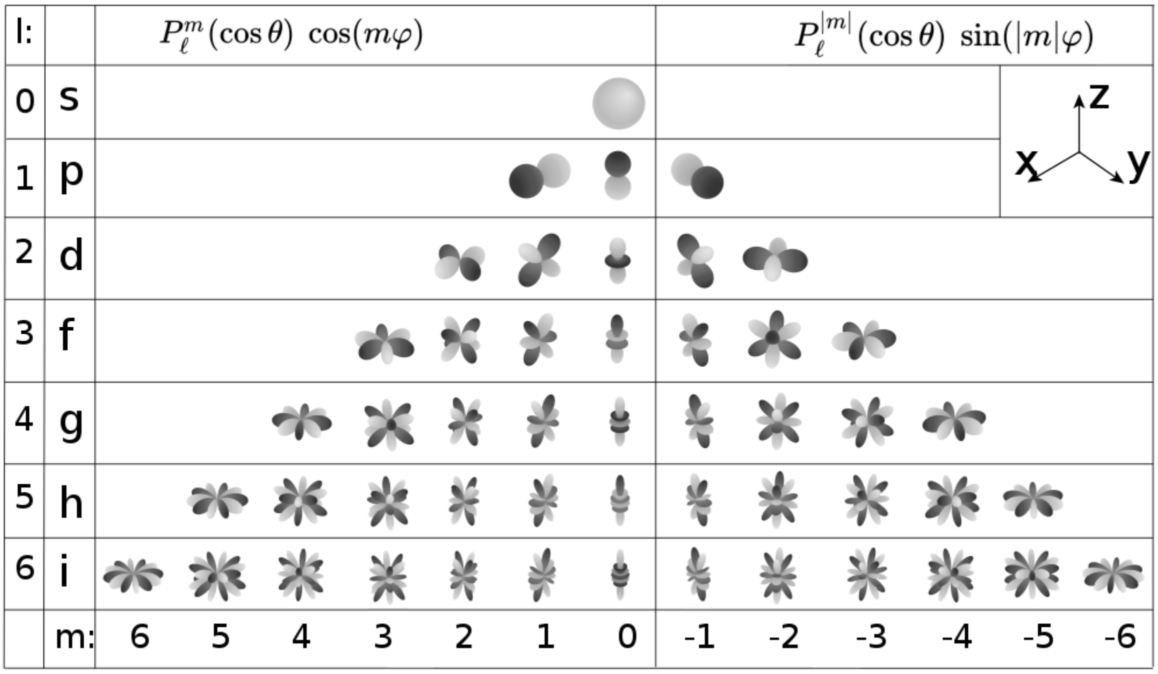

Supplementary Figure 32.4 | Spherical harmonics. Only the shapes corresponding to l = 2,4,6 are relevant for Crystal Field in lanthanides.

In the case of CN=8, the most common polyhedra are square antiprisms, triangular dodecahedra and biaugmented trigonal prisms (35 ts, 21 ud), which not only come in a far third place but also consistently present higher distortions CSM. Between square antiprisms and triangular dodecahedra in our dataset, there is no marked difference neither in the magnetic performance nor in the parameterization. This is notable, since proximity to square antiprisms has historically very often been invoked as a promising geometry to obtain SMM behavior. Biaugmented trigonal prisms tend to present higher values of U_{eff} and lower values of τ_0 compared with other polyhedra, but there is no marked difference in their magnetic performance.

Finally, in the case of CN=9, the most common polyhedra are spherically capped square antiprisms, muffins and spherically tricapped trigonal prisms (36 ts, 20 ud). The differences between the three shapes are more marked than in the case of CN=8 but less than in the case of CN=7. Among CN=9, muffin polyhedra, which present a high degree of distortion compared with any other polyhedra in our dataset, present an ac peak at T > 2K at f = 1000 Hz and also present hysteresis most often, and in both regards spherically capped square antiprisms come in second place and spherically tricapped trigonal prisms comes last. In terms of maintaining magnetic memory up to high temperatures, CN=9 muffins are not as exceptional as the CN=7 pentagonal bipyramids we discussed above, but they do perform markedly better than any CN=8 shape, again, shockingly, given the popularity of square antiprisms.

Also deserving further theoretical study (in the next section) is the fact that U_{eff}, T_{B3}, T_{Hys} vs CSM present overall positive slopes, meaning that higher distortion tends to produce higher U_{eff}, T_{B3}, T_{Hys}. This can be initially surprising, if one is thinking in terms of extradiagonal parameters in the crystal field Hamiltonian. It has often been argued precisely that ideal geometries are preferable to avoid mixing in the spin states. However, it makes more sense if one considers that reference polyhedra in SHAPE are as spherical as possible, meaning B_2^0 tends to cancel.

Supplementary Section 8. Extended SHAPE analysis, axial distortions

8.1 Methodology

For the analysis of the effect of structural distortions, we aimed to quantify the elongation or contraction of each coordination sphere employing SHAPE. Note that the reference polyhedra in SHAPE are as spherical as possible, in the sense that all vertices are at the same distance from the centre. So, after determining the overall distortion as CSM for the coordination sphere of each complex, our goal in this step was to get some insight into the character of said distortion: is it systematically elongated, or contracted, or are the distortions of a rather "isotropic" character? This is however not a standard feature of the program, so we developed an auxiliary methodology, which we explain here.

We first obtained a set of reference structures for the 9 most represented families of coordination polyhedra within our dataset (see Supplementary Table 6): capped square antiprism (CSAPR-9), square antiprism (SAPR-8), triangular dodecahedron (TDD-8), pentagonal bipyramid (PBPY-7), tricapped trigonal prism (TCTPR-9), muffin (MFF-9), biaugmented trigonal prism (BTPR-8), octahedron (OC-6) and capped trigonal prism (CTPR-7). The coordinates for the reference polyhedra were obtained from the SHAPE program by employing the keyword %test . We fixed the resulting orientation, which puts the z axis as a maximum symmetry axis. This z axis was the one we employed as a reference for axial compression or axial elongation. Note that in principle all results in the present analysis are conditioned by the choice of the elongation/compression axis.

Supplementary Table 6: Frequencies of the 9 most abundant closest polyhedra within the data set.

#	Closest polyhedron	Counts
1	Square antiprism	226
2	Triangular dodecahedron	132
3	Spherical capped square antiprism	58
4	Muffin	54
5	Spherical tricapped trigonal prism	36
6	Biaugmented trigonal prism	35
7	Octahedron	31
8	Pentagonal bipyramid	29
9	Capped trigonal prism	28

Path to repository with the sampling script:

https://github.com/silsgs/extended_SHAPE_analysis/blob/main/simpre_sampling_sph.sh

Example of SHAPE script generating an ideal 6-vertices octahedron coordination structure ("OC-6").

```
$ Generate X-vertices reference eg: 6-vertices refs OC-6; 7th atom is the metal

%test

  6 7

  3
```

Output file:

```
  7

H         0.00000000   0.00000000  -1.00000000
H         1.00000000   0.00000000   0.00000000
H         0.00000000   1.00000000   0.00000000
H        -1.00000000   0.00000000   0.00000000
H         0.00000000  -1.00000000   0.00000000
H         0.00000000   0.00000000   1.00000000
N         0.00000000   0.00000000   0.00000000
```

In a second step, and starting from the ideal polyhedron structure of each family (obtained from SHAPE), we propose to standardise a methodology to quantify non-regular structures assigning a value representative of their 'elongated' or 'compressed' character versus the ideal structure. For that, ideal structures were either axially compressed or elongated up to 10%, 20% and 50%. In practice, an extension/compression factor between 0.5-1.5 over the z coordinate of each ideal polyhedron was applied. For each of the 8 new distorted structures (varying z' from z'=z·0.5 to z'=z·1.5) for each reference polyhedron in Supplementary Table 6, we prepared a file of user-defined reference polyhedra. We run SHAPE in batches employing the code 0 to command SHAPE to read a user-defined "ideal" (.ide) reference file. Following this procedure, we obtained, first, a series of CSM(z') values, measuring how much each coordination polyhedron differs, not from the reference "spherical" polyhedra, but from each of the elongated or compressed references. In turn, this allowed us to obtain a series of new axial distortion quantifications Δ by difference of continuous shape measures CSM:

axial distortion $\Delta_{CSM(z')}$ = CSM(z')-CSM(original)

Let us briefly discuss what this axial distortion $\Delta_{CSM(z')}$ means in practice. If (for a given coordination sphere, reference polyhedron and axial distortion) $\Delta_{CSM(z')}$ is negative it means that this coordination sphere is closer to an axially distorted reference (smaller value of CSM(z')) rather than to the original one (larger value of CSM(original)). For example, let us say we are examining a hypothetical square antiprism (SAPR) where the CSM with respect to

the reference SAPR is CSM(z·1) = 3. The question we want to answer is: is this strong distortion mostly axial, mostly isotropic, or somewhere in between? An example of a mostly axial distortion case would be an elongated SAPR which, other than that, is a rather perfect D4d. An example of a mostly isotropic distortion would be a distorted SAPR where all spatial coordinates of the coordinating atoms have suffered random changes. An intermediate example, of course, would be a mixture of random distortions and axially directed ones.

A way to distinguish between these three situations is calculating $\Delta_{CSM(z')}$ for different values of z'. We can see this as a sampling of axial distortions. The values of $\Delta_{CSM(z')}$, plotted against z', look like a local minimum. Thus, the value of z' where one finds the most negative value of $\Delta_{CSM(z')}$, i.e. the minimum value of CSM, corresponds to the reference polyhedron with the elongation/compression that most closely resembles the one of the real coordination sphere. So with this set of calculations we would have an (admittedly rough) categorisation corresponding to this sampling of $(z'/z) \in \{0.5,0.75,0.9,1,1.1,1.25,1.5\}$.

However, our goal is slightly more ambitious, so additionally we prepared a minimally elongated structure (elongated by 1% i.e. multiplying z·1.01) to establish a distortion slope, which is a description of how much is CSM varying when certain distortion is applied, and it is defined as

axial distortion CSM slope s = 100*(CSM(z·1.01)-CSM(original))

This axial distortion CSM slope served as an intermediate step towards obtaining our goal "axial distortion" continuous metric. We compared the $\Delta_{CSM(z')}$ values of all structures with their axial distortion CSM slopes, and found some pretty robust correspondence, not just qualitative but even quantitative. That is, not only the sign of the slope corresponds in every case to the sign in $\Delta_{CSM(z')}$, but also larger slopes consistently correspond to negative $\Delta_{CSM(z')}$ for higher values of z'. Let us see it with some examples. If $\Delta_{CSM(1.5)} \lesssim 0$, it would mean that the structure is extremely elongated, because the complex is a bit closer to a SAPR with z' = z·1.5 than to a "spherical" SAPR. One could assume that it's axial distortion is of the order of z' = z·1.25 (the elongation point where a structure starts being to a reference with z' = z·1.5 than to the original reference with z' = z). In these cases, we tend to find a threshold value of $s \approx -10$. Or, if $\Delta_{CSM(z')} > 0$ for all values of z', it means that the complex is not at all elongated or compressed, but rather distorted in another way. Thus, any elongation or compression to the reference polyhedron can only increase the CSM value. In these cases, we tend to find a threshold value of $|s| < 2$. Finally, if $\Delta_{CSM(0.9)} \lesssim 0$, it would means that this structure is at least slightly contracted. One could assume that its axial distortion corresponds approximately to z' = z·0.95 (the compression point where a structure starts being to a reference with z' = z·0.9 than to the original reference with z' = z). In these cases, we tend to find a threshold value of $s \approx 2.5$

We were able to extract the following approximate relation to estimate the axial distortion:

axial distortion = s^2 -2.5s +1

8.2 Results

In the Supplementary Fig. 33 a correlation of three magnetic parameters U_{eff}, $U_{eff,ff}$, T_{B3}, with the elongation / contraction of two most important polyhedra (PBPY-7 and SAPR-8) is presented.

In the top graph we observe that in the case of the pentagonal bipyramid, most of the data is restricted to a 'compressed' range of [0.92-0.94] with a dispersion of U_{eff} values from approximately 5K up to 900K. In contrast, for the case of the square antiprism, a high number of real structures are considered as 'elongated', with values around [1.00-1.20]. In all these cases, U_{eff} values still show some dispersion with a soft dependence showing a U_{eff} increase with the elongation of the structure. In both cases, a small but relevant correlation of the two parameters can be extracted being a positive slope for the case of the square antiprism and negative for the pentagonal bipyramid.

In the centre panel, the full-fit effective barrier $U_{eff,ff}$ is represented versus the elongation / compression of the square antiprism structure which is the only coordination geometry where there is sufficient $U_{eff,ff}$ data to show the dependence between these two values. In this case the number of points is very scarce because very few studies performed a full-fit study considering Direct, Orbach and Raman relaxation paths with a crystal structure solved. In concordance with that happening in the case of the Orbach-only fit, a weak positive correlation is found, with the barrier height increasing with the structural elongation.

Finally, regarding the bottom graph, the 10^3 Hz AC blocking temperature (T_{B3}) is shown versus the structural distortion. In this case, the pentagonal bipyramid shows the more relevant correlation with the structural compression/ elongation, again showing a negative slope. For the square antiprism, the dependence is noisy but positive, in agreement with that observed in the previous plots.

Let us expose a factible explanation for this behaviour based on the axial term B_0^2. In the case of the pentagonal bipyramid, a compression of the axial ligands produces an increment of the axial strain and, in consequence, a higher B_0^2 value, thus increasing the effective barrier and the blocking temperature. In apparent contrast, the regular square antiprism turns more axial when the ligands are distorted by an axial elongation, with the same consequences as the former. Note that these observations are only valid for oblate ions (Tb^{3+}, Ho^{3+}, Dy^{3+}) which are among the most usual in the dataset. In the case of prolate ions, (Er^{3+}, Tm^{3+}, Yb^{3+}) the expected behaviour would be the opposite.

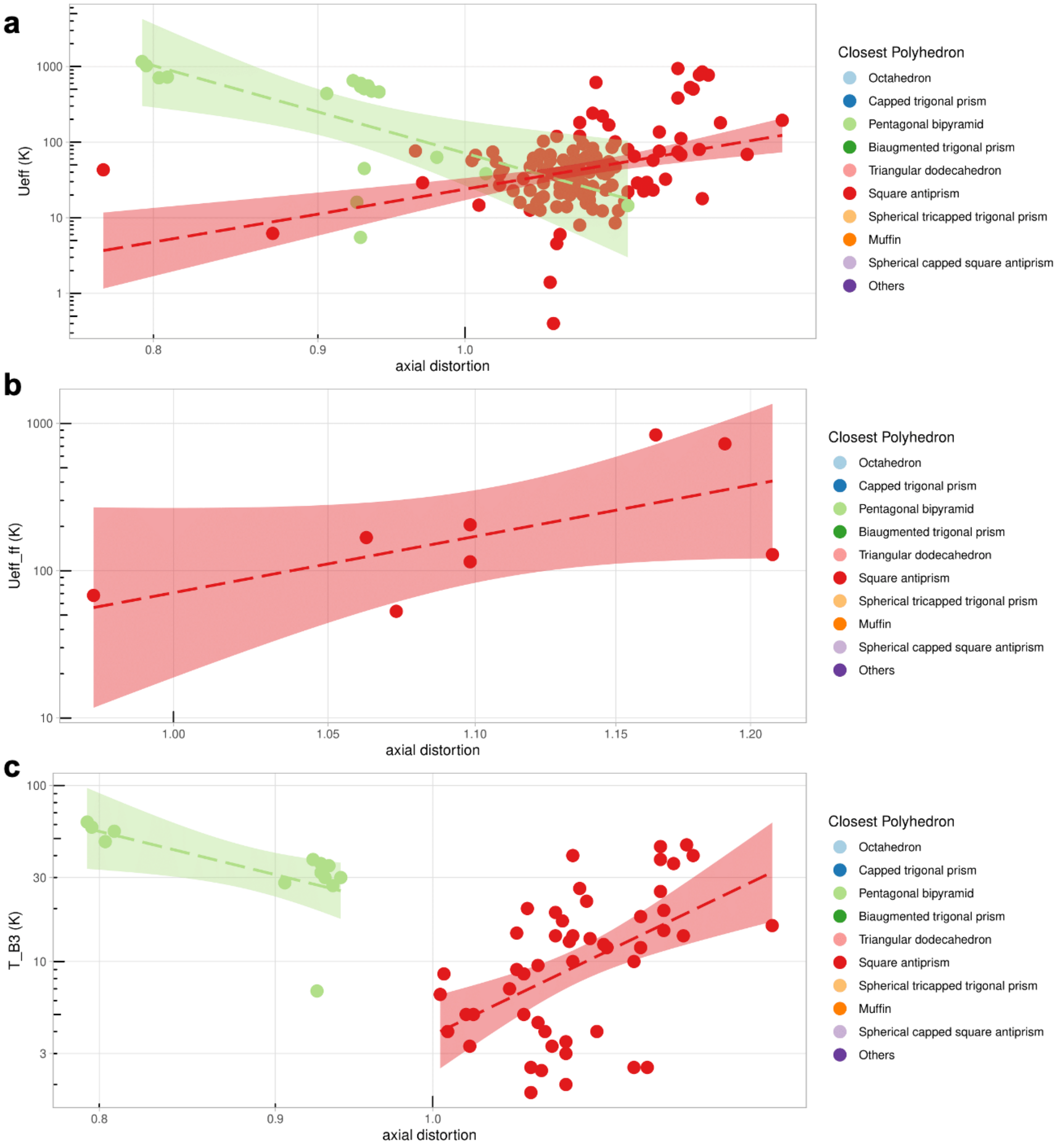

Supplementary Figure 33 | Scatter plot showing the variation vs axial distortion for U_{eff} (top), $U_{eff,ff}$ (centre), T_{B3} (bottom). The only displayed polyhedra are the ones that present a more clear dependence: square antiprism and pentagonal bipyramid. Note that not all samples will be present in all graphs (see Supplementary Figure 1.1). As a consequence, an (x vs y) plot can only include samples for which x and y are simultaneously present in the dataset.

Supplementary Section 9. Extended figures and discussion about U_{eff} vs Raman vs QTM

9.1 Investigation of the relation between different relaxation parameters

In Figure 5 of the main text we have seen an apparent correlation between $U_{eff,ff}$ and Raman relaxation prefactor C in a log-log-representation: faster relaxation via an Orbach mechanism (lower U_{eff}) happens together with faster mechanism via a Raman mechanism (higher C). As we can see in Supplementary Figure 34, the correlation is of the same sign, but more noisy, in the case of U_{eff},. Also interestingly, the correlation extends to the Raman exponent n, again with the same sign: faster relaxation at high temperature via an Orbach mechanism (lower U_{eff}) happens together with faster mechanism at high temperature via a Raman mechanism (higher n). We analyse this in much more statistical depth in the next section.

Let us start by addressing the fact of the so-called "anomalous" Raman exponents $n \neq \{7,9\}$ which are the norm rather than the exception in our dataset, and how this may be related to the meaning of the effective energy barrier U_{eff} considering an Orbach relaxation process. The matter of the anomalously low Raman exponents was recently the subject of a theoretical work by Gu *et al.*[68] First, it is important to recall that considering a pure Raman relaxation process the standard exponent at low temperatures should be either $n = 7$ (non-Kramers ions) or $n = 9$ (Kramers ions). Gu *et al.* explained why anomalies where the exponent n is in the range $3 < n < 5$ are frequently obtained by a simultaneous fitting of the relaxation rates by a 'full fit' procedure considering Raman, Orbach and direct processes, but just a single path for each mechanism. As it is known, the maximum value of the vibration energies (Debye energy) assigned to a magnetic relaxation process limits the temperature where the pure Raman process is applicable. Gu argued that, as one analyses the system above such temperature, multiple Orbach processes increase their contributions to the total rate. Indeed, the highly discrete nature of the vibrational DOS in magnetic molecules means that a sum including a single Orbach barrier U_{eff} and a single Raman contribution is an oversimplification. In particular, the low-energy nature of SIMs intra / intermolecular vibrations results in most of the magnetic measurements to be performed in the limit of the Debye energy and thus most of the measurements may be contaminated both by multiple Raman rates based on different phonons and by non-Raman rates. In a previous work[69] it was hypothesized that these values could be explained by using an optical-acoustic mechanism. However, Gu *et al.* argued that this explanation is not realistic for SIMs with a high magnetic anisotropy and a small Zeeman splitting in the ground doublet.

In any case, a plausible connection between the parameters assigned to different relaxation mechanisms can have precisely this source: the so-called "full" fit is still an oversimplification, up to the point where different parameters contaminate each other.

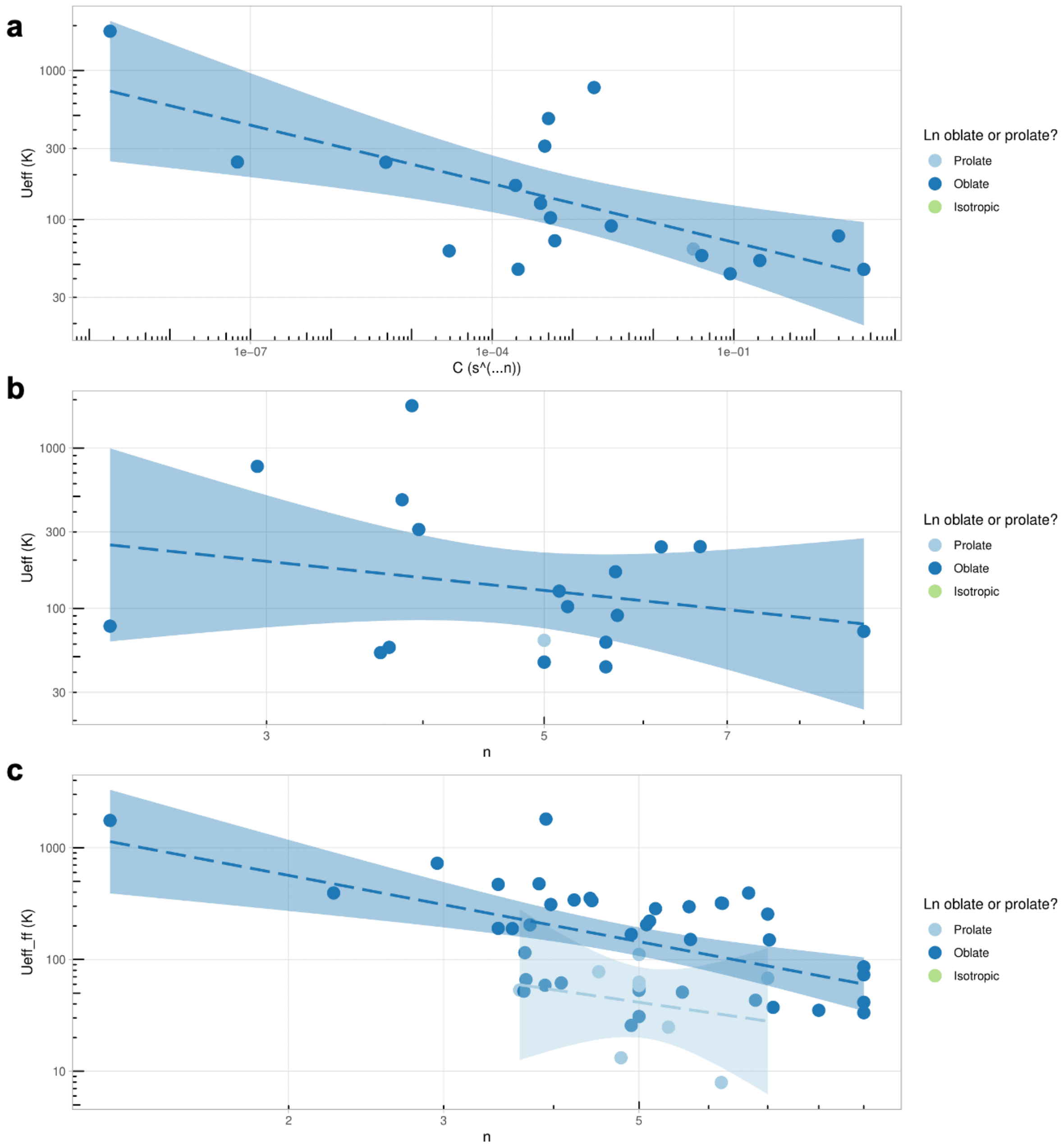

Supplementary Figure 34 | Scatter plots showing the dependence between U_{eff} and C (a), U_{eff} and n (b), $U_{eff,ff}$ and n (c) in log-log representations. The correlation with n is less clear than in the case of C. Note that not all samples will be present in all graphs (see Supplementary Figure 1.1). As a consequence, an (x vs y) plot can only include samples for which x and y are simultaneously present in the dataset.

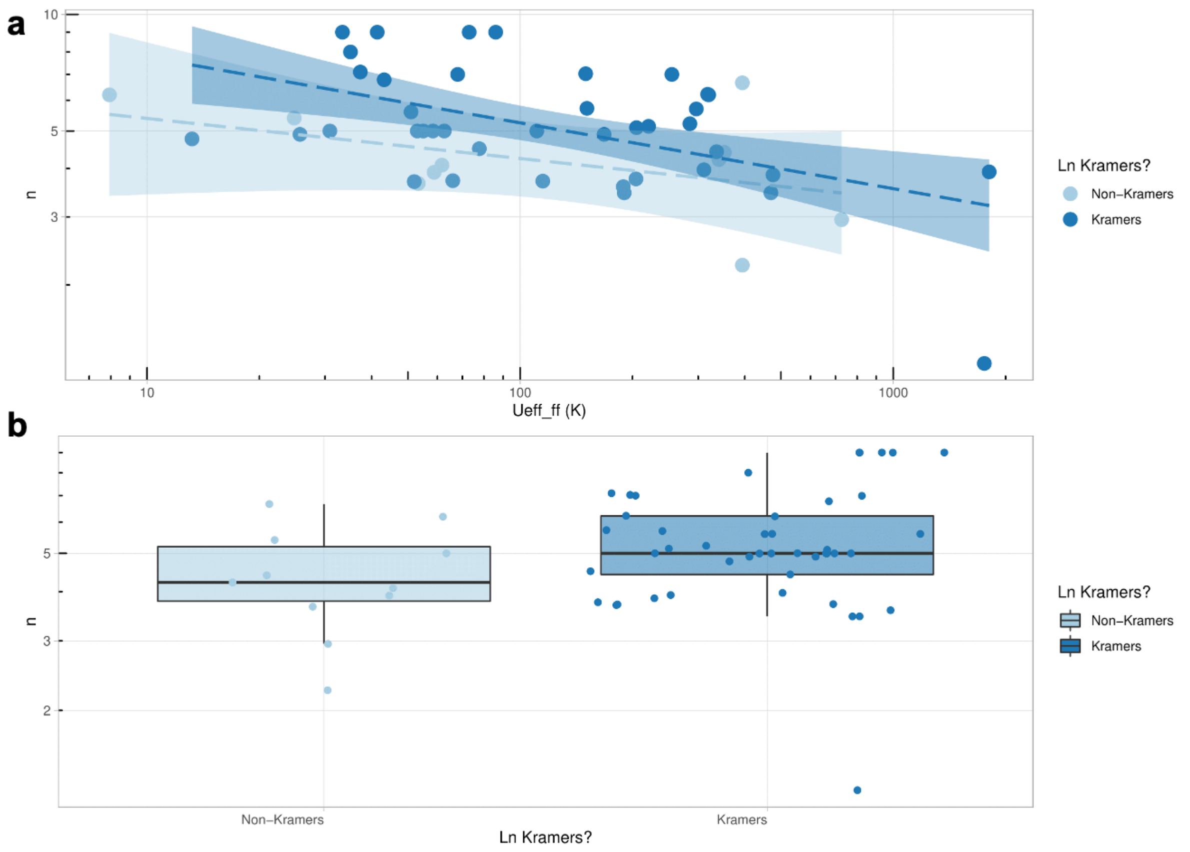

Supplementary Figure 35 | Scatter plot showing the dependence between *n* and U_{eff} (top); Boxplot showing values of *n* for Kramers and non-Kramers ions (bottom).

In Supplementary Figure 35, we represent the tendencies for both Kramers / non-Kramers ions between the Orbach barrier (U_{eff}) and the Raman exponent (n), as well as the distribution of n for Kramers vs non-Kramers ions. It is worth noting that for lower U_{eff} values, the corresponding Raman coefficients tend to the expected values 7 or 9 (9 for Kramers ions, 7 for non-Kramers). On the other hand, higher U_{eff} values are expected to produce anomalous coefficients in the range $3 < n < 5$. Again, this can be rationalised considering that for high barriers, the measuring temperatures are well over Debye energies thus producing an incorrect consideration of the Raman coefficients.

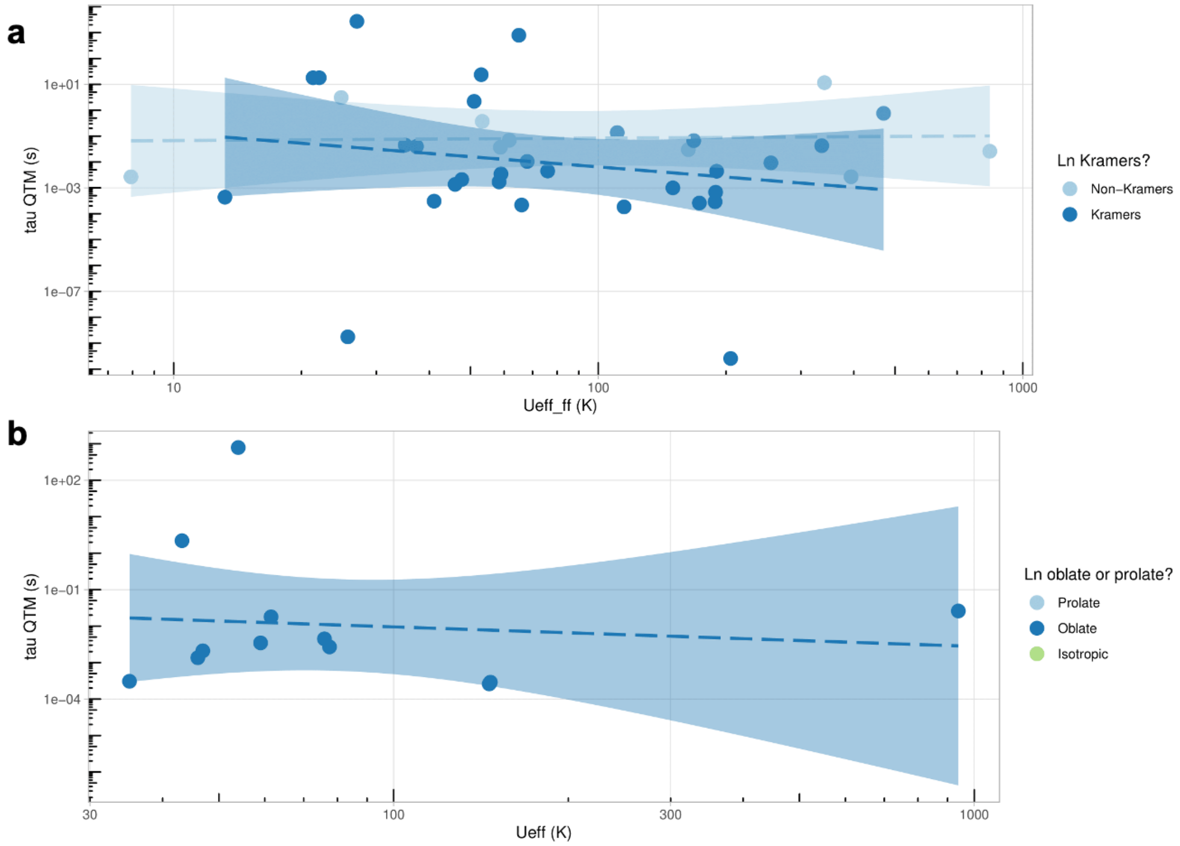

Supplementary Figure 36 | Scatter plots showing the (lack of) dependence between τ_{QTM} and U_{eff} (a), $U_{eff,ff}$ (b). There seems to be no correlation in this case.

In Supplementary Figure 36, we see the relation between τ_{QTM} and U_{eff} (top), $U_{eff,ff}$ (bottom), or rather, the lack thereof. This could be naïvely expected, of course, since Orbach and QTM are two physically independent mechanisms, but at this point it is somewhat surprising. If, as we have hypothesised, the correlation between Raman and Orbach parameters is due to so-called "full" fits being oversimplifications up to the point where different parameters contaminate each other, why should QTM be exempted from this mixing? This supports the hypothesis we expose in the main text of high values of U_{eff} being a witness for weak vibronic couplings, which also translate into low values of C, n, but do not affect QTM since phonons are not involved in QTM.

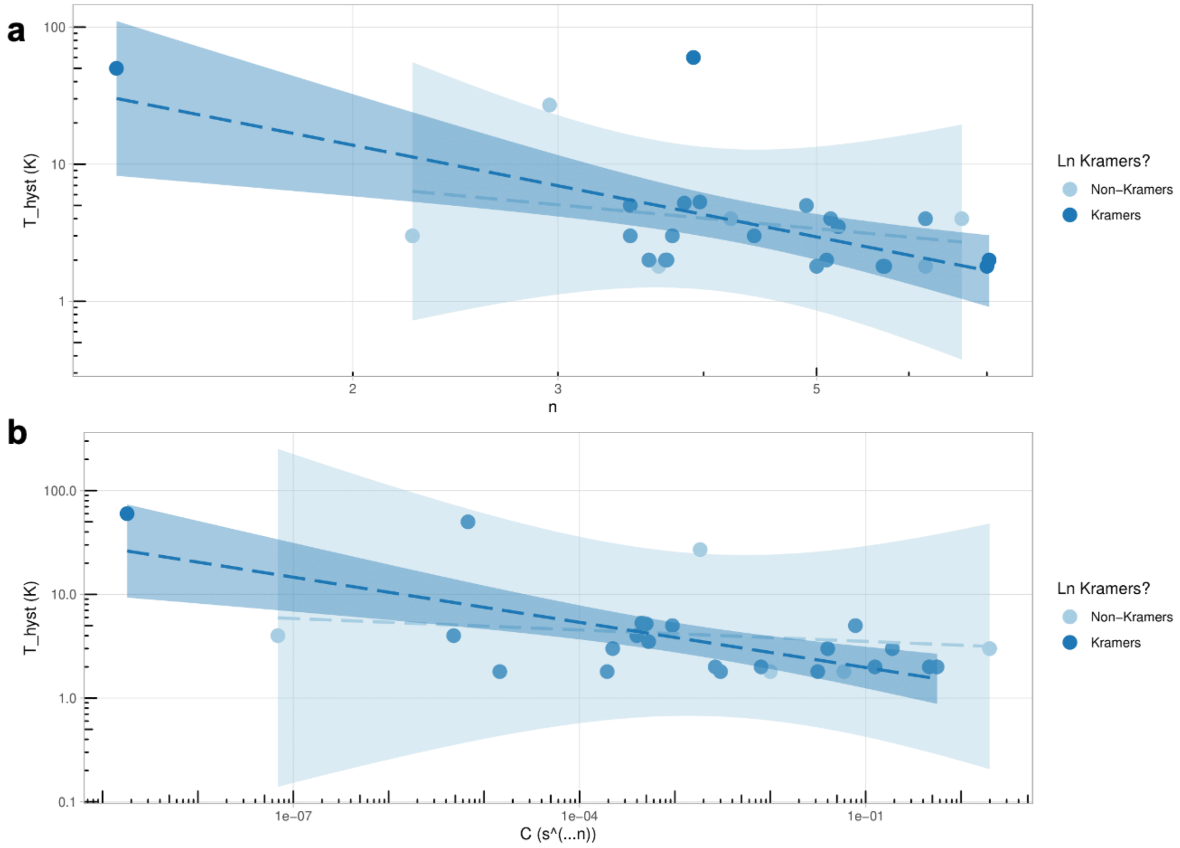

Supplementary Figure 37 | Scatter plots showing the dependence between n and T_{hyst} (a), C and T_{hyst} (b). The correlation is less clear than in the case of C. Note that not all samples will be present in all graphs (see Supplementary Figure 1.1). As a consequence, an (x vs y) plot can only include samples for which x and y are simultaneously present in the dataset.

As the last plots among physical parameters, let us discuss the relation between T_{hyst} and Raman parameters in Supplementary Figure 37. The lowering tendency of T_{hyst} with both n and C is expected, qualitatively meaning Raman relaxation mechanism needs to be weak for high hysteresis temperatures. This also supports the idea above that U_{eff} is tied to Raman and both of them are linked to magnetic behaviour.

9.2 Influence of CN and the number of ligands to optimise Raman and U_{eff}

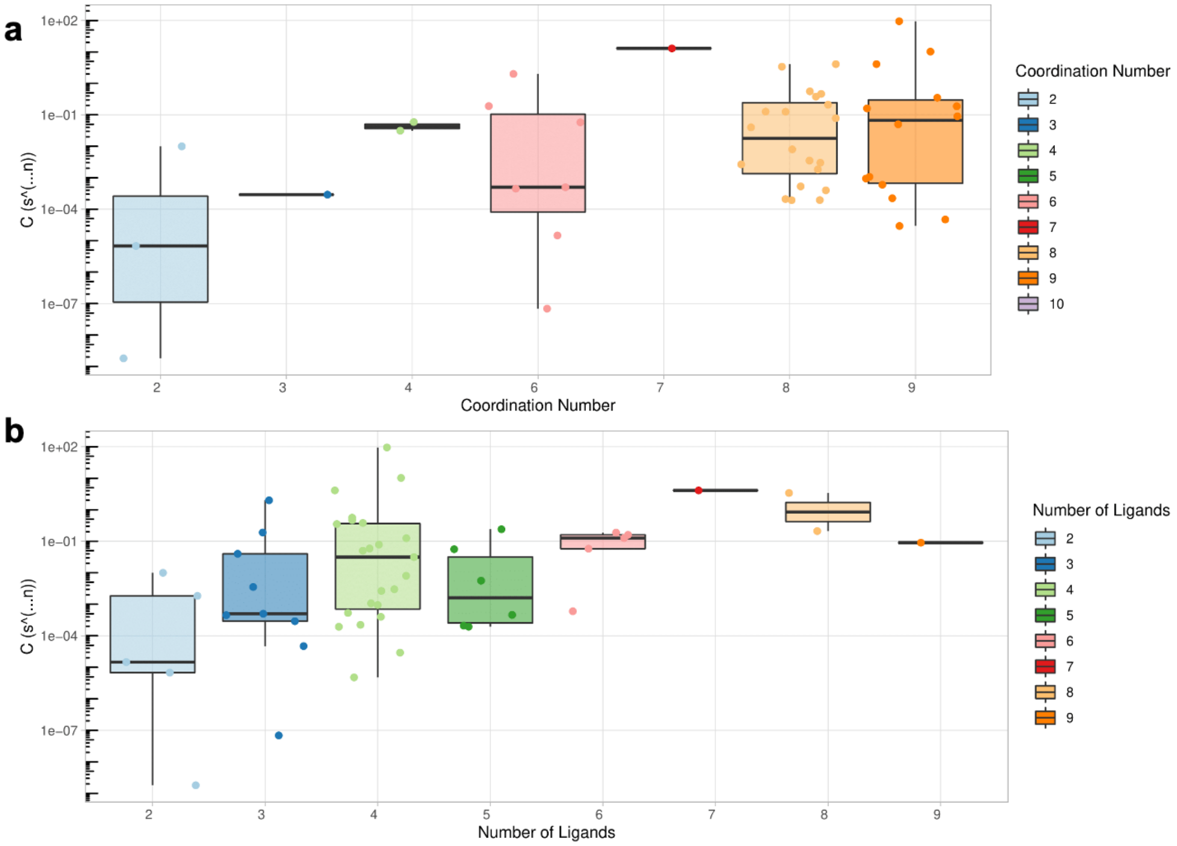

Supplementary Figure 38 | Boxplots showing the values of *C* for different coordination numbers (top) and numbers of ligands (bottom). Although the data is limited, in both cases the higher values of C_{Ra} tend to correspond to higher values of CN or numbers of ligands in the coordination sphere.

Since the Raman mechanism is governed by vibronic coupling, let us represent the evolution of *C* vs the CN and the number of ligands (see Supplementary Figure 38).

Even within very scarce data, there is an apparent tendency towards higher values of *C* vs the number of ligands and the coordination number. In the same sense but with more abundant data, there is also a tendency towards lower values of U_{eff}, with the coordination number, where CN=7 and number of ligands=7 constituting an anomaly to the general trend (Supplementary Figure 39).

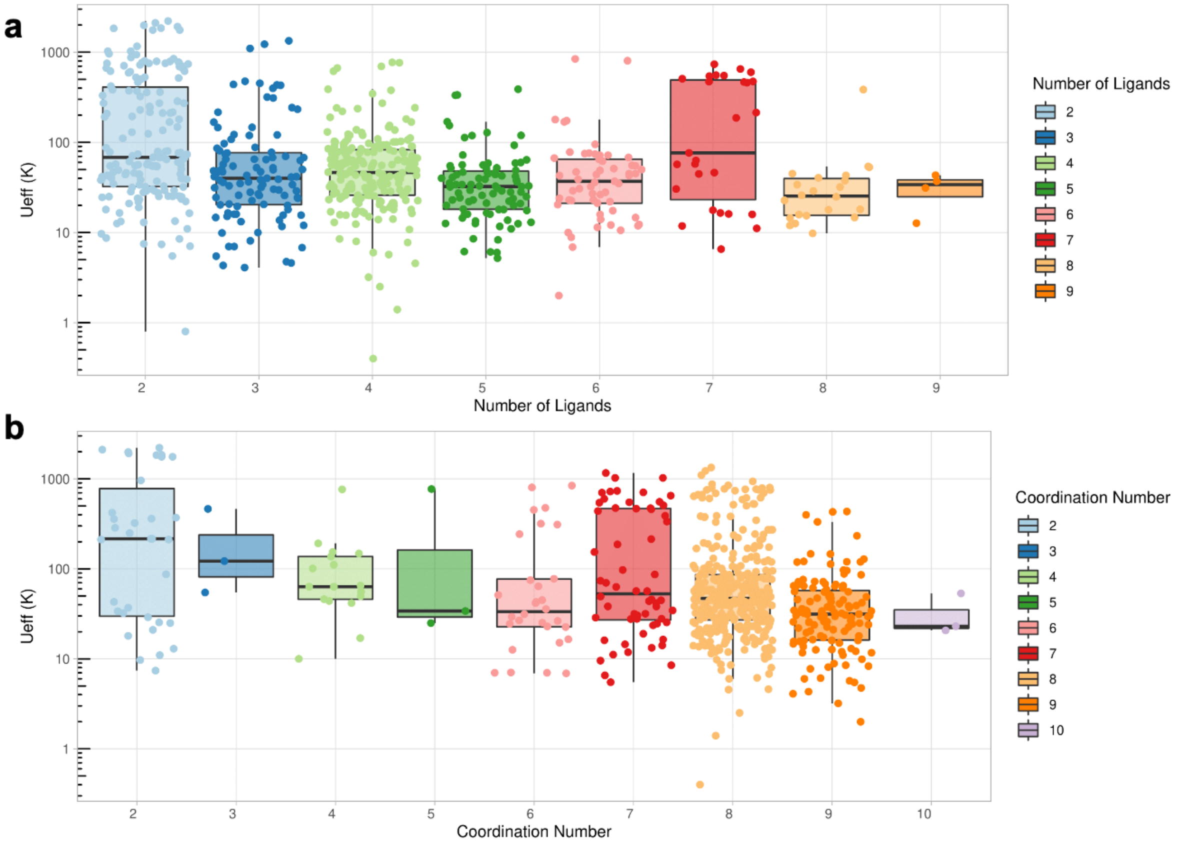

Supplementary Figure 39 | Boxplots showing the values of U_{eff} for different coordination numbers (top) and numbers of ligands (bottom). The data are more abundant, and the trend is consistent with the previous case, but less marked: in both cases the higher values of U_{eff} tend to correspond to lower values of CN or numbers of ligands in the coordination sphere, with 7 (either as CN or as number of ligands) seemingly marking an exception..

9.3 Linear relation between log($U_{\text{eff,ff}}$) vs log(C)

Since there is a seeming relation between $U_{\text{eff,ff}}$ and C when represented in log-log plots (see Figure 5 in the main text and Supplementary Figure 34), we need to verify this more thoroughly by employing statistical analysis. We show the result of the analysis in this section, and the corresponding work for $U_{\text{eff,ff}}$ vs n and $U_{\text{eff,ff}}$ vs τ_{QTM} in sections 9.4 and 9.5 respectively. Since the apparent correlation was observed in the log-log plots, we have applied a logarithmic transformation on the two variables (log10).

The linear correlation and linear regression are statistical methods that study the linear relationship between two variables:

·The correlation quantifies how closely related two variables are. There are different correlation coefficients depending on the type of data we are working with. We will use Pearson's correlation (quantitative variables with a normal distribution, although it is quite robust to non-normality). In addition to calculating the correlation coefficient, we must also calculate its significance to accept whether or not there is a correlation between the variables. Finally, we will look at the size of the associated effect, which is what we know as the coefficient of determination $R2$. It is interpreted as the amount of variance of the dependent variable explained by the independent variable. It is obtained by squaring the correlation coefficient.

·A linear regression consists of generating a model that, based on the relationship between the two variables, allows predicting the value of one from the other.

In both cases we will check the applicability conditions. We will analyse whether both variables are correlated, and if they are, we will set up the regression model.

We created sub-databases because, depending on the variable we are working with, it may have more or less missing (NA) values. We have to eliminate them in order to be able to work.

We make the graphical representation of the 46 data that we have in this case (we have eliminated one record since when applying the logarithmic transformation on zero it is infinite). For this we have used the function ggpairs of the R package GGally.[70]

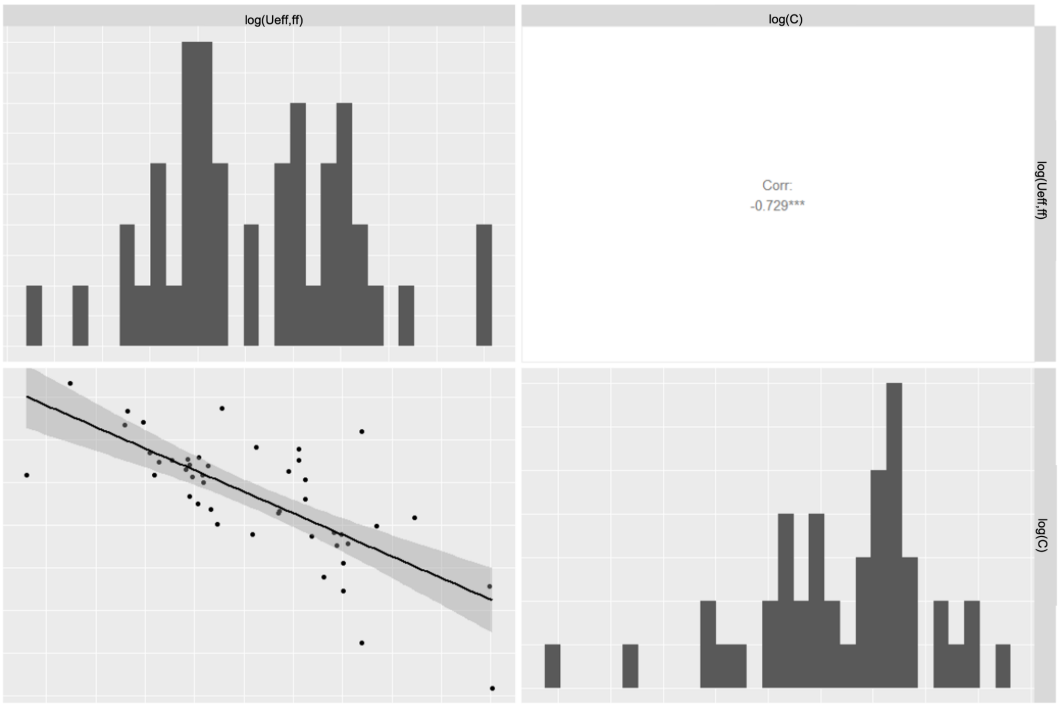

Supplementary Figure 40 | Log(U_{eff_ff}) vs log(C). From top left to bottom right: histogram with natural frequencies of U_{eff_ff} values, numerical value for the correlation, dotplot including the linear fit and histogram with natural frequencies of C.

We can see that the behaviour of the variables is very similar to a normal and that the calculated correlation coefficient is significant with a value of -0.729, which indicates that the association between the variables is high. In addition, the sign is negative, which indicates that when the values of Ueff_ff increase, the values of C decrease.

The Pearson's test result is as follows:

```
##
## Pearson's product-moment correlation
##
## data: datos1_C$logUeff_ff[-6] and datos1_C$logC[-6]
## t = -6.9775, df = 43, p-value = 1.387e-08
## alternative hypothesis: true correlation is not equal to 0
## 95 percent confidence interval:
## -0.8421097 -0.5535775
## sample estimates:
## cor
## -0.7287019
```

The coefficient of determination associated with the correlation coefficient is:

```
## [1] 0.5310065
```

Next we run the linear model. We obtain the following output:

```
## Estimate Std. Error Lower 95% Upper 95% P-value
## (Intercept) 4.251 0.922 2.392 6.11 <0.001

## logUeff_ff -3.038 0.435 -3.916 -2.16 <0.001
## R Squared 0.531
## Adj.R Squared 0.5201
```

We see that the estimate obtained for Ueff_ff is -3.038 and that it is significant. This means that: a one-unit increase in the predictor log(Ueff_ff) causes an average change of -3.038 units in the response variable log(*C*) while keeping all the other predictors constant (in this case we have no more predictors). In the same way as with the calculation of the correlation coefficient, the relationship is inverse.

Let us check the applicability conditions. From the model we obtain the following graphs:

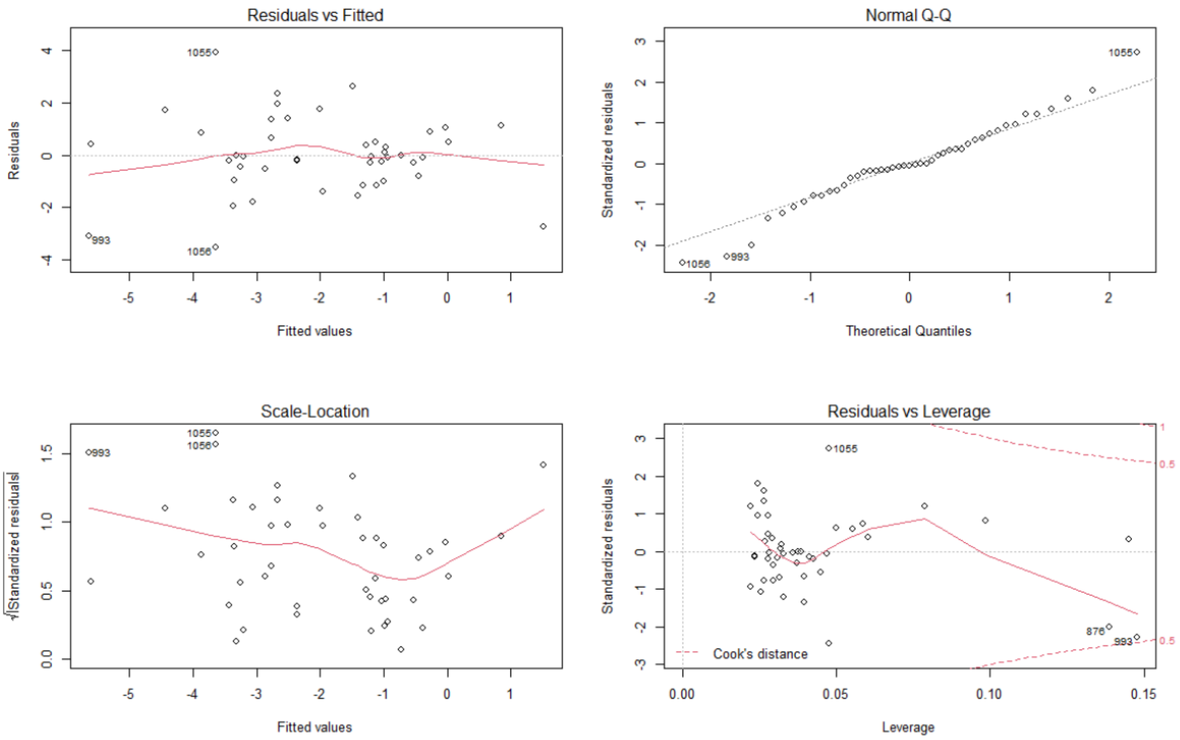

Supplementary Figure 41 | Applicability conditions for the relation between log(U_{eff_ff}) vs log(C). From top left to bottom right: residuals vs fitted values, standardised residuals vs their theoretical values, square root of the standardised residuals vs the fitted values and standardised residuals vs leverage.

Visually, it seems that the condition of linearity and normality of the residuals is fulfilled, but let us check them all:

- **Linearity:**

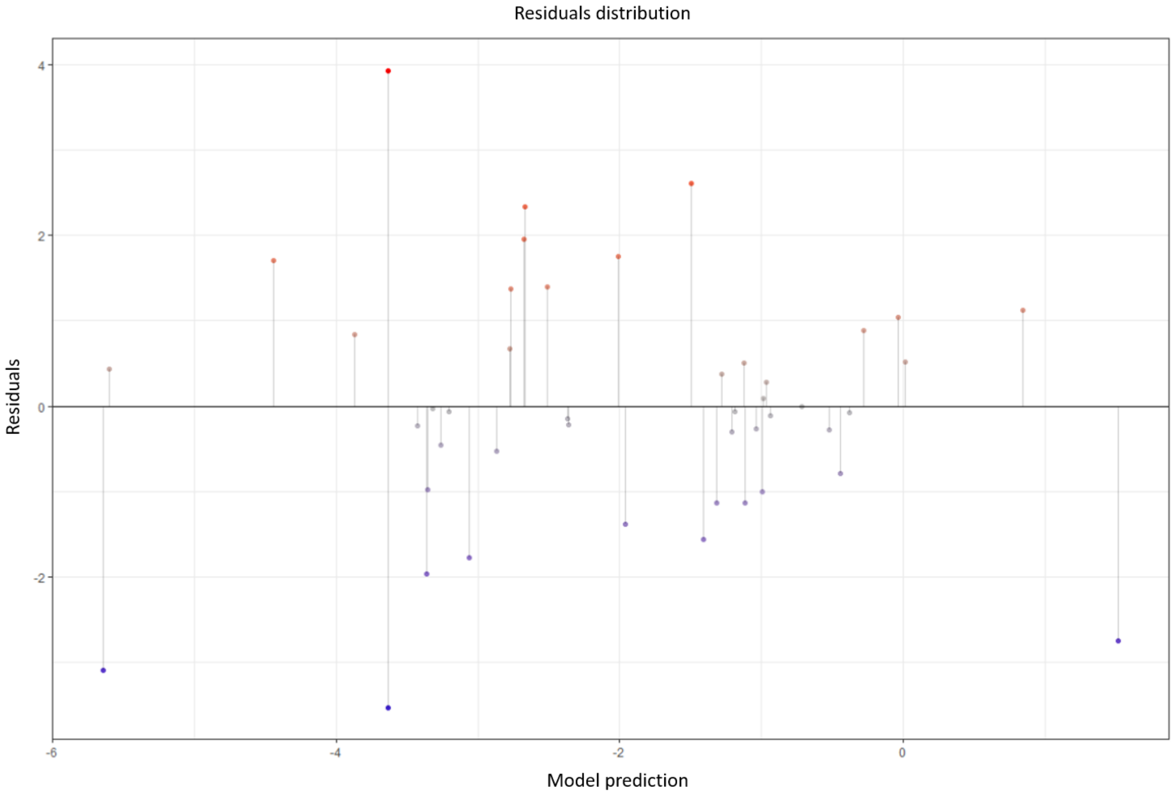

Supplementary Figure 42 | Linearity condition of the model. Value of the residual vs the prediction of the model.

The residuals are randomly distributed around 0 so linearity is accepted.

- **Normality:**

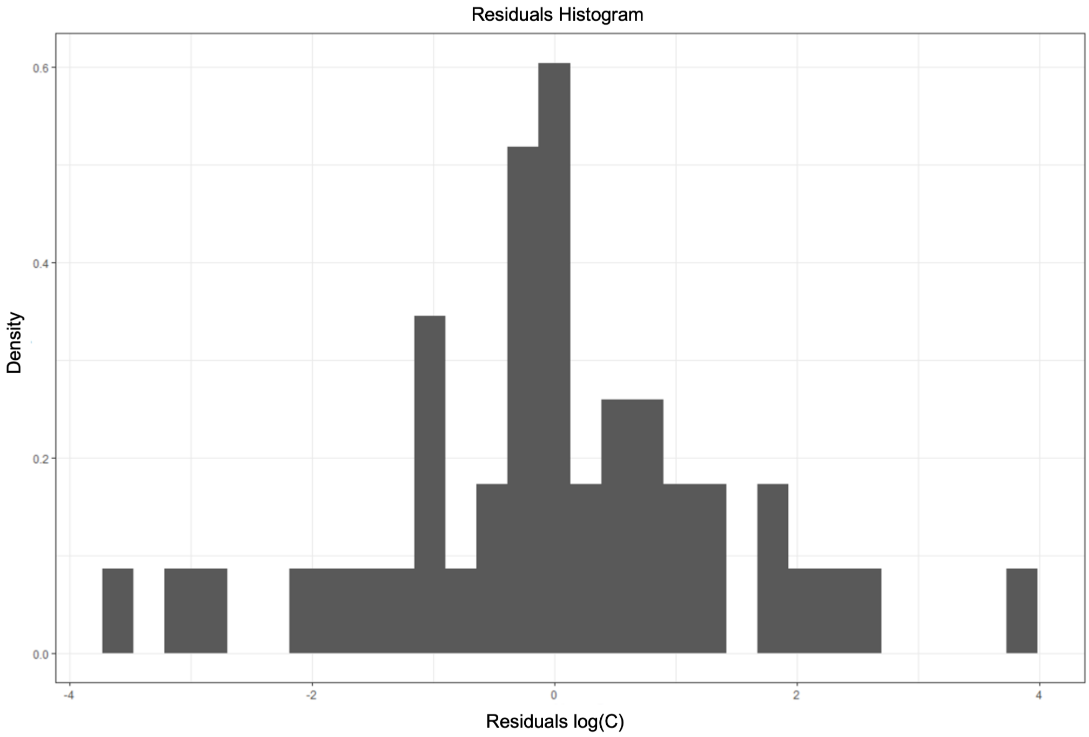

Supplementary Figure 43 | Normality condition of the model. Frequency density vs residuals.

Let's carry out the corresponding test to check it:

```
##
## Shapiro-Wilk normality test
##
## data: mod_C1$residuals
## W = 0.98134, p-value = 0.6743
```

The p-value obtained is not significant, so we are not in a position to reject the normality hypothesis, so the normal distribution of the residuals is confirmed.

- **Homogeneity of variance:**

We performed the Breusch-Pagan test to check for this:

```
##
## studentized Breusch-Pagan test
##
## data: mod_C1
## BP = 3.6016, df = 1, p-value = 0.05772
```

The p-value obtained is not significant, so we accept the condition of homogeneity of variances.

- **Autocorrelation of residuals:**

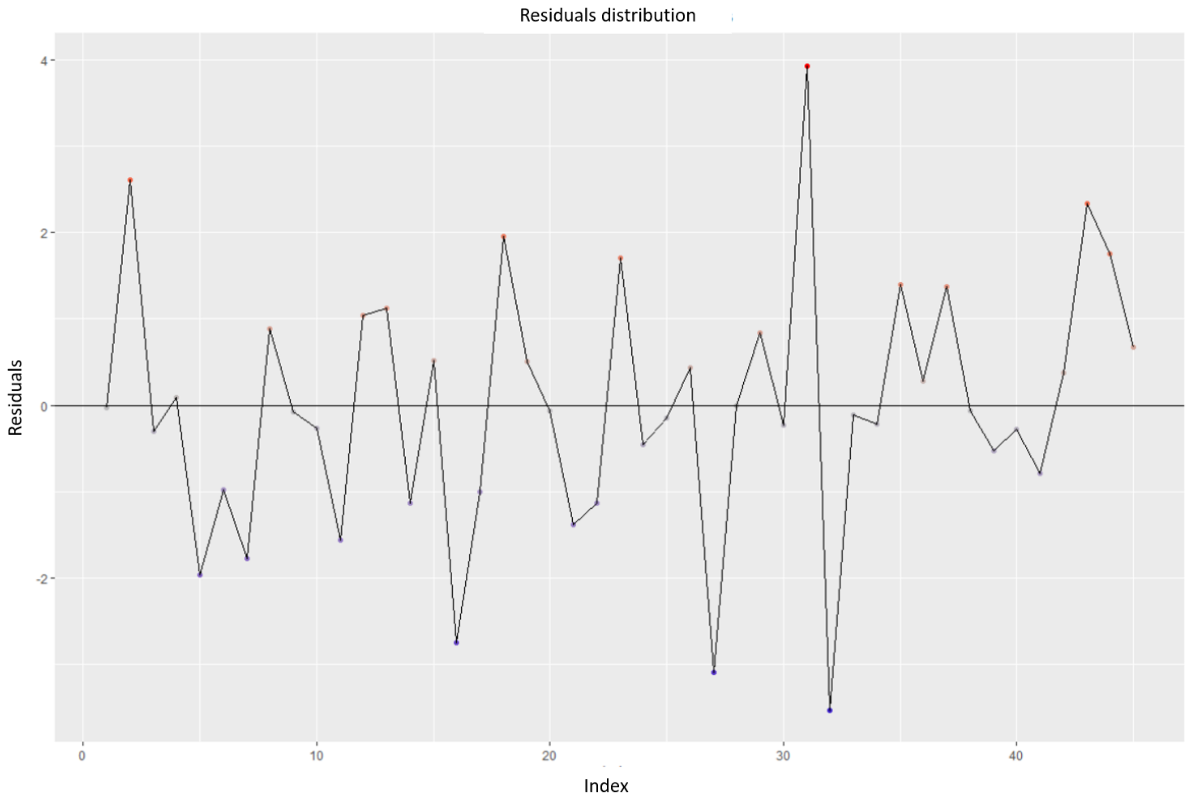

Supplementary Figure 44 | Autocorrelation of the residuals. Values of the residuals vs their index.

The representation of the residuals does not show any trend.

- **Outliers:**

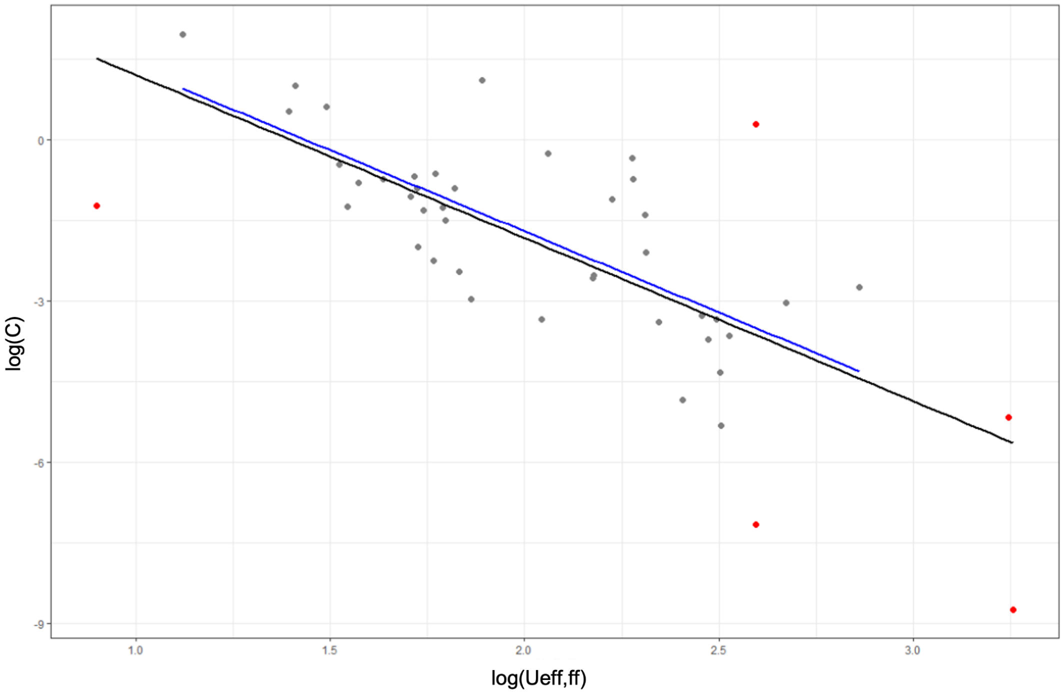

Supplementary Figure 45 | Outlier in log(U_{eff_ff}) vs log(C). Scatter diagram. Outliers are marked in red.

We have identified the outliers and recalculated the least squares linear regression. The plot shows that there is practically no change with or without the outliers.

In conclusion, all conditions to apply a least squares linear regression model are met and that the obtained p-value indicates a significant association for both variables. The adjusted R^2 is 52%.

9.4 Linear relation between $\log(U_{\text{eff,ff}})$ vs $\log(n)$

We make the graphical representation of the 50 data in this case. We have used, as in the previous section, the function ggpairs of the R package GGally.[70]

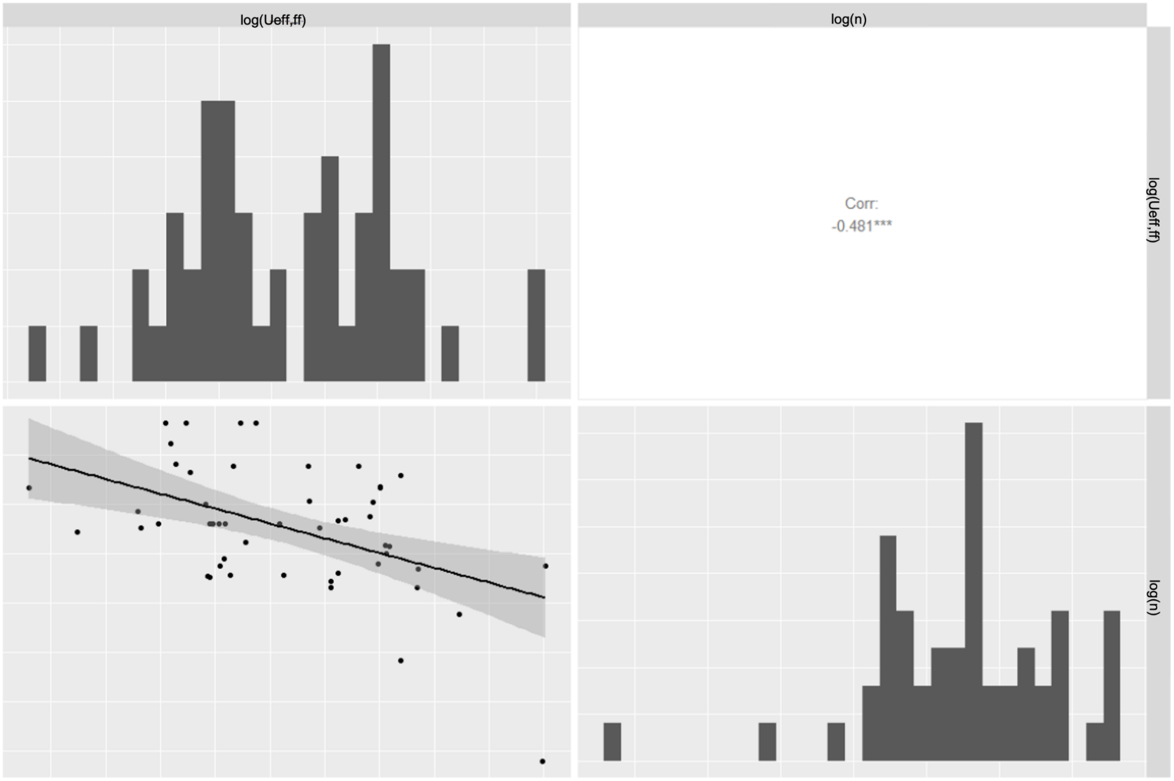

Supplementary Figure 46 | $\text{Log}(U_{\text{eff,ff}})$ vs $\log(n)$. From top left to bottom right: histogram with natural frequencies of $U_{\text{eff\_ff}}$ values, numerical value for the correlation, dotplot including the linear fit and histogram with natural frequencies of *n*.

It is shown that the behaviour of the variables is very similar to a normal distribution and that the calculated correlation coefficient is significant with a value of -0.481. This indicates that the association between the variables is moderate. Furthermore, the sign is negative, which means that when the values of Ueff_ff increase, consequently the values of *n* decrease.

The Pearson's test is as follows:

```
##
## Pearson's product-moment correlation
##
## data: datos1_n$logUeff_ff and datos1_n$logn
## t = -3.8043, df = 48, p-value = 0.0004023
## alternative hypothesis: true correlation is not equal to 0
## 95 percent confidence interval:
## -0.6699142 -0.2343687
## sample estimates:
## cor
## -0.4813173
```

The coefficient of determination associated with the correlation coefficient is:

```
## [1] 0.2316663
```

Next we run the linear model. We obtain the following output:

```
## Estimate Std. Error Lower 95% Upper 95% P-value
## (Intercept) 1.001 0.084 0.831 1.17 <0.001

## logUeff_ff -0.15 0.039 -0.229 -0.071 <0.001
## R Squared 0.2317
## Adj.R Squared 0.2157
```

We can see that the estimate obtained for log(Ueff_ff) is -0.15 and that it is significant. This indicates that an increase of one-unit in the predictor (log(Ueff_ff)) causes an average change of -0.15 units in the response variable (in this case is log(*n*)) while keeping all the other predictors constant (in this case we have no more predictors). Besides as with the calculation of the correlation coefficient, the relationship is inverse.

Let us check the applicability conditions. From the model we obtain the following graphs:

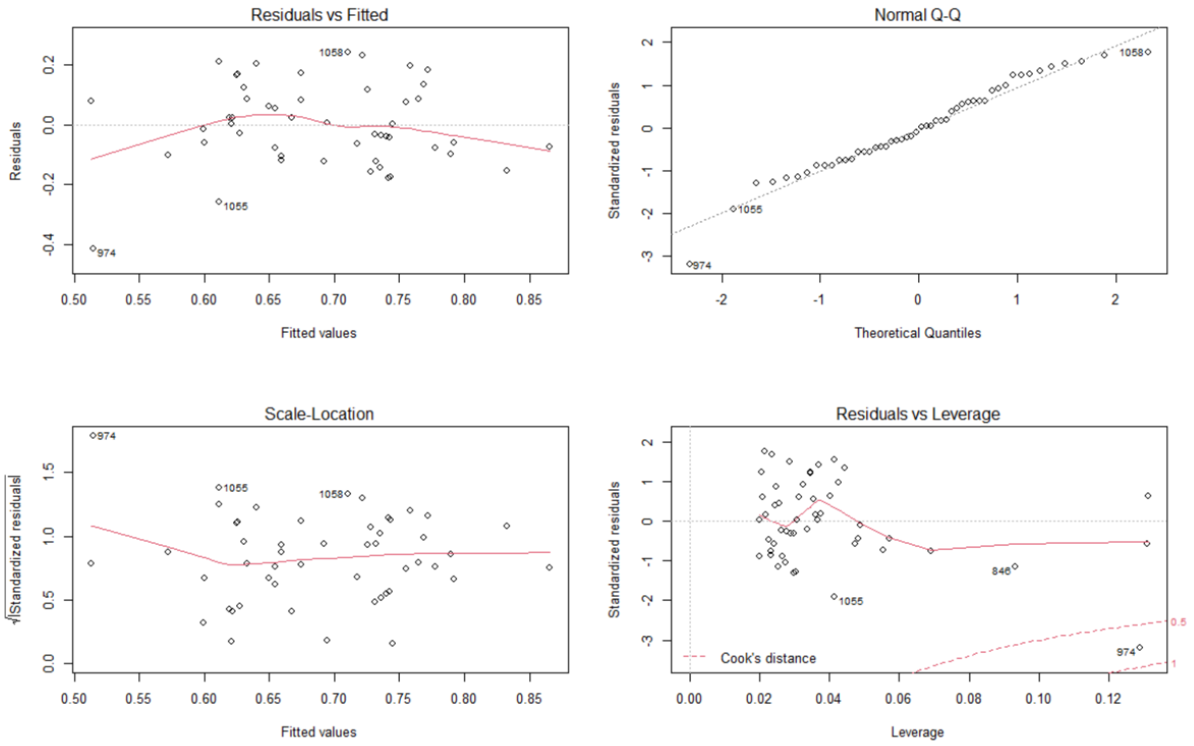

Supplementary Figure 47 | Applicability conditions for the relation between log($U_{eff,ff}$) vs log(n). From top left to bottom right: residuals vs fitted values, standardised residuals vs their theoretical values, square root of the standardised residuals vs the fitted values and standardised residuals vs leverage.

- **Linearity:**

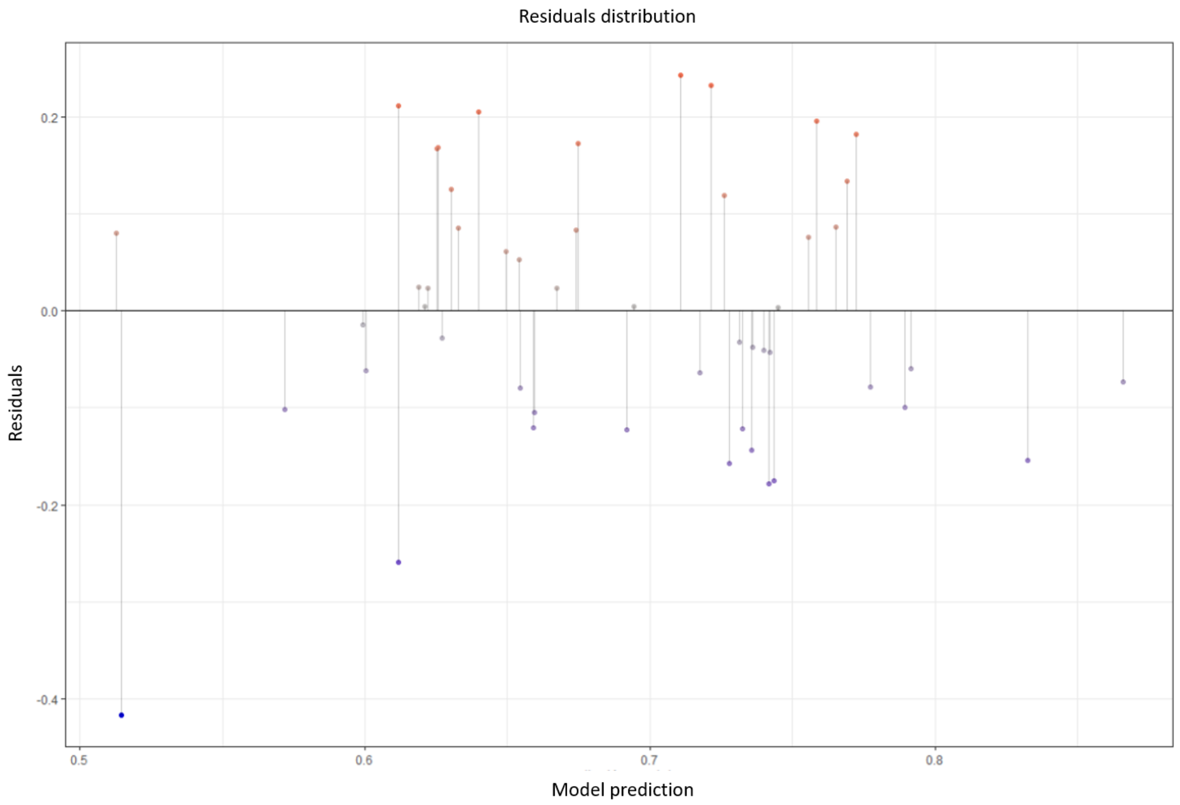

Supplementary Figure 48 | Linearity condition of the model. Value of the residual vs the prediction of the model.

The residuals are randomly distributed around 0 so linearity is accepted.

- **Normality:**

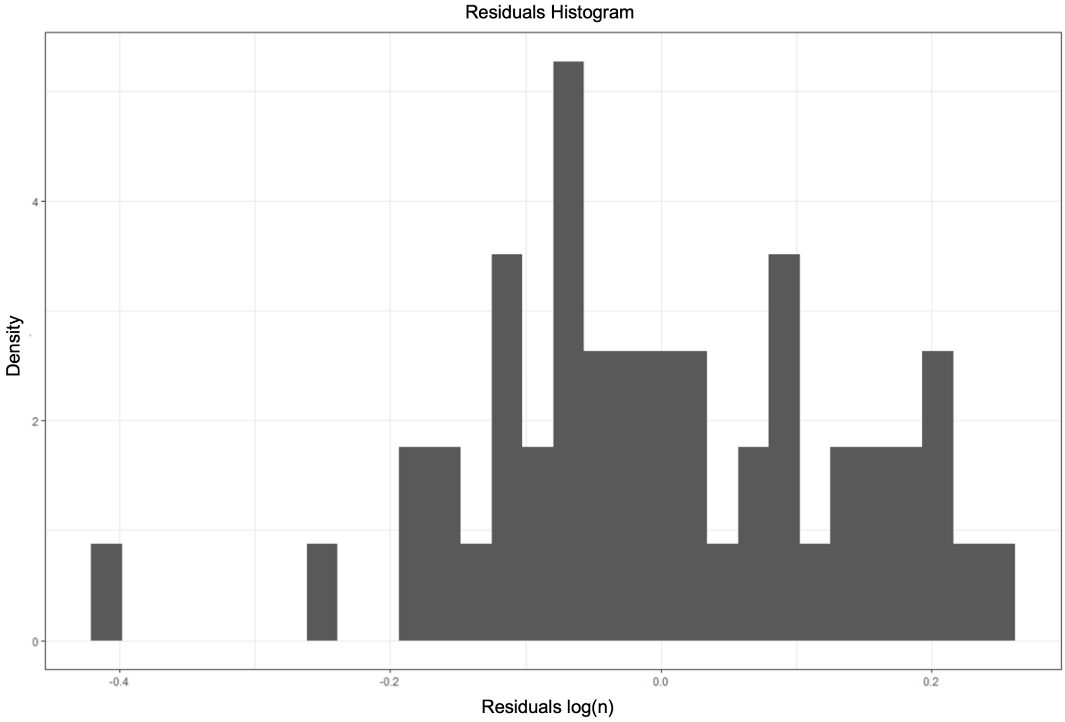

Supplementary Figure 49 | Normality condition of the model. Frequency density vs residuals.

Then we carry out the corresponding test:

```
##
## Shapiro-Wilk normality test
##
## data: mod_n1$residuals
## W = 0.97245, p-value = 0.2904
```

The p-value obtained is not significant, so we are not in a position to reject the normality hypothesis, so the normal distribution of the residuals is confirmed.

- **Homogeneity of variance:**

We performed the Breush-Pagan test to check for this:

```
##
## studentized Breusch-Pagan test
##
## data: mod_n1
## BP = 3.3668, df = 1, p-value = 0.06652
```

The p-value obtained is non-significant, so we accept the condition of homogeneity of variances.

- **Autocorrelation of residuals:**

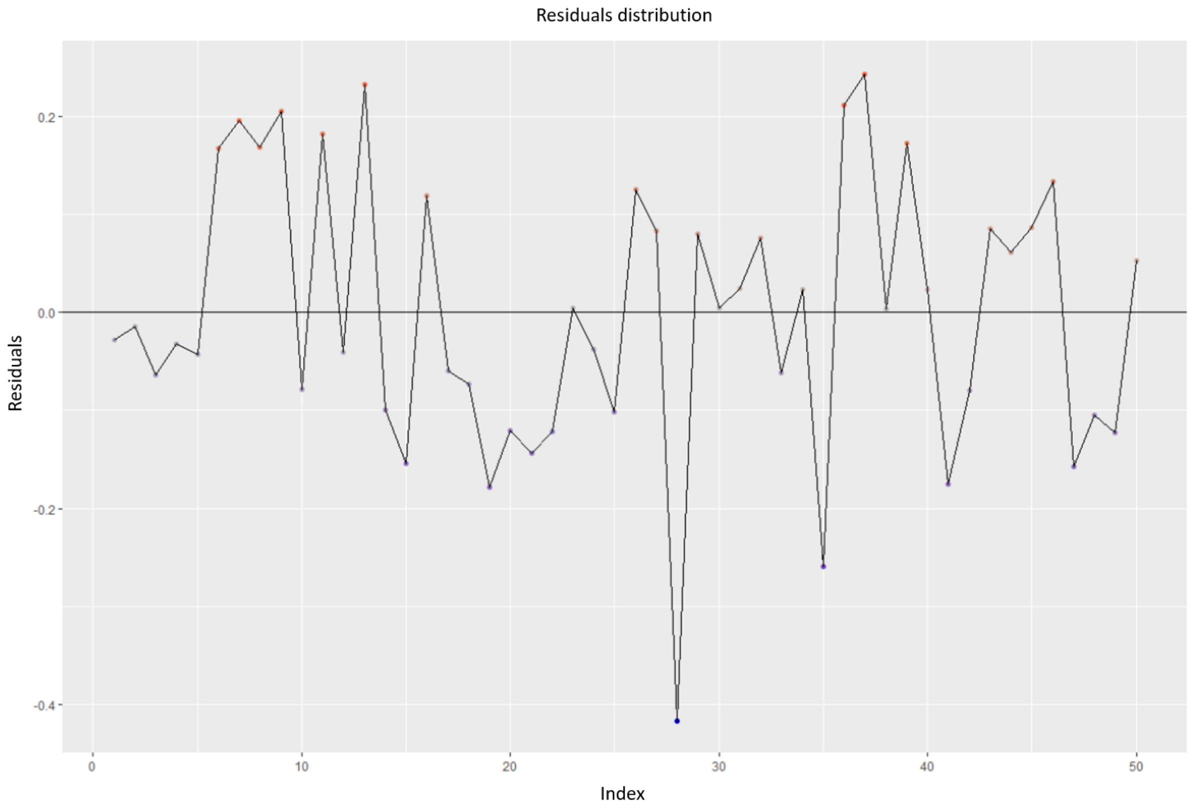

Supplementary Figure 50 | Autocorrelation of the residuals. Values of the residuals vs their index.

The representation of the residuals does not show any trend.

- **Outliers:**

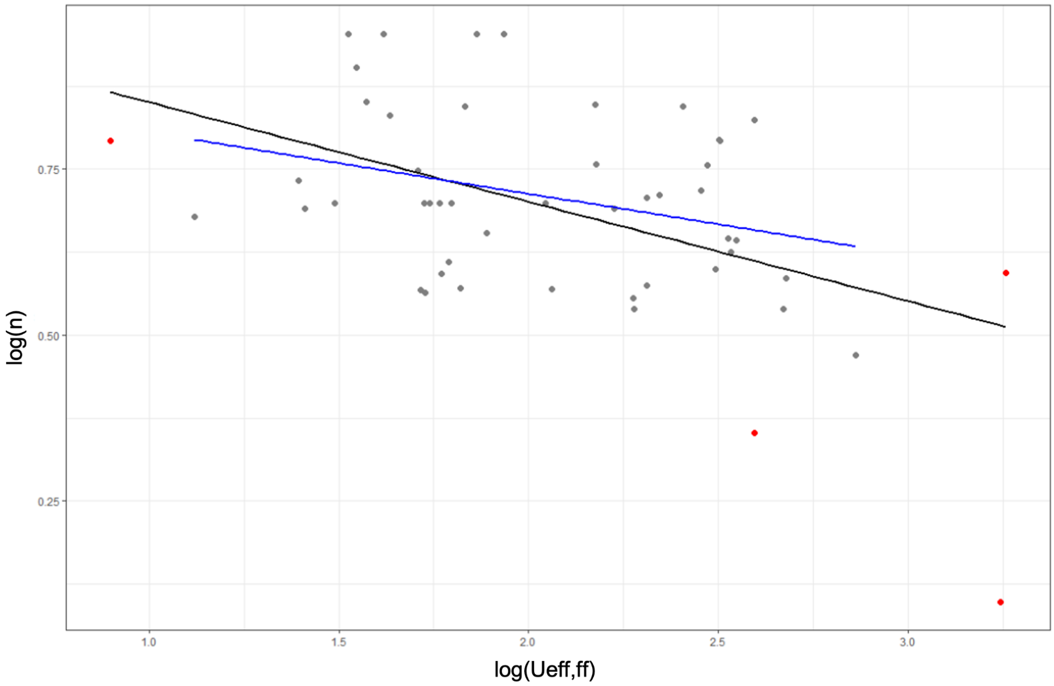

Supplementary Figure 51 | Outlier in log(n) vs log($U_{eff,ff}$). Scatter diagram. Outliers are marked in red

We have identified the outliers and recalculated the least squares linear regression. A change in the slope can be seen. Furthermore, if we remove the outliers the calculated estimation changes from -0.15 to -0.09.

In conclusion, all conditions to apply a least squares linear regression model are met, and the obtained p-value indicates a significant association for both variables. The adjusted R^2 is 21.5%.

9.5 Linear relation between log($U_{eff,ff}$) vs log(τ_{QTM})

Next, we make the graphical representation of the 58 entries with values of τ_{QTM}. We have used, as in the previous section, the function ggpairs of the R package GGally.[70]

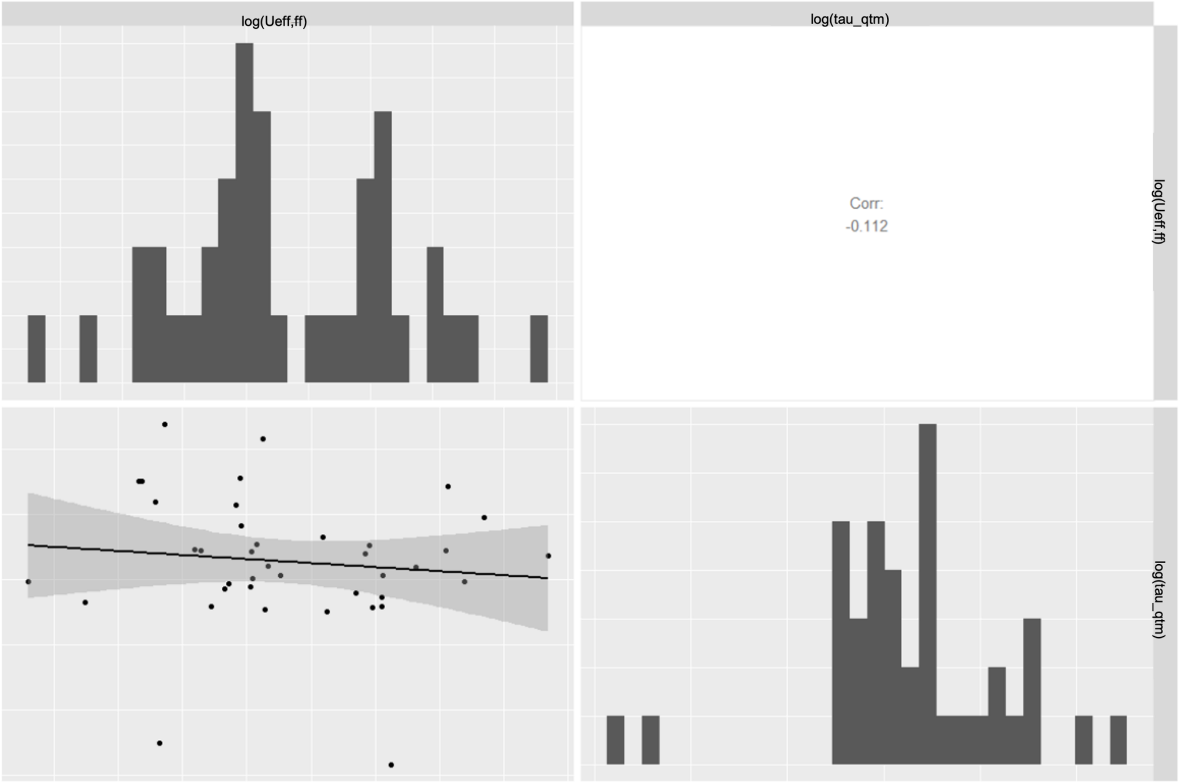

Supplementary Figure 52 | Log($U_{eff,ff}$) vs log(τ_{QTM}). From top left to bottom right: histogram with natural frequencies of U_{eff_ff} values, numerical value for the correlation, dotplot including the linear fit and histogram with natural frequencies of *n*.

We can see that the behaviour of the variables is very similar to a normal distribution and that the calculated correlation coefficient is not significant and small. This means either there is not an association between the variables or it is very small.

The Pearson's test is as follows:

```
##
## Pearson's product-moment correlation
##
## data: datos1_tauqtm$logUeff_ff and datos1_tauqtm$logtau_qtm
## t = -0.68349, df = 37, p-value = 0.4986
## alternative hypothesis: true correlation is not equal to 0
## 95 percent confidence interval:
## -0.4126421 0.2112983
## sample estimates:
## cor
## -0.1116631
```

We can see that the association is non-significant so that it makes no sense to test a linear model with the variables.

Anyhow, we show the result of the model:

```
## Estimate Std. Error Lower 95% Upper 95% P-value
## (Intercept) -0.634 1.777 -4.234 2.967 0.723
## logUeff_ff -0.62 0.907 -2.459 1.218 0.499
## R Squared 0.0125
## Adj.R Squared -0.0142
```

We can see that the calculated estimate for Ueff_ff is non-significant.

Supplementary Section 10. Evidence for/against the main tested hypotheses

Supplementary Table 7 | Main hypotheses in the present studies and location of the evidence for or against each of them.

hypothesis	answer	evidence
$LnPc_2$ distinctly promising as SIMs?	yes	·Fig. 3 a,b<br>·SI Figs 11.1, 11.6, 12.1, 12.2<br>·SI Sect. 6.1 (magnetostructural clustering analysis)
metallocenes distinctly promising as SIMs?	yes	
any other family among {Schiff bases, polyoxometalates, diketonates, radicals, TM near Ln} promising as SIMs?	no	
oblate (Dy,Tb…) > prolate (Er…) ? (ac, U_{eff})	yes	·Fig 3c<br>·SI Fig 11.3
oblate (Dy,Tb…) > prolate (Er…) ? (hysteresis)	no	·Fig 3d<br>·SI Fig 11.5
U_{eff} an excellent predictor?	yes	·Fig 5<br>·SI Sect. 5
U_{eff} correlated with τ_0?	yes	·SI Figs. 24.1, 24.2<br>·SI Tabl. 5
U_{eff} independent of Raman?	no !	·Fig 5<br>·SI Sect 9
any promising underexplored coordination polyhedron?	yes ! (compressed) pentagonal bipyramid	·Fig 6b,6c,6d<br>·SI Sect 7<br>·SI Sect 8

Supplementary References

1. Giansiracusa, M. J., Kostopoulos, A. K., Collison, D., Winpenny, R. E. P. & Chilton, N. F. Correlating blocking temperatures with relaxation mechanisms in monometallic single-molecule magnets with high energy barriers (Ueff > 600 K). *Chem. Commun.* **55**, 7025–7028 (2019).
2. Sutton, A. J., Duval, S. J., Tweedie, R. L., Abrams, K. R. & Jones, D. R. Empirical assessment of effect of publication bias on meta-analyses. *BMJ* **320**, 1574–1577 (2000).
3. Ishikawa, N., Sugita, M., Ishikawa, T., Koshihara, S. & Kaizu, Y. Lanthanide Double-Decker Complexes Functioning as Magnets at the Single-Molecular Level. *J. Am. Chem. Soc.* **125**, 8694–8695 (2003).
4. AlDamen, M. A., Clemente-Juan, J. M., Coronado, E., Martí-Gastaldo, C. & Gaita-Ariño, A. Mononuclear Lanthanide Single-Molecule Magnets Based on Polyoxometalates. *J. Am. Chem. Soc.* **130**, 8874–8875 (2008).
5. AlDamen, M. A. *et al.* Mononuclear Lanthanide Single Molecule Magnets Based on the Polyoxometalates $[Ln(W_5O_{18})_2]^{9-}$ and $[Ln(\beta_2\text{-}SiW_{11}O_{39})_2]^{13-}$($Ln^{III}$ = Tb, Dy, Ho, Er, Tm, and Yb). *Inorg. Chem.* **48**, 3467–3479 (2009).
6. Cardona-Serra, S. *et al.* Lanthanoid Single-Ion Magnets Based on Polyoxometalates with a 5-fold Symmetry: The Series $[LnP_5W_{30}O_{110}]^{12-}$ (Ln^{3+} = Tb, Dy, Ho, Er, Tm, and Yb). *Journal of the American Chemical Society* **134**, 14982–14990 (2012).
7. Li, D.-P. *et al.* Single-ion magnets based on mononuclear lanthanide complexes with chiral Schiff base ligands [Ln(FTA)3L] (Ln = Sm, Eu, Gd, Tb and Dy). *Chem. Commun.* **46**, 2929–2931 (2010).
8. Li, X., Li, T., Shi, X. J. & Tian, L. A family of 2p-4f complexes based on indazole radical: Syntheses, structures and magnetic properties. *Inorganica Chim. Acta* **456**, 216–223 (2017).
9. Wang, Y., Li, X.-L., Wang, T.-W., Song, Y. & You, X.-Z. Slow Relaxation Processes and Single-Ion Magnetic Behaviors in Dysprosium-Containing Complexes. *Inorganic Chemistry* **49**, 969–976 (2010).
10. Mei, X.-L., Ma, Y., Li, L.-C. & Liao, D.-Z. Ligand field-tuned single-molecule magnet behaviour of 2p–4f complexes. *Dalton Trans.* **41**, 505–511 (2011).
11. Mei, X.-L. *et al.* Modulating spin dynamics of cyclic LnIII-radical complexes (Ln^{III} = Tb, Dy) by using phenyltrifluoroacetylacetonate coligand. *Dalton Transactions* **41**, 2904 (2012).
12. Hu, P. *et al.* Single-molecule magnets based on rare earth complexes with chelating benzimidazole-substituted nitronyl nitroxide radicals. *Dalton Transactions* **41**, 14651 (2012).
13. Murakami, R., Ishida, T., Yoshii, S. & Nojiri, H. Single-molecule magnet $[Tb(hfac)_3(2pyNO)]$ (2pyNO = t-butyl 2-pyridyl nitroxide) with a relatively high barrier of magnetization reversal. *Dalton Transactions* **42**, 13968 (2013).
14. Hu, P. *et al.* A new family of Ln–radical chains (Ln = Nd, Sm, Gd, Tb and Dy): synthesis, structure, and magnetic properties. *Dalton Trans.* **43**, 2234–2243 (2014).
15. Li, C. *et al.* From Monomeric Species to One-Dimensional Chain: Enhancing Slow Magnetic Relaxation through Coupling Mononuclear Fragments in Ln-rad System. *Crystal Growth & Design* **16**, 7155–7162 (2016).
16. Tretyakov, E. V. *et al.* Complexes of lanthanides with spin-labeled pyrazolylquinoline. *Russ. Chem. Bull.* **63**, 1459–1464 (2014).
17. Dolinar, B. S., Gómez-Coca, S., Alexandropoulos, D. I. & Dunbar, K. R. An air stable radical-bridged dysprosium single molecule magnet and its neutral counterpart: redox switching of magnetic relaxation dynamics. *Chemical Communications* **53**, 2283–2286 (2017).
18. Sun, W.-B. *et al.* The slow magnetic relaxation regulated by ligand conformation of a lanthanide single-ion magnet $[Hex_4N][Dy(DBM)_4]$. *Inorg. Chem. Front.* **1**, 503–509 (2014).
19. Zeng, D., Ren, M., Bao, S.-S. & Zheng, L.-M. Tuning the Coordination Geometries and

Magnetic Dynamics of $[Ln(hfac)_4]^-$ through Alkali Metal Counterions. *Inorg. Chem.* **53**, 795–801 (2014).
20. Cen, P. *et al.* Capping N-Donor Ligands Modulate the Magnetic Dynamics of Dy^{III} β-Diketonate Single-Ion Magnets with D 4 d Symmetry. *Chemistry – A European Journal* **25**, 3884–3892 (2019).
21. Ishikawa, N., Mizuno, Y., Takamatsu, S., Ishikawa, T. & Koshihara, S. Effects of Chemically Induced Contraction of a Coordination Polyhedron on the Dynamical Magnetism of Bis(phthalocyaninato)disprosium, a Single-4f-Ionic Single-Molecule Magnet with a Kramers Ground State. *Inorganic Chemistry* **47**, 10217–10219 (2008).
22. Katoh, K. *et al.* Multiple-decker phthalocyaninato dinuclear lanthanoid(iii) single-molecule magnets with dual-magnetic relaxation processes. *Dalton Transactions* **41**, 13582 (2012).
23. Kan, J. *et al.* Sandwich-Type Mixed Tetrapyrrole Rare-Earth Triple-Decker Compounds. Effect of the Coordination Geometry on the Single-Molecule-Magnet Nature. *Inorganic Chemistry* **52**, 8505–8510 (2013).
24. Wang, K. *et al.* Binuclear Phthalocyanine-Based Sandwich-Type Rare Earth Complexes: Unprecedented Two π-Bridged Biradical-Metal Integrated SMMs. *Chemistry - A European Journal* **19**, 11162–11166 (2013).
25. Cao, W. *et al.* Rational enhancement of the energy barrier of bis(tetrapyrrole) dysprosium SMMs via replacing atom of porphyrin core. *Chemical Science* **6**, 5947–5954 (2015).
26. Giménez-Agulló, N. *et al.* Single-Molecule-Magnet Behavior in the Family of $[Ln(OETAP)_2]$ Double-Decker Complexes (Ln=Lanthanide, OETAP=Octa(ethyl)tetraazaporphyrin). *Chemistry - A European Journal* **20**, 12817–12825 (2014).
27. Clemente-Juan, J. M., Coronado, E. & Gaita-Ariño, A. Magnetic polyoxometalates: from molecular magnetism to molecular spintronics and quantum computing. *Chem. Soc. Rev.* **41**, 7464–7478 (2012).
28. Biswas, S. *et al.* Mononuclear Lanthanide Complexes: Energy-Barrier Enhancement by Ligand Substitution in Field-Induced Dy^{III} SIMs. *Inorganic Chemistry* **56**, 7985–7997 (2017).
29. Hamada, D. *et al.* Luminescent Dy^{III} single ion magnets with same N_6O_3 donor atoms but different donor atom arrangements, \`fac'-$[Dy^{III}(HLDL\text{-}ala)_3]$·8H2O and \`mer'-$[Dy^{III}(HLDL\text{-}phe)_3]$·7H2O. *Polyhedron* **109**, 120–128 (2016).
30. Ren, M. *et al.* Lanthanide salen-type complexes exhibiting single ion magnet and photoluminescent properties. *Dalton Transactions* **45**, 2974–2982 (2016).
31. Harriman, K. L. M. & Murugesu, M. An Organolanthanide Building Block Approach to Single-Molecule Magnets. *Acc. Chem. Res.* **49**, 1158–1167 (2016).
32. Jiang, S.-D., Wang, B.-W., Sun, H.-L., Wang, Z.-M. & Gao, S. An Organometallic Single-Ion Magnet. *J. Am. Chem. Soc.* **133**, 4730–4733 (2011).
33. Evans, P., Reta, D., Whitehead, G. F. S., Chilton, N. F. & Mills, D. P. Bis-Monophospholyl Dysprosium Cation Showing Magnetic Hysteresis at 48 K. *Journal of the American Chemical Society* **141**, 19935–19940 (2019).
34. Le Roy, J. J. *et al.* An Organometallic Building Block Approach To Produce a Multidecker 4f Single-Molecule Magnet. *J. Am. Chem. Soc.* **135**, 3502–3510 (2013).
35. Prytula-Kurkunova, A. Y., Pichon, C., Duhayon, C., Amirkhanov, V. M. & Sutter, J.-P. Mononuclear Lanthanide Complexes Containing [O-O]-Chelating Sulfonylamidophosphate Type Ligands. *European Journal of Inorganic Chemistry* **2019**, 4592–4596 (2019).
36. Demir, S., Jeon, I.-R., Long, J. R. & Harris, T. D. Radical ligand-containing single-molecule magnets. *Coord. Chem. Rev.* **289–290**, 149–176 (2015).
37. Huang, G. *et al.* Strong Magnetic Coupling and Single-Molecule-Magnet Behavior in Lanthanide-TEMPO Radical Chains. *Inorg. Chem.* **57**, 11044–11057 (2018).
38. Watanabe, A., Yamashita, A., Nakano, M., Yamamura, T. & Kajiwara, T. Multi-Path Magnetic Relaxation of Mono-Dysprosium(III) Single-Molecule Magnet with Extremely

High Barrier. *Chem. Eur. J.* **17**, 7428–7432 (2011).
39. Meihaus, K. R. *et al.* Influence of an Inner-Sphere K^+ Ion on the Magnetic Behavior of N_2^{3-} Radical-Bridged Dilanthanide Complexes Isolated Using an External Magnetic Field. *Inorg. Chem.* **53**, 3099–3107 (2014).
40. Costes, J. P. *et al.* Analysis of the Role of Peripheral Ligands Coordinated to Zn^{II} in Enhancing the Energy Barrier in Luminescent Linear Trinuclear Zn-Dy-Zn Single-Molecule Magnets. *Chem.: Eur. J.* **21**, 15785–15796 (2015).
41. Hu, K.-Q. *et al.* A trimetallic strategy towards $Zn^{II}_4Dy^{III}_2Cr^{III}_2$ and $Zn^{II}_4Dy^{III}_2Co^{III}_2$ single-ion magnets. *Dalton Trans.* **44**, 15413–15416 (2015).
42. Yamashita, A. *et al.* Wheel-Shaped ErIIIZnII3 Single-Molecule Magnet: A Macrocyclic Approach to Designing Magnetic Anisotropy. *Angew. Chem. Int. Ed.* **50**, 4016–4019 (2011).
43. Feltham, H. L. C. *et al.* A Non-sandwiched Macrocyclic Monolanthanide Single-Molecule Magnet: The Key Role of Axiality. *Chem.: Eur. J.* **17**, 4362–4365 (2011).
44. Cucinotta, G. *et al.* Magnetic Anisotropy in a Dysprosium/DOTA Single-Molecule Magnet: Beyond Simple Magneto-Structural Correlations. *Angew. Chem. Int. Ed.* **51**, 1606–1610 (2012).
45. Boulon, M.-E. *et al.* Magnetic Anisotropy and Spin-Parity Effect Along the Series of Lanthanide Complexes with DOTA. *Angewandte Chemie International Edition* **52**, 350–354 (2012).
46. Liu, J.-L. *et al.* Switching the anisotropy barrier of a single-ion magnet by symmetry change from quasi-D_{5h} to quasi-O_h. *Chem. Sci.* **4**, 3310–3316 (2013).
47. Palacios, M. A. *et al.* Bifunctional $Zn^{II}Ln^{III}$ Dinuclear Complexes Combining Field Induced SMM Behavior and Luminescence: Enhanced NIR Lanthanide Emission by 9-Anthracene Carboxylate Bridging Ligands. *Inorg. Chem.* **53**, 1465–1474 (2014).
48. Jiang, Z. *et al.* Excess axial electrostatic repulsion as a criterion for pentagonal bipyramidal Dy^{III} single-ion magnets with high U_{eff} and T_B. *Journal of Materials Chemistry C* **6**, 4273–4280 (2018).
49. Liu, S.-S. *et al.* Half-Sandwich Complexes of Dy^{III}: A Janus-Motif with Facile Tunability of Magnetism. *Inorganic Chemistry* **54**, 5162–5168 (2015).
50. Wu, J. *et al.* Cis–trans isomerism modulates the magnetic relaxation of dysprosium single-molecule magnets. *Chem. Sci.* **7**, 3632–3639 (2016).
51. König, S. N. *et al.* Fast magnetic relaxation in an octahedral dysprosium tetramethyl-aluminate complex. *Dalton Trans.* **43**, 3035–3038 (2014).
52. Blagg, R. J. *et al.* Magnetic relaxation pathways in lanthanide single-molecule magnets. *Nature Chemistry* **5**, 673–678 (2013).
53. Zhang, P. *et al.* Equatorially Coordinated Lanthanide Single Ion Magnets. *J. Am. Chem. Soc.* **136**, 4484–4487 (2014).
54. Gifi, Albert. *Nonlinear Multivariate Analysis*. (Wiley, 1991).
55. Mair, P. & Leeuw, J. D. homals: Gifi methods for optimal scaling. (2021).
56. Dray, S. *et al.* ade4: Analysis of ecological data: exploratory and euclidean methods in environmental sciences. (2020).
57. Husson, F., Josse, J., Le, S. & Mazet, J. FactoMineR: Multivariate exploratory data analysis and data mining. (2020).
58. Akaike, H. A new look at the statistical model identification. *IEEE Trans. Atomat. Contr.* **19**, 716–723 (1974).
59. R Core Team. *R: A language and environment for statistical computing*. (R Foundation for Statistical Computing, 2022).
60. Ripley, B. & Venables, W. nnet: Feed-forward neural networks and multinomial log-linear models. (2021).
61. Ding, Y.-S. *et al.* A Study of Magnetic Relaxation in Dysprosium(III) Single-Molecule Magnets. *Chem. Eur. J.* **26**, 5893–5902 (2020).
62. Abragam, A. & Bleaney, B. *Electron Paramagnetic Resonance of Transition Ions*. (Oxford University Press, 2012).
63. Kassambara, A. & Mundt, F. factoextra: Extract and visualize the results of multivariate

data analyses. (2020).
64. Scheie, A. PyCrystalField: software for calculation, analysis and fitting of crystal electric field Hamiltonians. *J Appl Cryst* **54**, 356–362 (2021).
65. Hall, S. R., Allen, F. H. & Brown, I. D. The crystallographic information file (CIF): a new standard archive file for crystallography. *Acta Cryst A* **47**, 655–685 (1991).
66. Zabrodsky, H., Peleg, S. & Avnir, D. Continuous symmetry measures. *J. Am. Chem. Soc.* **114**, 7843–7851 (1992).
67. Alvarez, S. *et al.* Shape maps and polyhedral interconversion paths in transition metal chemistry. *Coordination Chemistry Reviews* **249**, 1693–1708 (2005).
68. Gu, L. & Wu, R. Origin of the anomalously low Raman exponents in single molecule magnets. *Phys. Rev. B* **103**, 014401 (2021).
69. Singh, A. & Shrivastava, K. N. Optical-acoustic two-phonon relaxation in spin systems. *physica status solidi (b)* **95**, 273–277 (1979).
70. Schloerke, B. *et al.* GGally: Extension to 'ggplot2'. (2021).
