# Supplementary material for: Data-driven design of molecular nanomagnets
Source: Nat Commun. 2022 Dec 9;13:7626. doi: 10.1038/s41467-022-35336-9 (PMC9734471; doi:10.1038/s41467-022-35336-9)
Supplement: Supplementary file 3 — Description of Additional Supplementary Files [file 41467_2022_35336_MOESM3_ESM.pdf]

Description of Additional Supplementary Files

Supplementary Data 1: This file contains the SIMDavis dataset.

Supplementary Software: contains all the files required to run the SIMDAVIS App locally, provided R-base is installed. It is a copy of the files in the bitbucket repo in https://bitbucket.org/rosaleny/simdavis/. It also contains brief instructions to use or install the SIMDAVIS app (SIMDAVIS Guide.pdf).

.
