# Supplementary material for: Data-driven design of molecular nanomagnets
Source: Nat Commun. 2022 Dec 9;13:7626. doi: 10.1038/s41467-022-35336-9 (PMC9734471; doi:10.1038/s41467-022-35336-9)
Supplement: Supplementary file 5 — Supplementary Software [file 41467_2022_35336_MOESM5_ESM.zip › SupplementarySoftware/simdavis/authors.html]

### Authors & Contributions

**Lorena E. Rosaleny**, ICMol (Universitat de València), Spain [rosaleny@uv.es]   
Conceived and programmed the dashboard-style interactive web application for data visualisation and analysis, double-checked the raw data, cleaned and organized the raw data into a tidy dataset, conceived and supervised the statistical analytical exploration, and also employed software to extract structural data.

**Yan Duan**, Spin-X Institute South China University of Technology, P. R. China [yanduan@uv.es]   
Designed the whole procedure for raw data extraction and classification, did the manual data mining, double-checked the raw data.

**Joana T. Coutinho**, CDRSP—Centre for Rapid and Sustainable Product Development (Polytechnic Institute of Leiria), Portugal [joana.t.coutinho@ipleiria.pt]   
Designed the whole procedure for raw data extraction and classification, did the manual data-mining, double-checked the raw data.

**Alejandro Gaita-Ariño**, ICMol (Universitat de València), Spain [gaita@uv.es]   
Proposed the SIMs study, did manual data-mining, double-checked the raw data, cleaned and organized the raw data into a tidy datase, and finally conceived and supervised the statistical data analysis.

**Salvador Cardona-Serra**, ICMol (Universitat de València), Spain [salvador.cardona@uv.es]   
Proposed the SIMs study, did manual data mining and double-checked raw data.

**Silvia Giménez-Santamarina**, ICMol (Universitat de València), Spain [silvia.m.gimenez@uv.es]   
Did manual data mining, double-checked raw data and implemented & employed software to extract structural data.

**Allen Scheie**, Neutron Scattering Division Oak Ridge National Laboratory, USA [scheieao@ornl.gov]   
Implemented & employed software to extract structural data.

**José J. Baldoví**, ICMol (Universitat de València), Spain [j.jaime.baldovi@uv.es]   
Proposed the SIMs study, did manual data mining.

  
  

### Acknowledgmements

This work has been supported by the COST Action MolSpin on Molecular Spintronics (Project 15128), H2020 (FATMOLS project) and QUANTERA (SUMO project), the European Research Council (ERC) under the European Union's Horizon 2020 research and innovation programme (grant agreement No 647301 for CoG “DECRESIM”, No 78822 for AdG “MOL2D”), the Spanish MINECO (grants MAT2017-89993-R, CTQ2017-89528-P, PID2020-117264GB-I00 and PID2020-117177GB-I00 cofinanced by FEDER and Excellence Unit María de Maeztu MDM-2015-0538 and CEX2019-000919-M), the Fundação para a Ciência e a Tecnologia (projects UIDB/04044/2020 and UIDP/04044/2020), and the Generalitat Valenciana (Prometeo Program of Excellence/2019/066, CDEIGENT/2019/022 and CIDEGENT/2021/018). This research used resources at the Spallation Neutron Source, a DOE Office of Science User Facility operated by the Oak Ridge National Laboratory.
The statistical analysis was performed by Raquel Gavidia Josa with the Statistical Section of the S.C.S.I.E. (Universitat de València). Pablo García Muñoz contributed to the SIMDAVIS app code.
  
