# Supplementary material for: Data-driven design of molecular nanomagnets
Source: Nat Commun. 2022 Dec 9;13:7626. doi: 10.1038/s41467-022-35336-9 (PMC9734471; doi:10.1038/s41467-022-35336-9)
Supplement: Supplementary file 5 — Supplementary Software [file 41467_2022_35336_MOESM5_ESM.zip › SupplementarySoftware/simdavis/changelog.html]

### Changelog

**Update (July 22nd, 2022) to version 1.1.9**  
Released SIMDAVIS version 1.1.9.

**Update (July 14th, 2022) to version 1.1.8.9006**  
Fixed one sample data, display closest\_polyhedron with 45º in boxplots, reordered levels of closest\_polyhedron and removed minor grid marks for axes (log10).

**Update (July 5th, 2022) to version 1.1.8.9005**  
Workaround for bug in annotation\_logticks, removed discrete variable pyCF\_coordination\_number from plots, changed C\_Raman and n\_Raman to C and n, removed data from pyCrystalField, updated variables\_verbose.txt.

**Update (July 1st, 2022) to version 1.1.8.9004**  
Fixed some data in dataset, added CCDC\_COD\_ID, link\_to\_CIF and CIF\_type variables, added pyCrystalField and SHAPE calculations and calculated slope\_CSM and axial\_distorsion.

**Update (February 14th, 2022) to version 1.1.8.9003**  
Added new variables available for plots (C\_Raman, n\_Raman, tau\_qtm) and updated About SIMDAVIS \> Variables tab with the newest additions.

**Update (February 2nd, 2022) to version 1.1.8.9002**  
Added new data calculated via pyCrystalField and SHAPE, new variables available for plots (closest\_polyhedron, main\_MJ, main\_MJ\_module), partial revision of CIF assignments to samples and link to CIFs. Added relaxation data (C\_Raman, n\_Raman, tau\_qtm).

**Update (August 03, 2021) to version 1.1.8.9001**  
Added filters to columns in View Data tab and added data calculated via pyCrystalField and SHAPE.

**Update (July 26, 2021) to version 1.1.8**  
Added meta tags to make SIMDAVIS App searchable.

**Update (July 12, 2021) to version 1.1.7**  
Added record T\_hyst samples from 2018, year also as numerical variable, descriptions of plots with number of annotated samples, download buttons for the data feeding each plot, only present categories are shown in z checkbox, and updated Acknowledgements.

**Update (May 3, 2021) to version 1.1.6**  
Fixed some chemical family assignments.

**Update (April 19, 2021) to version 1.1.5**  
Fixed some X''\_max data, updated README, and made the bitbucket repository public.

**Update (April 16, 2021) to version 1.1.4**  
Fixed some Hyst data, added AGPL-3.0 license tab and updated README.md

**Update (April 13, 2021) to version 1.1.3**  
Added new clustering with more categories for download, assigned "Unassigned" to NA in mag\_struct\_cluster, removed unused levels in coord. number and number of ligands, created different dataset for download with raw URLs, added descriptions for the clustering variables in the Variables tab, and fixed some errors.

**Update (March 29, 2021) to version 1.1.2**  
Added the updated molecular and magnetostructural clustering data, and renamed chemical families.

**Update (March 26, 2021) to version 1.1.1**  
A new home tab and new subtabs in "About SIMDAVIS" tab (Authors, Feedback&Bugs, Changelog) added.

**Update (March 22, 2021) to version 1.1**  
Added CIF links and missing magnetic data from 2016, fixed errors in T\_B3/T\_B3H values when field was present, and some other minor corrections.

**Update (March 18, 2021) to version 1.0.3**  
DOI opens in new window, removed values H = 0, and some corrections.

**Update (March 16, 2021) to version 1.0.2**  
Histogram to bar chart, variable names and factors changed.

**Update (March 11, 2021) to version 1.0.1**  
Fixed some compound names, the chemical families of some samples were reassigned, preselected variables in data tab changed and a tab about the SIMDAVIS app is added.

**Update (March 8, 2021) to version 1.0**  
Fixed one doi error and family categories.  
  
Update (December 3, 2019) to version 0.9

Update (July 26, 2019) to version 0.8

Update (July 8, 2019) to version 0.7

Update (July 2, 2019) to version 0.6

Update (June 26, 2019) to version 0.5

Update (June 21, 2019) to version 0.4

Update (June 14, 2019) to version 0.3

Update (June 6, 2019) to version 0.2

Update (May 31, 2019) to version 0.1

  
  
