# Supplementary material for: Data-driven design of molecular nanomagnets
Source: Nat Commun. 2022 Dec 9;13:7626. doi: 10.1038/s41467-022-35336-9 (PMC9734471; doi:10.1038/s41467-022-35336-9)
Supplement: Supplementary file 5 — Supplementary Software [file 41467_2022_35336_MOESM5_ESM.zip › SupplementarySoftware/simdavis/home.html]

### SIMDAVIS App

**SIMDAVIS Version 1.1.9**  
*Last Updated July 22nd, 2022*


---

SIMDAVIS (for Single Ion Magnet DAta VISualisation) is a dashboard-style web application, designed to fulfill a double objective. First, it is a dataset of Lanthanide-based Single Ion Magnets, which as of July 2022 contains 1411 samples from 448 articles published between 2003 and 2019. The dataset can be downloaded via the **Data Tab** > Download Data. SIMDAVIS is also a visualisation tool, allowing the production of scatter plots, box plots and bar charts, which then can be downloaded as vectorial PDF files. You can also download the represented subset of the data as a tsv file.

In the **ScatterPlots tab** the user can represent 15 quantitative physical properties versus one another; the chemical category (plus publication year) of every data point is identified by a color. For each of the 12 qualitative categorization possibilities, each category may be shown or hidden; checking a box adds a linear regression for each category. Click on any data point in the plot to show its sample ID, compound and DOI hyperlink in the table below the plot.

The **BoxPlots tab** allows to examine the distribution of each SIMs quantitative property vs a categorization criterion. The **BarCharts tab** explores the frequency of different qualitative variables in our dataset. The **Data tab** to browse the dataset allows choosing the data columns to show, ordering in ascending or descending order, and filtering by arbitrary keywords.

SIMDAVIS was specifically developed to accompany a publication, and we kindly request its citation if you use it. The most recent version is the following: Duan, Y., *et al*. Data mining, dashboard and statistical analysis: a powerful framework for the chemical design of molecular nanomagnets. Preprint at https://arxiv.org/abs/2103.03199 (2021).
