# Supplementary material for: Data-driven design of molecular nanomagnets
Source: Nat Commun. 2022 Dec 9;13:7626. doi: 10.1038/s41467-022-35336-9 (PMC9734471; doi:10.1038/s41467-022-35336-9)
Supplement: Supplementary file 5 — Supplementary Software [file 41467_2022_35336_MOESM5_ESM.zip › SupplementarySoftware/SIMDAVIS Guide.pdf]

1. System requirements

SIMDAVIS can be run either from a web browser (simpler, recommended) or locally (if you want to customize the code and/or dataset).

The web browser version just requires a web browser and accessing the SIMDAVIS internet site:
https://rosaleny.shinyapps.io/simdavis_dashboard/

It has no specific requirements in terms of browser, operating system or screen size (from cellphone to desktop computer). It has been tested and found to work properly, among others, on:
·Firefox 88.0.1 (64-bit) on Windows 10 Home and Windows 10 Enterprise
·Google Chrome 91.0.4472.77 (64-bit) on Windows 10 Home
·Internet Explorer 11 on Windows 7
·Opera 75 on Windows 7
·Google Chrome 90.0.4430.212 (x86_64) on MacOS Mojave 10.14
·Safari 14.0.3 on MacOS BigSur 11.2.3
·Firefox 102.0 (64-bit) on Ubuntu 20.04
·Firefox 88.0 (64-bit) on Ubuntu 16.04
·Firefox 88.0.1 (64-bit) on Ubuntu 20.04
·Firefox 78.3.0esr (64-bit) on Debian linux 9.13 (64-bit)
·Chromium 73.0.3683.75 on Debian linux 9.13 (64-bit)
·Google Chrome 90.0.4430.210 on Android 7.1.1 and Android 10
·Qwant 4.0.4 on Android 7.1.1

2. Installation guide

The web browser version requires no installation.

The local version has been tested on R version 4.1.3 under Ubuntu 20.04.1 LTS. If you wish to run a local installation of SIMDAVIS you will need to install R (https://www.r-project.org/) and the following R packages (and their dependencies): readr, dplyr, shiny, ggplot2 and DT.

The installation of R and all required packages requires familiarity with R language and about 1 h on a typical desktop computer. Once this is installed, SIMDAVIS does not require installation as it is executed within R locally. It however requires download of the SIMDAVIS folder from the bitbucket repository.

The bitbucket repository at https://bitbucket.org/rosaleny/simdavis/src/issue-6/ contains the Free Libre Open-Source Software (FLOSS) that is the basis for the SIMDAVIS App, where you can download all files required for this app.

3. Demo – Visualizing the SIMDAVIS dataset

The recommended procedure is employing the web version at:
https://rosaleny.shinyapps.io/simdavis_dashboard/
Loading the app can take between 2 and 10 s on a typical device (from cellphone to desktop computer). Initially you should land on the “Home” tab, containing basic information on how the rest of the tabs work. The dataset is already loaded, and can be visualized in different ways in each of the tabs. Response to any specific action (plotting data, change tabs, download data) can take between 1 and 5 s. A more detailed explanation of what data visualizations you can expect from this app can be found in Supplementary Section 3.

4. Instructions for use – Visualizing your own dataset

The SIMDAVIS app has been developed and extensively tested using Rstudio IDE 2022.02.3+492 "Prairie Trillium" Release. If you choose to install Rstudio Desktop (https://www.rstudio.com/products/rstudio/download/) you can run the SIMDAVIS app employing Rstudio’s GUI. After starting Rstudio, go to File > Open Project > go to the simdavis folder. Then you go to File > Open File > app.R. You will see in the editor pane the app.R file, and to run the script you need to click on the ‘Run App’ icon above the file editor. This will open your internet browser with the app being executed.

You can also run the app.R script without installing Rstudio IDE by opening a terminal or console window, going to the simdavis folder, and executing this:

```
R -e "shiny::runApp('app.R')"
```

Note that this does start the server part of the application, not the client side. As runApp starts the application on a randomly selected port, you will need to go to your browser and paste the port you get on the last line your console prompts. For instance if you get “Listening on http://127.0.0.1:3868”, you can copy http://127.0.0.1:3868 on the URL bar on your browser, and you will be running the app on your browser.

NOTE: If run locally, changing the dataset involves substituting the data file “SIMDAVIS_data_2022_07_14.rds” with your own. Note that this file is an R native format, and you will need to generate it from within R (https://www.rdocumentation.org/packages/base/versions/3.6.2/topics/readRDS). For instance with a functions such as:

```
saveRDS(dataframe, file = “filename.rds”)
```
