# Supplementary figures and images for: Data-driven design of molecular nanomagnets
Source: Nat Commun. 2022 Dec 9;13:7626. doi: 10.1038/s41467-022-35336-9 (PMC9734471; doi:10.1038/s41467-022-35336-9)

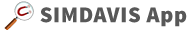

Supplement: Supplementary file 5 — Supplementary Software [file 41467_2022_35336_MOESM5_ESM.zip › SupplementarySoftware/simdavis/www/SIMDAVIS_App_sign.png]

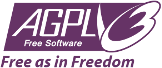

Supplement: Supplementary file 5 — Supplementary Software [file 41467_2022_35336_MOESM5_ESM.zip › SupplementarySoftware/simdavis/www/agplv3-with-text-162x68.png]

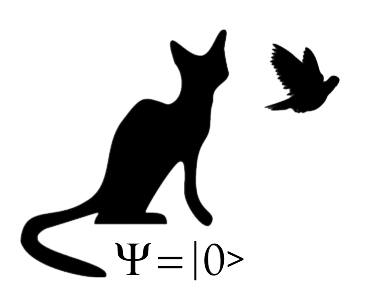

Supplement: Supplementary file 5 — Supplementary Software [file 41467_2022_35336_MOESM5_ESM.zip › SupplementarySoftware/simdavis/www/cat2.png]

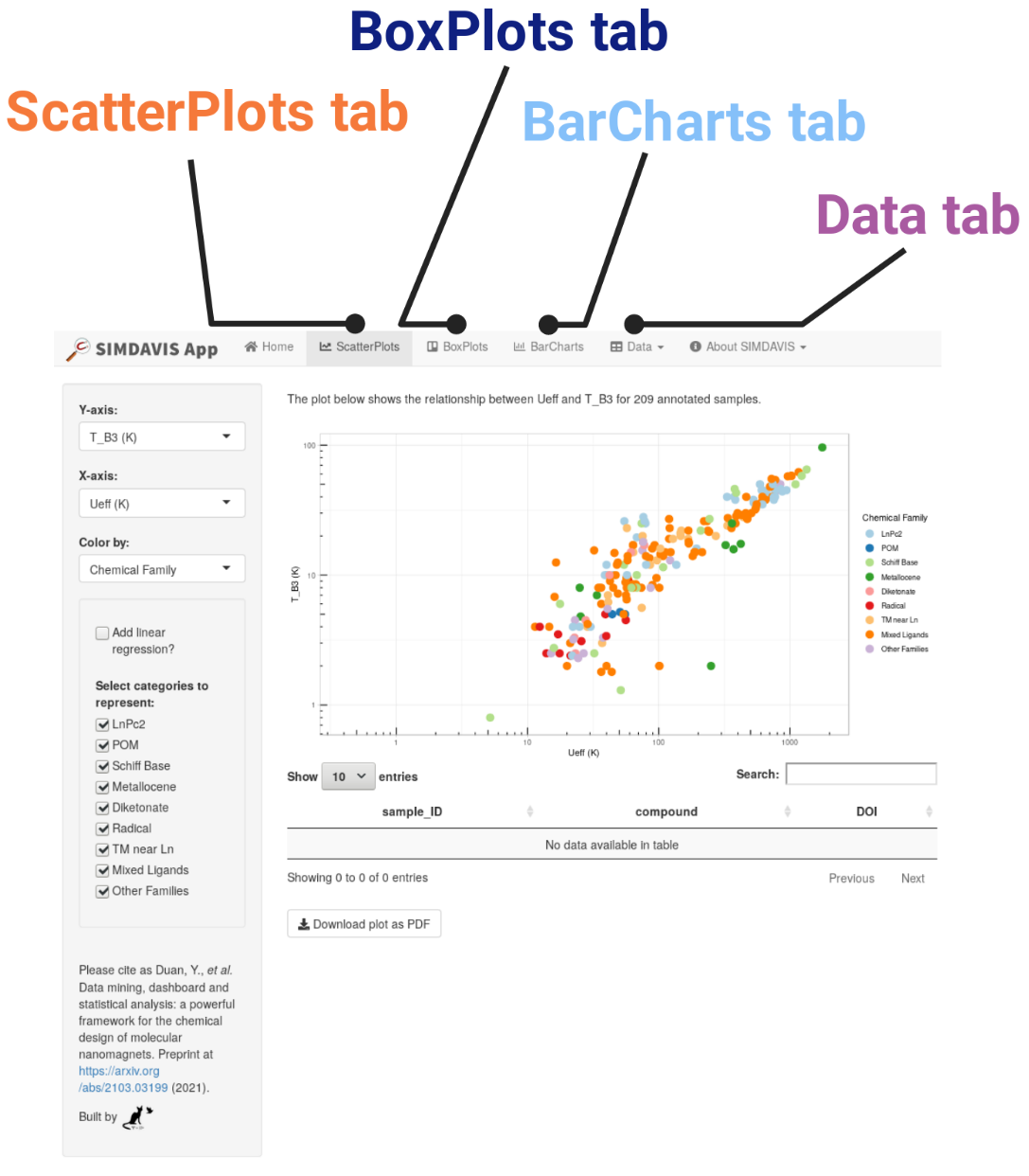

Supplement: Supplementary file 5 — Supplementary Software [file 41467_2022_35336_MOESM5_ESM.zip › SupplementarySoftware/simdavis/www/fig_Home_SIMDAVIS.png]

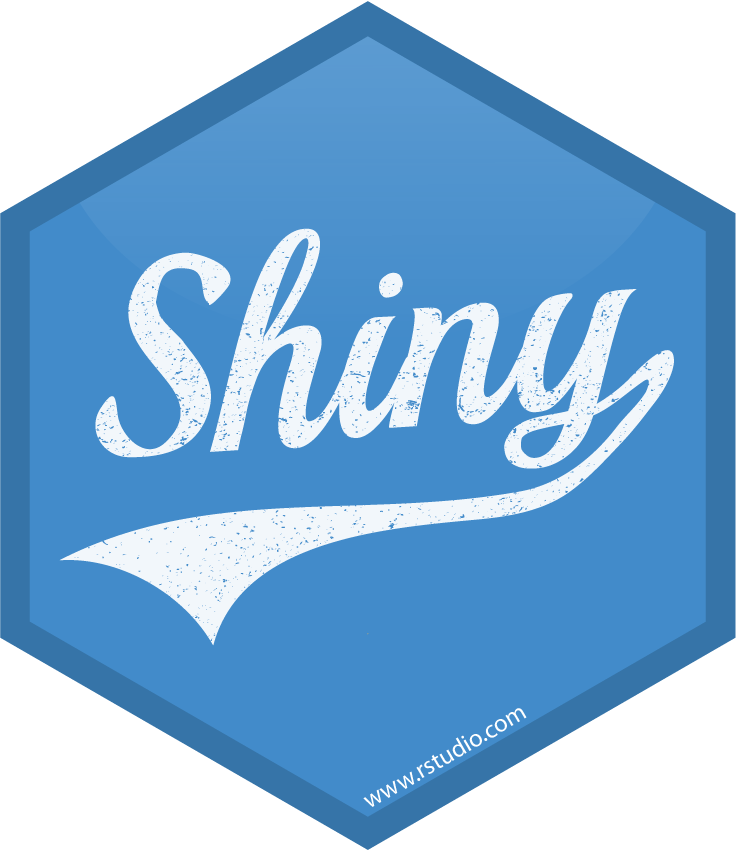

Supplement: Supplementary file 5 — Supplementary Software [file 41467_2022_35336_MOESM5_ESM.zip › SupplementarySoftware/simdavis/www/shiny.png]

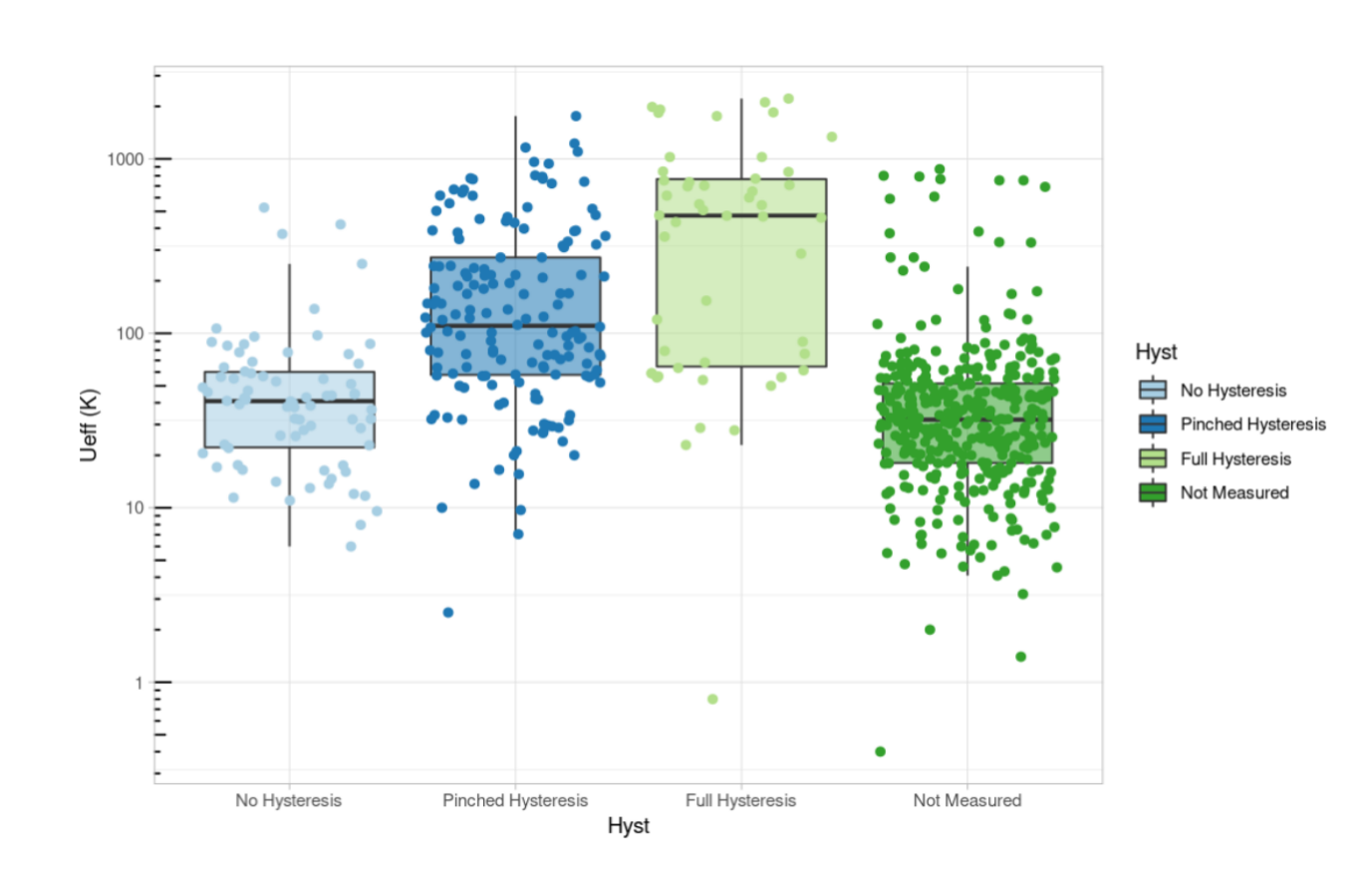

Supplement: Supplementary file 5 — Supplementary Software [file 41467_2022_35336_MOESM5_ESM.zip › SupplementarySoftware/simdavis/www/SIMDAVIS_image.png]
